# Non-invasive prehabilitation to foster widespread fMRI cortical reorganization before brain tumor surgery: lessons from a case series

**DOI:** 10.1007/s11060-024-04774-4

**Published:** 2024-07-23

**Authors:** Leonardo Boccuni, Alba Roca-Ventura, Edgar Buloz-Osorio, David Leno-Colorado, Selma Delgado-Gallén, María Cabello-Toscano, Ruben Perellón-Alfonso, Gloria Villalba-Martínez, Francisco Martínez-Ricarte, Jesús Martín-Fernández, Mònica Buxeda-Rodriguez, Gerardo Conesa-Bertrán, Mireia Illueca-Moreno, Estela Lladó-Carbó, Cristóbal Perla y Perla, César Garrido, José Carlos Pariente, Carlos Laredo, Emma Muñoz-Moreno, Núria Bargalló, Carlo Trompetto, Lucio Marinelli, David Bartrés-Faz, Kilian Abellaneda-Pérez, Alvaro Pascual-Leone, Josep María Tormos-Muñoz

**Affiliations:** 1 Institut Guttmann, Institut Universitari de Neurorehabilitació adscrit a la UAB, Badalona, Barcelona, Spain; 2 Universitat Autònoma de Barcelona, Cerdanyola del Vallès, Bellaterra, Spain; 3 Fundació Institut d’Investigació en Ciències de la Salut Germans Trias i Pujol, Badalona, Barcelona, Spain; 4 Departament de Medicina, Facultat de Medicina i Ciències de la Salut, Institut de Neurociències, Universitat de Barcelona, Barcelona, Spain; 5 Institut d’Investigacions Biomèdiques August Pi i Sunyer (IDIBAPS), Barcelona, Spain; 6 Department of Neurosurgery, Hospital del Mar, Barcelona, Spain; 7 Systems Neurologic and Neurotherapeutic Group at Research Institute Hospital del Mar, Barcelona, Spain; 8 Universitat Pompeu Fabra, Barcelona, Spain; 9 Department of Neurosurgery, Vall d’Hebron Hospital, Universitat Autònoma de Barcelona, Barcelona, Spain; 10 Department of Neurosurgery, Hôpital Gui de Chauliac, Montpellier, France; 11 Department of Neurosurgery, Hospital Universitario Nuestra Señora de Candelaria, Tenerife, Spain; 12 Universidad de La Laguna, Tenerife, Spain; 13 Department of Neurosurgery, University Hospital Mútua Terrassa, Barcelona, Spain; 14 Department of Neurosurgery, Teknon Medical Center, Barcelona, Spain; 15 Department of Neurosurgery, HM Nou Delfos, Barcelona, Spain; 16 Magnetic Resonance Image Core Facility (IDIBAPS), Barcelona, Spain; 17 Neuroradiology Section, Radiology Department, Diagnostic Image Centre, Hospital Clinic of Barcelona, University of Barcelona, Barcelona, Spain; 18 Centro de Investigación Biomédica en Red de Salud Mental (CIBERSAM), Instituto de Salud Carlos III, Barcelona, Spain; 19 Department of Neuroscience, Rehabilitation, Ophthalmology, Genetics, Maternal and Child Health, University of Genova, Genova, Italy; 20 IRCCS Ospedale Policlinico San Martino, Genova, Italy; 21 Wolk Center for Memory Health and Marcus Institute for Aging Research, Hebrew Senior Life, Boston, MA USA; 22 Department of Neurology, Harvard Medical School, Boston, MA USA; 23 Centro de Investigación Traslacional San Alberto Magno, Universidad Católica de Valencia San Vicente Mártir, Valencia, Spain

**Keywords:** Brain tumor, Prehabilitation, Neurorehabilitation, Neuromodulation, Neurosurgery, Case series.

## Abstract

**Purpose:**

The objective of this prospective, single-centre case series was to investigate feasibility, clinical outcomes, and neural correlates of non-invasive Neuromodulation-Induced Cortical Prehabilitation (NICP) before brain tumor surgery. Previous studies have shown that gross total resection is paramount to increase life expectancy but is counterbalanced by the need of preserving critical functional areas. NICP aims at expanding functional margins for extensive tumor resection without functional sequelae. Invasive NICP (intracranial neuromodulation) was effective but characterized by elevated costs and high rate of adverse events. Non-invasive NICP (transcranial neuromodulation) may represent a more feasible alternative. Nonetheless, up to this point, non-invasive NICP has been examined in only two case reports, yielding inconclusive findings.

**Methods:**

Treatment sessions consisted of non-invasive neuromodulation, to transiently deactivate critical areas adjacent to the lesion, coupled with intensive functional training, to activate alternative nodes within the same functional network. Patients were evaluated pre-NICP, post-NICP, and at follow-up post-surgery.

**Results:**

Ten patients performed the intervention. Feasibility criteria were met (retention, adherence, safety, and patient’s satisfaction). Clinical outcomes showed overall stability and improvements in motor and executive function from pre- to post-NICP, and at follow-up. Relevant plasticity changes (increase in the distance between tumor and critical area) were observed when the neuromodulation target was guided by functional neuroimaging data.

**Conclusion:**

This is the first case series demonstrating feasibility of non-invasive NICP. Neural correlates indicate that neuroimaging-guided target selection may represent a valid strategy to leverage neuroplastic changes before neurosurgery. Further investigations are needed to confirm such preliminary findings.

**Supplementary Information:**

The online version contains supplementary material available at 10.1007/s11060-024-04774-4.

## Introduction

Despite considerable advancements in neurosurgery, radiotherapy and chemotherapy, brain tumors are still characterized by elevated mortality and morbidity rates. Brain tumors are among the first three leading causes of cancer death for children, adolescents, and adults younger than 50, with an overall five-year survival rate of 33% [1]. Furthermore, brain tumors cause morbidity and elevated burden for the healthcare system, responsible for 7,7 millions of disability-adjusted life-years worldwide between 1990 and 2016 [2]. Gross total resection increase survival and delay disease progression [3–5]; however, the delicate balance between tumor exeresis and preserving healthy brain tissue poses cost-benefit dilemmas [6, 7]. Radical approaches may achieve supratotal resection but at the expense of functional sequelae, while subtotal resection may preserve critical areas but heightens the risk of tumor recurrence.

A potential strategy to overcome this dilemma is neuromodulation-induced cortical prehabilitation (NICP) that might be able to boost the transfer of functional activity from eloquent areas (close to the tumor) to other nodes within the same functional network but located at a greater distance from the tumor [8]. To date, neuromodulation in the form of brain stimulation is the application of a targeted electrical stimulus to the cortex to modulate neural activity either in an excitatory or inhibitory fashion, with invasive or non-invasive modalities, for assessment and treatment purposes [9]. Invasive neuromodulation typically involves surgical implantation of epidural, subdural or parenchymal electrodes connected to a pulse generator device, whereas non-invasive neuromodulation typically involves the delivery of an electric or magnetic stimulus over the scalp corresponding to a specific cortical region. The most extensively investigated form of brain stimulation, transcranial magnetic stimulation, has demonstrated considerable therapeutic potential and reasonable safety for several neurological and psychiatric disorders [10]. Each NICP session involves inhibitory neuromodulation, to transiently deactivate eloquent areas, coupled with intensive functional training, to foster the activation of alternative brain resources. Clinically, gradual cortical reorganization cannot produce any negative impact on cognitive and motor functions related to the targeted brain areas [10]. At the same time, the cumulative effect of repeated sessions aims at increasing the distance between the tumor and the pattern of cortical activity, thus widening the functional margins for safe, gross total resection [11].

NICP is a pioneering field, with only six patients receiving invasive neuromodulation and two patients receiving non-invasive neuromodulation [11–15]. As reviewed in detail by Hamer et al., invasive NICP yielded relevant neuroplastic changes in terms of fMRI related to functions at risk of being compromised [11]. However, the technique’s invasiveness led to elevated costs (a first surgery to implant intracranial electrodes, a second surgery to remove the electrodes and the tumor) and adverse events (infections, hematomas, seizures) [13, 14]. For instance, a patient with anaplastic astrocytoma at the level of left inferior frontal gyrus and presenting with left dominant speech fMRI activity within tumor showed the appearance of new language activation clusters at ipsilesional and contralesional hemisphere, but experienced seizures and osteo-myelitis of bone flap during NICP intervention [13]. By contrast, non-invasive NICP was safe (i.e. no adverse events) but showed modest results in terms of neuroplastic adaptations as evidenced by functional magnetic resonance imaging [12, 15]. For instance, Barcia et al. reported the case of a patient with oligodendroglioma adjacent to left inferior frontal gyrus who received NICP in the form of transcranial magnetic stimulation coupled with speech therapy. Results of task-based fMRI at the end of the intervention were substantially unchanged as compared to baseline, with a persistent peritumoral left dominant speech activation cluster [12].

Notably, structured task practice elicit itself neuroplastic changes given that adequate schedule, dosis, specificity, variability, progressive difficulty, and feedback is provided [16]. Indeed, aside from neuromodulation modalities a striking difference of previous NICP studies is the amount of training. For invasive NICP, Rivera-Rivera et al. provided several hours of formal (therapist-assisted) training, and additional hours of self-administered training every day for 2–3 weeks [13]; similarly, Serrano-Castro et al. provided at least six hours of speech training every day for six consecutive days [14]. Conversely for non-invasive NICP, Barcia et al. applied 10 min of speech training immediately after each neuromodulation session, and Dadario et al. did not provide any formal training [12, 15]. Such heterogeneity complicates the interpretation of findings and warrant further investigation.

The present case series aims to move a step forward from previous case reports, by investigating non-invasive NICP before neurosurgery to gather consistent outcomes from patients receiving the same intervention. By delivering neuromodulation and functional training with achievable specificity and intensity, hypotheses were that the intervention would have been feasible (adherence, absence of adverse events, patients’ satisfaction) and capable of producing relevant neuroplastic changes (widening of functional margins).

## Methods

For the present case series, the methodology corresponds to the protocol of a subsequent phase I, pilot feasibility trial (ClinicalTrials.gov, identifier NCT05844605), which is still ongoing. Full protocol description has been already published [17]; therefore, this section only summarizes core methodological elements. To ensure replicability of all study procedures, further details are also available as supplementary material.

This prospective single-center consecutive case series adhered to the Declaration of Helsinki, with approval from the Research Ethical Committee of Fundació Unió Catalana d’Hospitals (approval number: CEI 21/65, version 1, 13/07/2021). Participants provided written informed consent before joining the study.

### Settings, timeframes, participants

Clinical assessments, neurophysiological examinations, and all NICP interventions were performed at the Guttmann Institute (Guttmann Barcelona– Brain Health and Neurorehabilitation, Barcelona, Spain). Neuroimaging assessments were conducted at the Unitat d’Imatge per Ressonància Magnètica IDIBAPS (Institut d’Investigacions Biomèdiques August Pi i Sunyer) at Hospital Clínic de Barcelona, Barcelona. Surgeries were performed at hospitals in Barcelona metropolitan area (Hospital del Mar, Vall d’Hebron Hospital, Hospital Universitario Mútua Terrassa, HM Nou Delfos). Recruitment spanned July 2021 to March 2023, with assessments conducted at baseline (pre-NICP), post-intervention but before neurosurgery (post-NICP), and follow-up (three to six months post-surgery). The intervention, lasting two to four weeks, comprised daily neuromodulation and intensive functional training. Neurosurgery was performed soon after.

Adults requiring neurosurgery for any brain tumor were deemed eligible, with exclusion criteria including contraindications to imaging or transcranial magnetic stimulation (TMS), unstable medical conditions, substance abuse history, and severe musculoskeletal or cognitive disorders impacting the intervention. Participants needed to comprehend the study’s purpose, provide written consent, and agree on attending a minimum of 10 treatment sessions.

### Assessments

Comprehensive clinical, neurophysiological, and neuroimaging assessments were conducted at each time point (for detailed description please refer to the manual as supplementary material and to the previously published protocol [17]). Clinical assessments evaluating neurological status, functional independence, quality of life and motor function were performed by a physiotherapist (LB), while assessments of cognitive functions were performed by neuropsychologists (ARV and DLC). Neurophysiological evaluations consisted of neuronavigated TMS to identify the cortical site of the primary motor cortex (M1 hotspot) eliciting the largest motor response from the first dorsal interosseus, and its resting motor threshold (RMT). For treatment, the intensity of low-frequency repetitive TMS was set at 90% RMT of the affected hemisphere. Neuroimaging assessments included structural and task-based functional magnetic resonance imaging (fMRI). For fMRI, key paradigms were the finger tapping task (tap each finger with the thumb), semantic decision task (mention objects from certain places), and word generation task (mention words starting from a certain letter).

Baseline assessments guided the identification of the function at highest risk of being compromised, i.e. the function judged as most vulnerable to compromission due to surgical tumor eradication. To this end, presence of eloquent areas identified with fMRI was the main parameter to consider. In cases where fMRI were not suitable or available, other factors were considered such as presenting symptoms and neuroanatomical considerations based on tumor location. Normalized coordinates (MNI space) of the target for neuromodulation were determined either as M1 hotspot (in case of eloquent areas related to upper limb motor function), peak-activation of fMRI cluster (in case of language/cognitive function, or in case where M1 hotspot could not be determined), or based on previous literature individuating normalized neuroanatomical sites (in case where the area related to the function at risk of being compromised was not identifiable with TMS nor fMRI paradigms).

### Intervention

Participants were scheduled to attend in between 10 and 20 sessions of treatment, provided once or twice a day, consisting of inhibitory neuromodulation coupled with intensive task training. Neuromodulation consisted of TMS or multichannel Transcranial Direct Current Stimulation (tDCS), depending on whether the target was a specific spot or a broader region, respectively. Intensive task training was performed for one hour, immediately after (TMS) or during (tDCS) neuromodulation; it consisted of practicing motor or cognitive tasks related to the function at risk of being compromised. The goal of neuromodulation was to provoke a virtual lesion, i.e. a temporary inhibition of the targeted eloquent area (a functionally active area close to the tumor) [18]. The goal of intensive training was to foster the activation of alternative nodes within the same functional network, thus reducing the functional relevance of the eloquent area.

### Feasibility

Feasibility was defined by retention, adherence, safety, and patient’s satisfaction, as for the published trial protocol [17]. Retention was met if at least 75% of patients completed the intervention. For each patient, completion was achieved if at least 10 sessions and at least 75% of planned sessions (adherence) were performed. Safety was defined as the absence of serious adverse events causally related to the intervention, and patient’s satisfaction was based on the PATSAT questionnaire [19].

### Interpretation of neuroplastic changes

Neuroplasticity was determined based on structural and fMRI data. Regarding structure, we determined tumor volume and centre of gravity coordinates (tumor-CoG). Regarding function, we determined fMRI activation clusters of the function at risk of being compromised. By analysing task-fMRI data, the cluster with the highest significance value of the peak of activation (T-statistics) was considered the *main* cluster of interest [20]. The derived variables included the total volume of fMRI clusters, the volume of fMRI clusters only on the affected hemisphere, the volume of the main fMRI cluster, and coordinates of peak-fMRI activity within the main cluster (main-peak). For the target of neuromodulation (N-target), the only variables were its coordinates. All imaging data were normalized to Montreal Neuroimaging Institute (MNI) space before processing.

Analysis of volumes (mm^3^) were performed using an in-house Matlab script. To delineate tumors, semi-automatic segmentation was conducted with a software application (ITK-SNAP) [21], while fMRI-based activation clusters were automatically generated by fMRI processing leading to activation maps associated to each task (for a detailed description of the methodology please refer to the published protocol) [17]. Further analyses determined total fMRI activation volume, as the sum of volumes for all significant clusters; lateralization index, as the ratio between the volume of the clusters on the affected hemisphere over total fMRI activation volume (multiplied by 100); and relevance index, as the ratio between the volume of the main significant cluster over total fMRI activation volume (multiplied by 100).

Analysis of distances (mm) measured the squared Euclidean distance between main-peak detected by task-based fMRI and two points determined at baseline: N-target and tumor-CoG [22]. By comparing task-based fMRI at baseline versus at the end of the prehabilitation protocol (right before neurosurgery), an increase in distance from N-target was interpreted as positive outcome research-wise (i.e. the intervention was capable of inducing neuroplastic changes), while an increase in distance from tumor-CoG was interpreted as positive outcome clinical-wise (neuroplastic changes were relevant for neurosurgical planning and outcomes). MRIcroGL, a cross-platform NIfTI format image viewer (https://www.nitrc.org/projects/mricrogl) was used for MRI visualization [23]. Supplementary Fig. 1 depicts a representation of analysis by volumes and distances.

### Statistical analysis

Non-parametric statistics reported measures of central tendency and dispersion (median, interquartile range), differences between paired observations (Wilcoxon signed-rank test), and correlations (Kendall’s tau). Given the small sample size and intervention heterogeneity, the emphasis was on informative case descriptions rather than firm conclusions.

The case series adheres to the PROCESS Guideline [24].

## Results

10 cases were consecutively enrolled (six males and four females, age range 29–64, median 55). Five cases had a risk of compromission for upper limb motor function and were therefore classified as ‘motor patients’, while other five cases presented with symptoms related to speech production and other high-order cognitive functions, and were classified as ‘cognitive patients’. To ease the interpretation of findings, cases one to five and cases six to ten represent motor and cognitive patients, respectively.

**Fig. 1:**
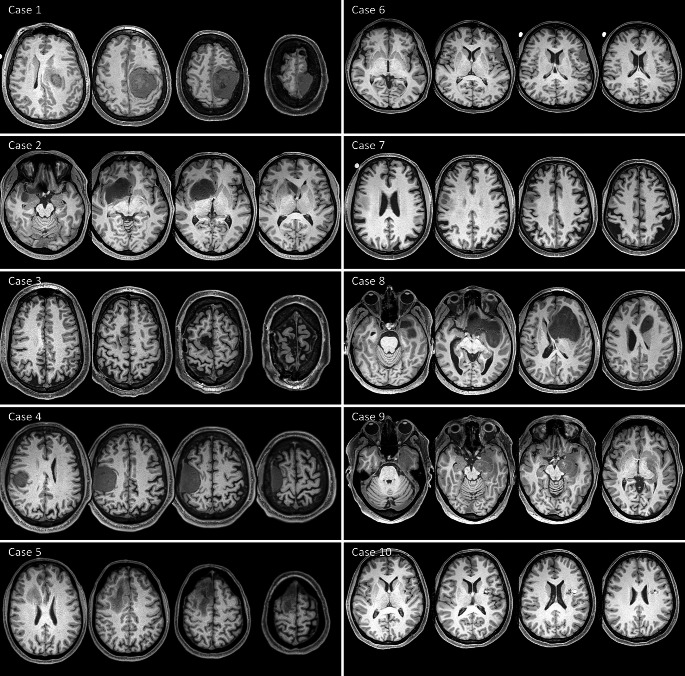
Tumor distribution at baseline. Axial slices of the structural MRI for each subject (native space) depicting tumor location. The first five cases (left side) are ‘motor patients’, the second five cases (right side) are ‘cognitive patients’

Table 1 reports tumor classification and WHO grading, symptoms at baseline, and feasibility outcomes. Figure 1 shows axial slices of the structural MRI of each subject (native space) to illustrate tumor distribution at baseline. Eight patients had tumor infiltrating the frontal lobe in isolation or with other lobes (parietal, temporal); case 9 had a tumor infiltrating the hippocampus and case 10 had the mass at the level of the insula. Six patients (case 2, 3, 5, 7, 8, 9) had a glioma, three patients (case 1, 4, 6) a meningioma, and case 10 a cavernoma. Concentration and focus alterations were reportedly the most common symptoms at baseline, both for motor (case 2, 3, 5) and cognitive (case 7, 9) patients. Only two patients presented with upper limb motor symptoms at baseline: case 1, with moderate upper limb hypertonia and motor impairment; and case 6, with slight hand dexterity deficits. Other symptoms at baseline included hand paraesthesia (case 4), speech disturbances (case 6) and severe memory loss (case 9).

**Table 1:** Tumor characteristics, symptoms at baseline, and feasibility outcomes

	Case 1	Case 2	Case 3	Case 4	Case 5	Case 6	Case 7	Case 8	Case 9	Case 10	Median (IQR)
**Tumor classification and grading**											
Tumor side	left	right	right	right	right	left	right	left	left	left	
Tumor location (Presurgical diagnosis)	frontoparietal cortex	intraparenchymal fronto-insulo-temporal area	cortico-subcortical parasagittal superior frontal area	frontoparietal cortex	cortico-subcortical infiltrative frontal premotor area	inferior (opercular) frontal cortex	cortico-subcortical middle frontal area	intraparenchymal fronto-temporal-insular area	cortico-subcortical hippocampal area	intraparenchymal subinsular area	
Tumor classification (WHO 2021)	Meningioma	Glioma	Glioma	Meningioma	Oligodendroglioma	Meningioma	Glioblastoma	Astrocytoma	Glioblastoma	Cavernoma	
Grade	WHO grade I	WHO grade III	WHO grade III	WHO grade II	WHO grade II	WHO grade I	WHO grade IV	WHO grade IV	WHO Grade IV		
**Baseline symptoms**											
Initial symptoms leading to diagnosis	Moderate speech disturbance / Moderate-severe right hand motor and sensory impairment	Dizziness / Presyncope	Comitial crisis	Left hand sensory impairment (paresthesia)	Comitial crisis	Slight speech disturbance (paraphasia) / Right handclumsiness and reduced grip	Transitory left facial supranuclear paralysis / Involuntary movements / Speech disturbance	Comitial crisis	Comitial crisis	Right arm and leg paresthesias. Previous left hemisphere stroke (2018) (ad integrum recovery)	
Worst symptom at baseline	Right arm monoparesia	Slight concentration and focus alteration	Slight concentration and focus alteration	Left hand paresthesias	Slight concentration and focus alteration	Slight speech disturbances and right hand motor impairment	Moderate concentration and focus alteration. Fatigue. Facial palsys	Absence seizures	Severe memory loss. Sustained attention deficit	Asymptomatic	
Functions at risk of being compromized	Upper limb motor function	Upper limb motor function, executive function	Upper limb motor function	Upper limb motor function	Upper limb motor function, executive function	Speech production, dexterity	Speech production, motor control facial muscles	Speech production	Memory	Speech production, motor control facial muscles	
**Feasibility**											
Days in between first MRI and surgery	18	35	26	52	18	30	16	36	44	25	28 (18)
Total days NICP (1–2 sessions performed)	6	15	6	10	8	20	6	12	21	10	10 (8)
Sessions performed for NICP (perf-NICP)	7	22	11	10	13	20	6	20	21	17	15 (10)
Sessions planned for NICP (plan-NICP)	10	26	11	11	16	20	10	20	20	20	18 (9)
Adherence (perf-NICP/plan-NICP)	70	85	100	91	81	100	60	100	105	85	88 (18)
Extra sessions before surgery	1	0	4	9	0	0	1	0	0	0	0 (1)
Total sessions performed (NICP + extra)	8	22	15	19	13	20	7	20	21	17	18 (7)
Adverse events not attributable to NICP	None	None	None	None	None	None	None	None	Motor symptoms	None	
Adverse events attributable to NICP	None	None	None	None	None	None	None	None	None	headache	
Patient’s satisfaction (IN-PATSAT32)	99	99	99	99	89	99	61	99	80	99	99 (8)

All feasibility criteria were met. Two patients (case 1 and 7) couldn’t achieve the bare minimum of 10 NICP sessions, due to time constraints (only 6 weekdays available for the intervention). The same patients were also below the bare minimum of 80% treatment adherence. Therefore, retention was adequate, with eight patients out of 10 performing at least 10 sessions, and with at least 80% adherence to planned sessions. The only adverse event attributable to the intervention was a transient headache for case 10. Another adverse event (not attributable to the intervention) was the onset of motor symptoms for case 9, with the appearance of upper limb motor impairments and balance deficits. This case was initially diagnosed as low-grade glioma at the level of the left hippocampus; unfortunately, it later resulted being a grade IV glioblastoma infiltrating the midbrain and the corticospinal tract, which explained the sudden onset and rapid progression of motor symptoms. Given that the intervention focused on cognitive function (memory training), and that the N-target targeted the left supramarginal gyrus (a cortical area related to the hippocampal network [25], it was excluded a causal relationship with the intervention. Finally, patient’s satisfaction was overall excellent, with seven patients reporting the maximal score on the IN-PATSAT32 questionnaire.

Clinical outcomes are reported in Table 2. When comparing results for the whole cohort at different time points, several significant differences were found, indicating an improvement from baseline that peaked post-NICP for the 6-minute walk test, processing speed, executive function, and memory. Compared to baseline, choice reaction time task was the only outcome that slightly worsened at follow-up, though there was a trend for improvement post-NICP, compared to baseline.

**Table 2:** Clinical outcomes

	whole cohort (*n* = 10)			patients receiving motor NICP (*n* = 5)			patients receiving cognitive NICP (*n* = 5)		
	pre-NICP	post-NICP	follow-up	pre-NICP	post-NICP	follow-up	pre-NICP	post-NICP	follow-up
**Overall functional status**									
NANO scale (neurological symptoms)	0 (1)	0 (1)	1 (1)	0 (2)	0 (1)	0 (1)	0 (1)	0 (1)	1 (2)
KPS (independency)	90 (8)	90 (15)	90 (3)	90 (10)	90 (10)	90 (10)	90 (0)	90 (10)	90 (0)
EORTC QLQ C30 (functioning)	84 (21)	87 (18)	93 (16)	93 (29)	93 (13)	96 (4)	82 (4)	72 (27)	82 (7)
EORTC QLQ C30 (symptoms)	10 (13)	10 (23)	12 (18)	3 (13)	3 (8)	0 (10)	15 (10)	19 (14)	18 (3)
EORTC QLQ C30 (quality of life)	71 (27)	83 (25)	83 (19)	75 (33)	83 (17)	83 (0)	67 (8)	71 (27)	58 (17)
EORTC BN20 (impact of symptoms)	14 (20)	13 (17)	9 (16)	7 (17)	5 (13)	2 (5)	15 (13)	19 (12)	13 (7)
EORTC FA12 (fatigue)	19 (30)	8 (36)	6 (21)	6 (25)	8 (8)	0 (3)	19 (14)	28 (27)	17 (12)
**Clinical assessment**,** motor function**									
Grip strength (kg)	30 (8)	30 (10)	30 (15)	29 (9)	30 (17)	33 (15)	32 (6)	31 (9)	30 (10)
9-Hole Peg Test (seconds)	21 (9)	20 (16)	25 (5)	21 (8)	20 (22)	25 (3)	22 (9)	22 (10)	22 (11)
Reaction time task, simple (milliseconds)	298 (25)	294 (46)	307 (43)	305 (50)	301 (67)	305 (28)	295 (21)	294 (33)	309 (46)
Reaction time task, choice (milliseconds)	587 (40)	567 (100)	**600 (167)¨**	591 (21)	543 (82)	580 (32)	568 (56)	589 (91)	895 (298)
6-MWT (meters)	578 (64)	607 (67)	**589 (95)¨**	574 (39)	607 (40)	588 (90)	602 (60)	588 (93)	590 (56)
Forward Reach, standing (cm)	38 (5)	37 (3)	30 (13)	38 (5)	37 (2)	32 (12)	38 (5)	36 (4)	28 (9)
**Clinical assessment**,** cognitive function**									
WAIS IV number-letters (working memory)	11 (2)	10 (2)	10 (2)	9 (1)	10 (2)	10 (1)	11 (1)	11 (2)	11 (1)
WAIS IV - inverse digits (working memory)	4 (0)	5 (1)	4 (1)	4 (0)	4.5 (1)	4.5 (1)	4 (1)	5 (1)	4 (0)
WAIS IV number-key (processing speed)	56 (20)	67 (3)*	63 (13)	56 (9)	66 (5)	67 (8)	58 (13)	68 (1)	57 (6)
WAIS IV blocks (visoconstruction, executive function)	34 (8)	47 (16)*	**41 (7)¨**	34 (9)	50 (11)	44 (9)	34 (6)	37 (8)	31 (7)
RVLT (memory) - total learning	47 (17)	65 (16)*	**59 (11)^**	48 (15)	58 (18)	59 (5)	48 (10)	68 (7)	52 (8)
RVLT - Recognition	15 (3)	15 (0)	15 (0)	15 (1)	15 (0)	15 (0)	15 (5)	15 (0)	14 (1)
RVLT - delay	12 (4)	14 (2)	13 (3)	12 (1)	14 (2)	13 (3)	12 (6)	14 (2)	12 (3)
PMR (phonemic fluency)	39 (26)	46 (18)	40 (17)	33 (30)	52 (21)	42 (21)	48 (15)	46 (12)	40 (7)
TB-R (verbo-verbal denomination)	6 (0)	6 (0)	6 (0)	6 (0)	6 (0)	6 (0)	6 (0)	6 (0)	6 (0)
TB-R (reading comprehension)	8 (0)	8 (0)	8 (0)	8 (0)	8 (0)	8 (0)	8 (0)	8 (0)	8 (0)
TMT-A (visual attention, sequencing)	35 (9)	28 (4)	35 (7)	33 (12)	28 (1)	31 (8)	36 (3)	34 (7)	37 (1)
TMT-B (visual attention, sequencing, flexibility)	76 (12)	70 (15)	69 (12)	76 (8)	65 (11)	66 (3)	75 (20)	75 (11)	88 (8)
Hayling test time (behavioural regulation)	3 (1)	2 (1)	3 (1)	3 (0.1)	2 (0.3)	2 (1)	3 (1)	3 (3)	4 (1)

Wilcoxon signed-rank test (*p* <.05) when comparing:

*pre-NICP versus post-NICP

^pre-NICP versus follow-up

¨post-NICP versus follow-up

Neural correlates on rationale for neuromodulation, analysis by volumes, and analysis by distances are reported in Supplementary Table 1. The selection of target for neuromodulation (TMS) was: M1 hotspot for case 1, 3, 5; based on neuroanatomical considerations for case 6 and 7; based on peak activation of the clusters of interest detected by task-based fMRI for case 4, 8, 10. Notably, the peak-fMRI selected for case 4 and 8 was from the cluster showing the most significant activation (the main cluster); for case 10, the peak-fMRI of a secondary cluster was selected, because located in a region with overlapping clusters for motor and cognitive functions, in an attempt of targeting multiple functions of interest at the same time. Multichannel tDCS was applied in case 2 because the tumor was located subcortically and potentially affecting the white matter underlying several cortical areas, with three cathodes (F4, C4, P4) to inhibit a widespread parietofrontal region, and one anode (F3) on the contralesional hemisphere. For similar reasons case 8 received multichannel tDCS with three cathodes (F3, P3, T7) and one anode (C4); because of patient’s disposability to attend two sessions per day, the patient received TMS in the morning sessions and multichannel tDCS in the afternoon sessions.

**Fig. 2:**
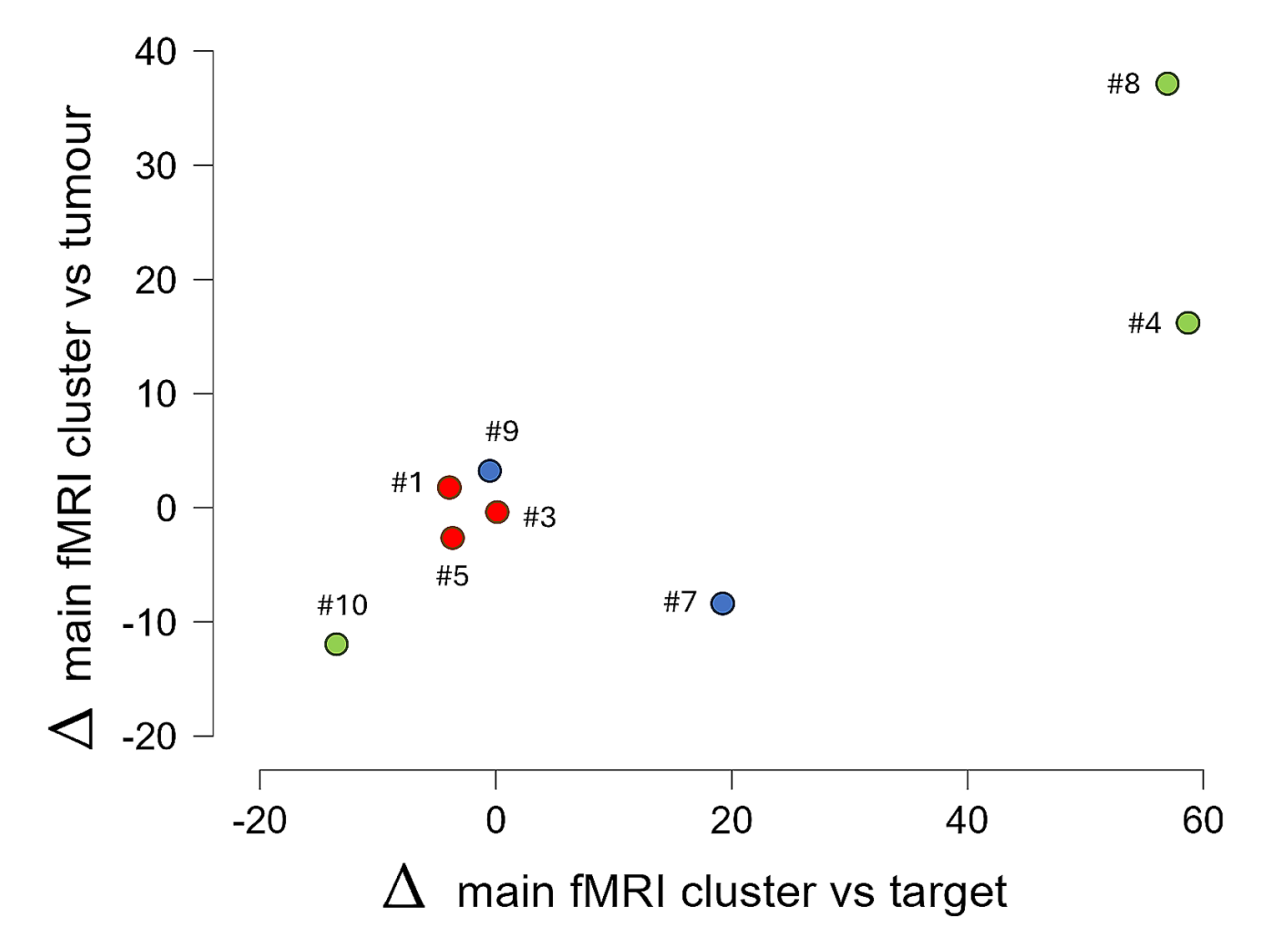
Change in distances between main-peak versus N-target (x-axis) and main-peak versus tumor-CoG (y-axis)

**Fig. 3:**
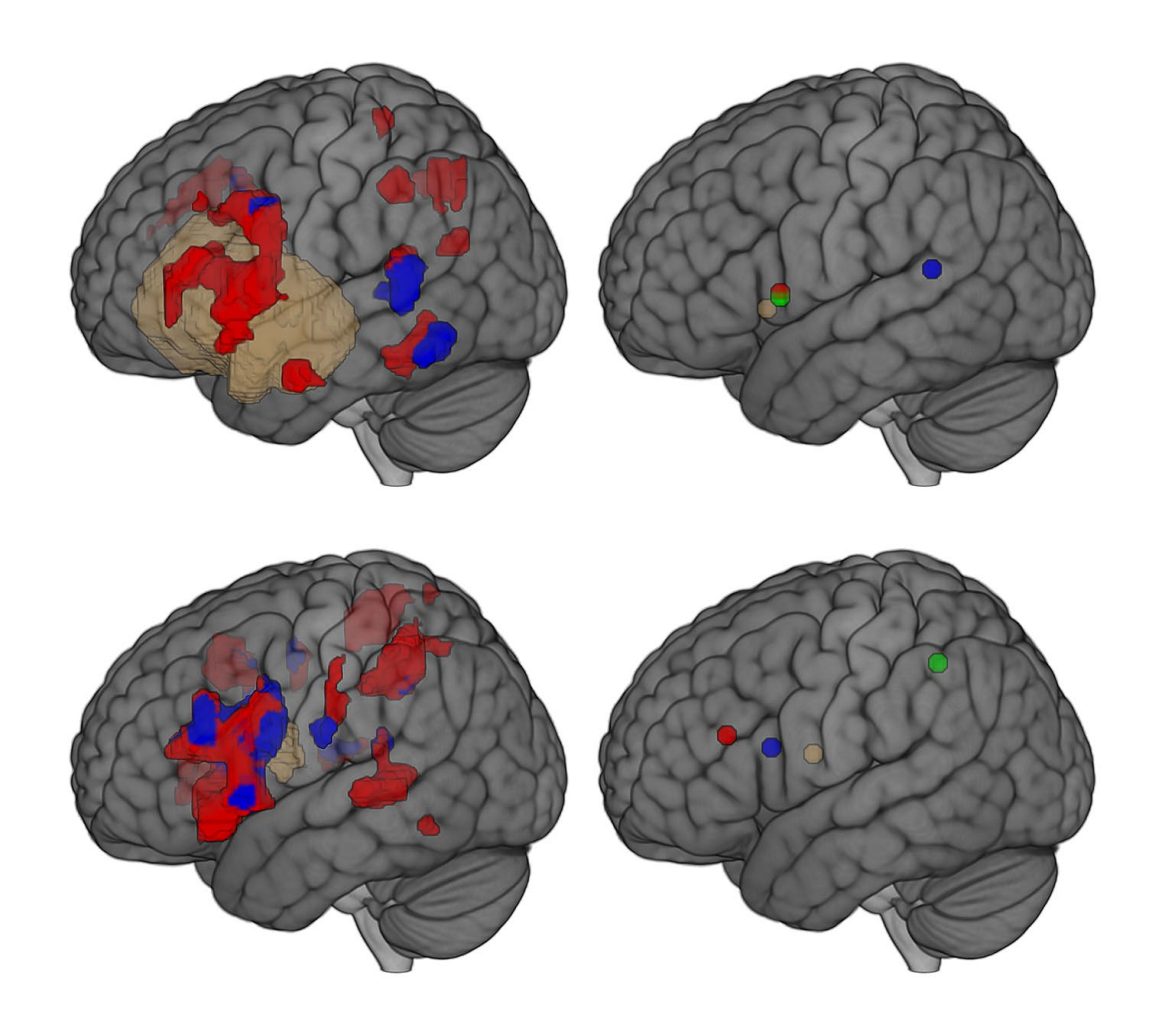
Neural correlates when targeting peak-fMRI. Case 8: (top images) and case 10 (bottom images) targeted neuromodulation at peak-fMRI of the main cluster of interest and at peak-fMRI of a secondary cluster, respectively. Change in volume distributions is shown on the left images, change in distances is shown on right images. Brown cluster: tumor; red clusters: fMRI activity pre-NICP; blue clusters: fMRI activity post-NICP; yellow dot: tumor-CoG; red dot: main-peak pre-NICP; blue dot: main-peak post-NICP; green dot: N-target

There was a trend for increase in distances, both when looking at the distance between main-peak versus N-target, and main-peak versus tumor-CoG. A closer look (Figs. 2 and 3) revealed different neuroplastic responses, potentially due to the rationale for N-target selection.

Figure 2 is a scatterplot for changes in distance between main-peak versus N-target (x-axis), and changes in distance between main-peak versus tumor-CoG (y-axis). Each dot is labelled with the corresponding case number. Green dots represent cases targeting the peak-activation of an fMRI cluster, with the distinction that case 4 and 8 focused on main-peak, whereas case 10 focused on peak-fMRI of a secondary cluster. Red dots represent cases targeting M1 hotspot (cases 1, 3, 5). Blue dots (case 7 and 9) represent cases targeting brain areas based on neuroanatomical considerations and/or literature references. Case 2 is not present in this scatterplot, because multichannel tDCS was applied instead of TMS; case 6 is also not included, because of unavailable fMRI data post-NICP.

Figure 3 illustrates two extreme cases of the series in terms of neuroplastic changes, the first one (case 8, upper quadrants) with positive outcomes and the second one (case 10) with rather negative outcomes. In fact, case 8 showed an important increase in the distances for both main-peak versus N-target, and for main-peak versus tumor-CoG. On the other hand, case 10 showed a decrease of both distances, moving main-peak even closer to the tumor. Both cases were ‘cognitive patients’ with the lesion affecting the left hemisphere. Notably, a diriment factor may be the rationale for N-target selection: for case 8, the target was in almost perfect correspondence with the main-peak fMRI of interest. For case 10, the target corresponded with the peak of a secondary fMRI cluster distant from both the tumor and the main cluster. At the level of N-target, both cases showed a local reduction of cortical activity, but for case 10 there was also an unwanted approximation of main-peak to tumor-CoG.

## Discussion

The present case series investigated feasibility and neural correlates of non-invasive NICP on 10 patients with brain tumor. Considering that only eight cases were previously published (two cases of non-invasive NICP and six cases of invasive NICP), the reported findings represent a significant advancement in the field. The desired outcome is, as result of the cumulative effect from several sessions of non-invasive neuromodulation coupled with intensive task training, to determine a reorganization of cortical activity away from the tumor site, thus favouring the odds of supratotal resection without functional sequelae. While the intervention was overall feasible, neural correlates varied greatly depending on neuromodulation target selection and inferences are here discussed.

### Feasibility

The most serious adverse event due to TMS is the occurrence of seizures [10]. Epilepsy is a common issue in patients with brain tumor, often occurring as initial symptom leading to diagnosis [26]. Therefore, stimulation parameters targeting peritumoral areas were carefully selected to ensure safety. There is consensus that low-frequency (≤ 1 Hz) repeated TMS (rTMS) at 90% intensity of resting motor threshold is associated with a very low likelihood of inducing seizure (approximately 1 out of 100,000 sessions) due to its inhibitory effect [10]. Indeed, low-frequency rTMS is evenly applied for the investigation and treatment of patients with drug-resistant epilepsy [27]. According to these premises, no serious adverse events attributable to neuromodulation were observed during the conduct of the present study.

Aside from *clinical* feasibility, the study demonstrated *organizational* feasibility, i.e. that it is possible to coordinate complex activities such as patient’s referral, initial clinical, neuroimaging, and neurophysiological assessments, 10 to 20 sessions of neuromodulation coupled with task intensive task training, post-intervention assessments, and the production of a presurgical report to the neurosurgeon right before surgery, in about two to four weeks (note: formal TMS mapping for neurosurgical planning was performed at the hospital where neurosurgery took place, independently from study procedures; presurgical report was intended as complementary information). This is the result of multidisciplinary collaboration of neurosurgeons, neurologists, physiotherapists, neuropsychologists, and neuroradiologists from distinct institutions; the developed methodology is described in the previously published protocol and as supplementary material of this article, to encourage the development of future research studies and the translation to clinical practice.

### Neural correlates

In the present case-series, relevant neuroplastic changes occurred when the low-frequency rTMS target was selected based on individual functional neuroimaging. Three patients received neuromodulation over a cortical site corresponding to the peak of activity for fMRI clusters identified at baseline. In all three cases, a specific fMRI task was selected because the main cluster of interest was close to the tumor. However, positive outcomes (increase of the distance between fMRI clusters and the tumor) resulted in only two cases (one ‘cognitive’ and one ‘motor’ patient); a common feature was neuromodulation applied over the main cluster of interest. For the third case, negative outcomes (decrease of the distance between fMRI clusters and the tumor) resulted from neuromodulation applied over a secondary cluster, which was also distant from the tumor. Therefore, functional neuroimaging-guided target selection may represent a promising strategy to elicit relevant changes, but the choice of the cluster should prioritize those closer to the tumor, to displace the pattern of functional activity away from the tumor (and surgical) site. Such findings are in line with previous studies showing that TMS delivered at individualized, functional neuroimaging-guided targets may optimize reliability and clinical effectiveness [28–30]. However, other three patients in this case series received individualized neuromodulation, this time based on M1-hotspot defined during TMS mapping yet did not show meaningful neuroplastic changes. Notably, a previous study compared the extent of functional connectivity of M1 as determined by fMRI (peak-activation of finger tapping task) versus TMS (M1 hotspot) [31]. Results showed stronger and more diffuse connectivity of M1 peak-fMRI (to premotor areas, basal ganglia, and insula) than M1 hotspot. Authors argued that TMS treatment targeting M1 peak-fMRI may result in larger network effects than targeting M1 hotspot [31]. While this inference may help interpreting the present findings, most of the literature currently consider M1 hotspot as gold standard for neuromodulation of motor function, and more research is needed to corroborate the superiority of targeting M1 peak-fMRI.

### Limitations

There are several limitations to be considered, the foremost being the small sample size requiring caution in the generalizability of the present findings, with future larger studies needed to confirm both feasibility and neural outcomes. Another limitation is the predominant use of task-based fMRI to interpret neuroplastic changes, without considering other neuroimaging modalities such as resting state fMRI that may have further contributed to the interpretation of multiple large-scale network dynamics, and perhaps the investigation of traditionally non-eloquent areas clinically relevant but often neglected [32]. This is particularly relevant for those cases where neither TMS mapping nor task-based fMRI were diriment for the identification of neuromodulation target. Again, future studies should aim at investigating multiple neuroimaging modalities not only to help the interpretation of neuroplastic changes, but also a priori to contribute to the decision-making process of which function (and underlying cortical area) to target with the intervention. An in-depth analysis of neuroimaging findings is fundamental in the neuro-oncological field, considering that between group comparison (intervention versus placebo) would not be feasible or even ethical. A further limitation, and suggestion for future research studies, was the lack of comparisons between presurgical neural correlates (neuroimaging, TMS mapping) versus cortical stimulation mapping during neurosurgery. Indeed, considering brain mapping during surgical procedure as gold standard, it would have been insightful to compare it with presurgical neuroimaging analysis to test the reliability and clinical relevance of neuroplastic changes. This was not possible due to technical and organizational limitations, but we strongly advise the registration of targets for cortical stimulation mapping in normalized space to perform such analyses. Finally, the number of sessions was arbitrarily based upon the days/weeks available from initial referral to the day of surgery. Future studies may include clinical assessments or neural correlates as biomarkers to determine when prehabilitation has reached the objective of neuroplastic change with widening of the distance between functional activation clusters and the tumor, thus setting the optimal timing for neurosurgery.

## Conclusion

In conclusion, results from this case series demonstrate that non-invasive prehabilitation is feasible and may produce relevant neuroplastic changes widening the distance between the tumor and eloquent areas. To reach this ambitious goal, multidisciplinary collaboration is fundamental to define a personalized, focused, and intensive intervention, both in terms of neuromodulation target and functional training.

## Electronic supplementary material

Below is the link to the electronic supplementary material.

Supplementary Material 1

## Data Availability

De-identified data are available upon request from the corresponding authors.
