# Supplementary material for: Non-invasive prehabilitation to foster widespread fMRI cortical reorganization before brain tumor surgery: lessons from a case series
Source: J Neurooncol. 2024 Jul 23;170(1):185–98. doi: 10.1007/s11060-024-04774-4 (PMC11447047; doi:10.1007/s11060-024-04774-4)
Supplement: Supplementary file 1 — Supplementary Material 1 [file 11060_2024_4774_MOESM1_ESM.pdf]

SUPPLEMENTARY MATERIAL

- Supplementary Figure 1
- Supplementary Table 1
- Study procedures (manual)

Title:

Non-invasive prehabilitation to foster widespread cortical reorganization before brain tumor surgery: lessons from a case series.

Authors

Leonardo Boccuni[1,2], Alba Roca-Ventura[1,3,4], Edgar Buloz-Osorio[1,2,3], David Leno-Colorado[1,2,3], Selma Delgado-Gallén[1,2,3], María Cabello-Toscano[4,5], Ruben Perellón-Alfonso[1,2,3,4,5], Gloria Villalba-Martínez[6,7,8], Francisco Martínez-Ricarte[9], Jesús Martín-Fernández[10,11,12], Mònica Buxeda-Rodriguez[13], Gerardo Conesa-Bertrán[14], Mireia Illueca-Moreno[15], Estela Lladó-Carbó[15], Cristóbal Perla y Perla[15], César Garrido[5,16,17], José Carlos Pariente[5,16], Carlos Laredo[5,16], Emma Muñoz-Moreno[5,16], Núria Bargalló[4,5,16,17,18], Carlo Trompetto[19,20], Lucio Marinelli[19,20], David Bartrés-Faz[1,4,5], Kilian Abellaneda-Pérez[1,2,3]†, Alvaro Pascual-Leone[1,21,22]†, Josep María Tormos Muñoz[1,23]†

Affiliations

1 Institut Guttmann, Institut Universitari de Neurorehabilitació adscrit a la UAB, Badalona, Barcelona, Spain.

2 Universitat Autònoma de Barcelona, Bellaterra, Cerdanyola del Vallès, Spain.

3 Fundació Institut d'Investigació en Ciències de la Salut Germans Trias i Pujol, Badalona, Barcelona, Spain.

4 Departament de Medicina, Facultat de Medicina i Ciències de la Salut, Institut de Neurociències, Universitat de Barcelona, Spain.

5 Institut d'Investigacions Biomèdiques August Pi i Sunyer (IDIBAPS), Barcelona, Spain.

6 Department of Neurosurgery, Hospital del Mar, Barcelona, Spain.

7 Systems Neurologic and Neurotherapeutic Group at Research Institute Hospital del Mar, Barcelona, Spain.

8 Universitat Pompeu Fabra, Barcelona, Spain.

9 Department of Neurosurgery, Vall d'Hebron Hospital, Universitat Autònoma de Barcelona, Barcelona, Spain.

10 Department of Neurosurgery, Hôpital Gui de Chauliac, Montpellier, France.

11 Department of Neurosurgery, Hospital Universitario Nuestra Señora de Candelaria, Tenerife, Spain.

12 Universidad de La Laguna, Tenerife, Spain.

13 Department of Neurosurgery, University Hospital Mútua Terrassa, Barcelona, Spain.

14 Department of Neurosurgery, Teknon Medical Center, Barcelona, Spain.

15 Department of Neurosurgery, HM Nou Delfos, Barcelona, Spain.

16 Magnetic Resonance Image Core Facility (IDIBAPS), Barcelona, Spain.

17 Neuroradiology Section, Radiology Department, Diagnostic Image Centre, Hospital Clinic of Barcelona, University of Barcelona, Barcelona, Spain.

18 Centro de Investigación Biomédica en Red de Salud Mental (CIBERSAM), Instituto de Salud Carlos III, Barcelona, Spain.

19 University of Genova, Department of Neuroscience, Rehabilitation, Ophthalmology, Genetics, Maternal and Child Health, Genova, Italy.

20 IRCCS Ospedale Policlinico San Martino, Genova, Italy.

21 Hinda and Arthur Marcus Institute for Aging Research and Deanna and Sidney Wolk Centre for Memory Health, Hebrew Senior Life, Boston, MA, USA.

22 Department of Neurology, Harvard Medical School, Boston, MA, USA.

23 Centro de Investigación Traslacional San Alberto Magno, Universidad Católica de Valencia San Vicente Mártir, Valencia, Spain.

† These authors contributed equally to this work.

Supplementary Figure 1. Analysis by volumes and distances.

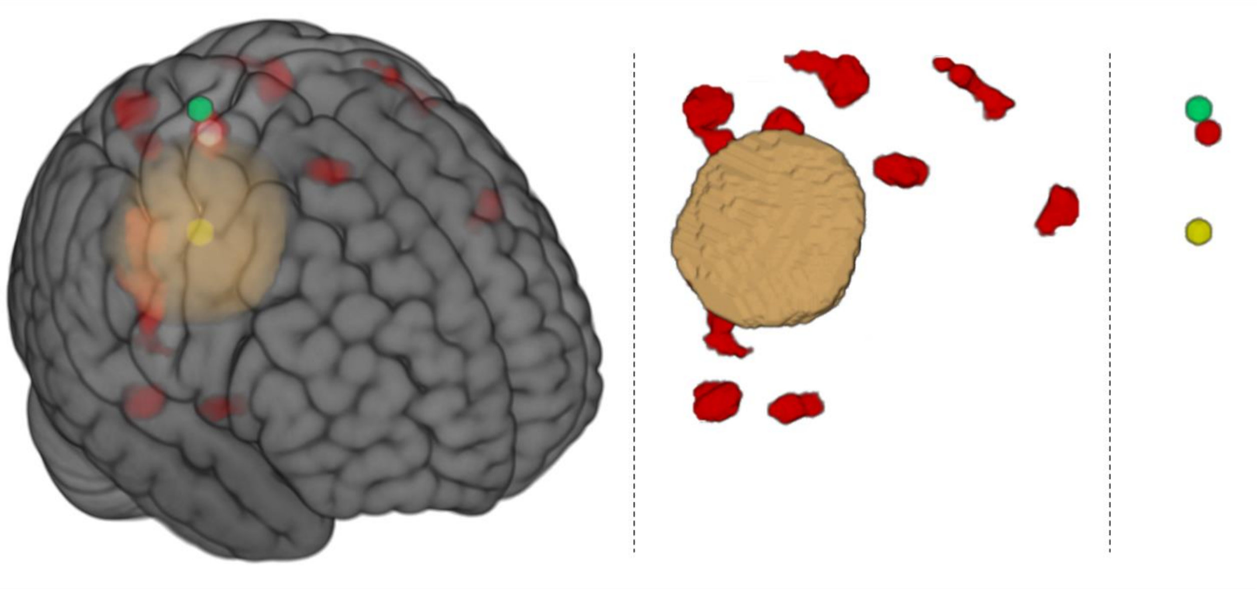

Normalized structural and functional MRI data were used to determine volumes of the tumor (brown), and of activation clusters (red) related to the function at risk of being compromised. Analysis of distances considered three points: tumor centre of gravity (yellow dot), neuromodulation target (green dot), and the peak-fMRI of the main cluster of interest (red dot).

Supplementary Table 1. Neural correlates.

	Case 1	Case 2	Case 3	Case 4	Case 5	Case 6	Case 7	Case 8	Case 9	Case 10	Median (IQR)
**Target for neuromodulation and main fMRI cluster**											
Focus of NICP (motor, cognitive)	motor	motor	motor	motor	Motor	cognitive	cognitive	cognitive	cognitive	cognitive	
fMRI of interest	Finger tapping task, right hand	Finger tapping task, left hand	Finger tapping task, left hand	Finger tapping task, left hand	Finger tapping task, left hand	Word generation task	Semantic decision task	Semantic decision task	Word generation task	Word generation task	
Neuromodulation (TMS/tDCS)	TMS	tDCS	TMS	TMS	TMS	TMS	TMS	TMS, tDCS	TMS	TMS	
Type of target	TMS hotspot	32-channel EEG electrodes	TMS hotspot	peak fMRI (main cluster)	TMS hotspot	Anatomical	Anatomical	peak fMRI (main cluster)	Resting state target (from literature)	peak fMRI (secondary cluster)	
Neural region targeted by neuromodulation	Inferior parietal lobule	Precentral gyrus	Precentral gyrus	Precentral gyrus	Precentral gyrus	Inferior frontal gyrus	Precentral gyrus	Inferior frontal gyrus	Supramarginal gyrus	Inferior parietal lobule	
Neural region of peak fMRI main cluster pre-NICP	Precentral gyrus	Precentral gyrus	Precentral gyrus	Precentral gyrus	Precentral gyrus	Middle temporal gyrus	Superior parietal lobule	Inferior frontal gyrus	Precentral gyrus	Inferior frontal gyrus	
Neural region of peak fMRI main cluster post-NICP	Precentral gyrus	Precentral gyrus	Precentral gyrus	Angular gyrus	Precentral gyrus	NA	Middle temporal gyrus	Superior temporal gyrus	Precentral gyrus	Inferior frontal gyrus	
**Analysis by volumes**											
tumor size (pre-NICP)	102744	39000	13048	51824	63592	7240	5096	133616	41984	2064	40492 (56352)
tumor size (post-NICP)	100704	33616	13768	52200	55128	7432	7928	138048	57104	2043	42908

											(49 176)
total fMRI volume (pre-NICP)	1956 48	1684 0	6780 0	1173 6	3352	664	60176	2996 8	13592	47928	234 04 (48 440)
total fMRI volume (post-NICP)	7553 6	1567 44	2829 6	3040	9640	NA	52160	5048	944	21632	216 32 (59 804)
lateralization index (pre-NICP)	60	82	55	70	81	100	24	70	100	68	70 (27)
lateralization index (post-NICP)	65	55	57	36	94	NA	10	92	100	87	65 (48)
relevance index (pre-NICP)	72	61	48	9	81	73	4	43	49	46	49 (29)
relevance index (post-NICP)	39	82	55	19	94	NA	4	52	100	79	55 (59)
**Analysis by distances**											
distance main cluster-target (pre-NICP)	24	NA	20	10	15	50	59	1	61	83	24 (48)
distance main cluster-target (post-NICP)	20	NA	20	68	11	NA	79	58	60	69	59 (49)
distance main cluster-tumor (pre-NICP)	29	84	38	31	46	28	78	38	64	39	39 (33)
distance main cluster-tumor (post-NICP)	31	85	38	47	43	NA	69	75	68	27	47 (38)

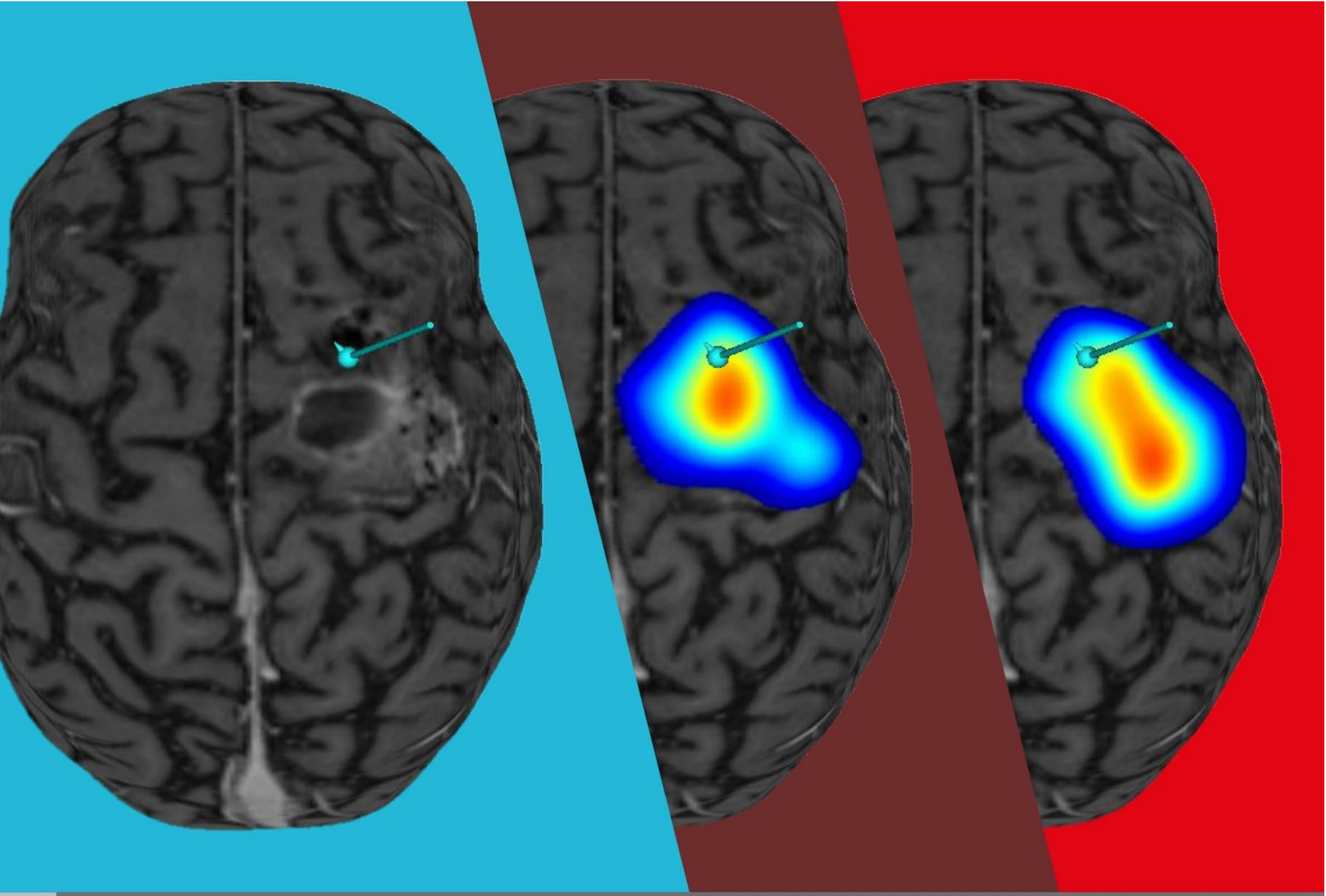

Prehabilitation in neuro-oncology

Step-by-step

A practical manual to understand and apply the research protocol to clinical practice

By Leonardo Boccuni, Guttmann Institute, September 2023.

Prehabilita
Institut Guttmann | Fundació Joan Ribas Araquistain

How to use this manual.

This manual is intended for researchers and clinicians interested in understanding and replicating the protocol developed for the research project PREHABILITA.

The main goal of the intervention is to optimize neurosurgical outcomes in patients with brain tumours, by leveraging neuroplastic changes in a way that allows maximal tumour resection with minimal risk of functional sequelae.

A considerable effort has been made to ensure transparency and reproducibility of the methodology and the results, by registering the trial online (ClinicalTrial.gov ID: NCT05844605) and by publishing the research protocol*.

This manual is a complementary source of information, where readers may find more detailed description of all the procedures that were put into place to manage the intervention. It is intended as a practical, straightforward, and step-by-step manual to those already familiar with the concept of prehabilitation in neuro-oncology. Therefore, before reading this manual, we strongly encourage to read the published protocol to understand the overall rationale of the intervention.

*Boccuni, L., Abellaneda-Pérez, K., Martín-Fernández, J., Leno-Colorado, D., Roca-Ventura, A., Prats Bisbe, A., Buloz-Osorio, E. A., Bartrés-Faz, D., Bargalló, N., Cabello-Toscano, M., Pariente, J. C., Muñoz-Moreno, E., Trompetto, C., Marinelli, L., Villalba-Martinez, G., Duffau, H., Pascual-Leone, Á., & Tormos Muñoz, J. M. (2023). Neuromodulation-induced prehabilitation to leverage neuroplasticity before brain tumor surgery: a single-cohort feasibility trial protocol. *Frontiers in neurology*, *14*, 1243857. https://doi.org/10.3389/fneur.2023.1243857

Acknowledgments.

I would like to thank the Fundació Joan Ribas Araquistain for funding the present research project, and the Institut Guttmann for giving me the possibility of developing the project for my doctoral thesis in Neuroscience.

I would also like to thank my promotors for the doctoral thesis Prof. Tormos Muñoz, Dr. Abellaneda Perez and Prof. Trompetto, for their guidance and valuable mentorship.

A great thank to the multidisciplinary team of physiotherapists, neuropsychologists, neuroradiologists, neurologists, and neurosurgeons that made this project possible.

Finally, I would like to thank from the bottom of my heart the patients that believed in this project and accepted the challenge of undertaking such intensive training before neurosurgery. This manual and all the results are dedicated to them, and to those that are still fighting against cancer.

15th February 2024.

Leonardo Boccuni

Summary (following study timeline).

1. Clinical assessment.

1.1. Clinical assessment – motor function, independency, and quality of life.

1.1.1. Data management – RedCap.

1.1.2. Data management – Teams.

1.1.3. How to structure a motor function evaluation session.

1.2. Clinical assessment – neuropsychology.

2. TMS mapping.

2.1. How to create a Brainsight project.

2.2. How to create a LabChart EMG data acquisition file.

2 Language mapping with Matlab.

2.4. Schematics of TMS lab.

2.5. Stepwise approach for motor and language mapping.

2.6. Differences between the first and subsequent TMS mapping sessions.

2.7. TMS motor mapping analysis.

3. Neuroimaging: main data of interest.

4. Treatment.

4.1. Decision making process for treatment planning.

4.2. Examples of treatments applied so far.

Appendix

1. Clinical and neurophysiology assessment.

Each assessment timepoint is composed of three main branches: MRI, clinical, and TMS. As MRI acquisition and processing is carried out externally (Hospital Clinic), we will focus on clinical and TMS assessment. Furthermore, given that the rationale of the whole intervention has been already reported in the study protocol, in this manual we will deep dive into the practical steps to generate files, manage data, process, and report the outcomes.

1.1. Clinical assessment – motor function, independency, and quality of life.

A physiotherapist manages this part of the assessment. Tools required are as follows (Figure 1):

- A laptop, to note data on RedCap, and to perform the Reaction Time Task.
- Chronometer (within the smartphone).
- Nine-Hole Peg Test equipment.
- Hand dynamometer.
- Neurological hammer, plastic card, pen, can, tennis ball (for the Fugl-Meyer assessment).
- Meter (for the Forward Reach test within the Brunel Balance Assessment).
- Printed copies of the EORTC C-30, BN20, and FA12 (Quality of life questionnaires).
- Printed copy of the PATSAT (patient's satisfaction of the treatment received, only for assessment performed at the end of prehabilitation).

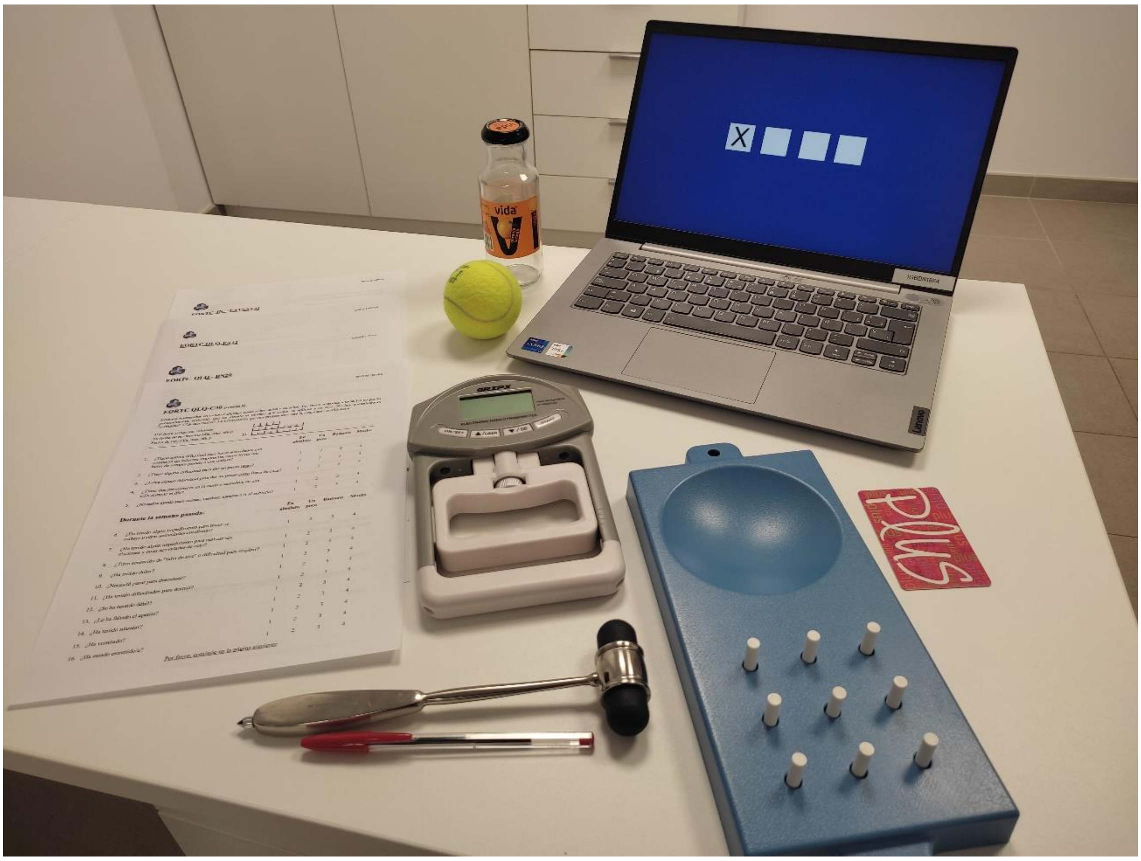

Figure 1. Equipment for the clinical assessment of motor function, independency, and QoL.

To perform the reaction task, do the following steps:

1. Go to https://datashare.ed.ac.uk/handle/10283/2085

2. Click on ‘Download all files’.

3. Open the folders until you reach the file ‘DLRT.exe’ (Figure 2).

4. Double click on the file and fill in the template as depicted in Figure 3.

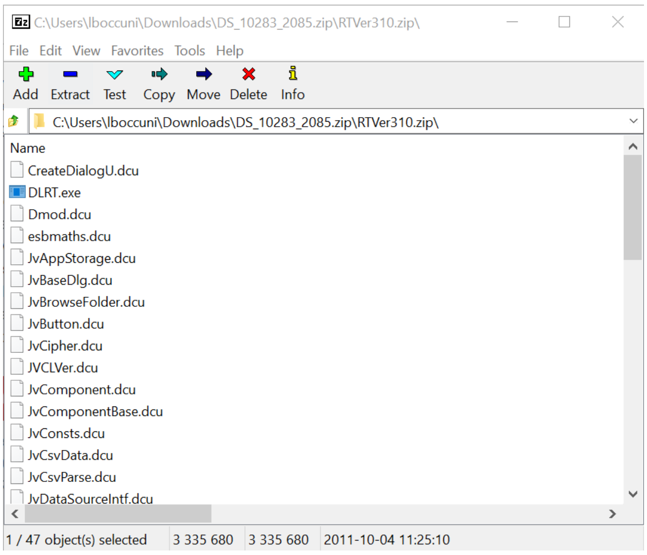

Figure 2. DLRT.exe location

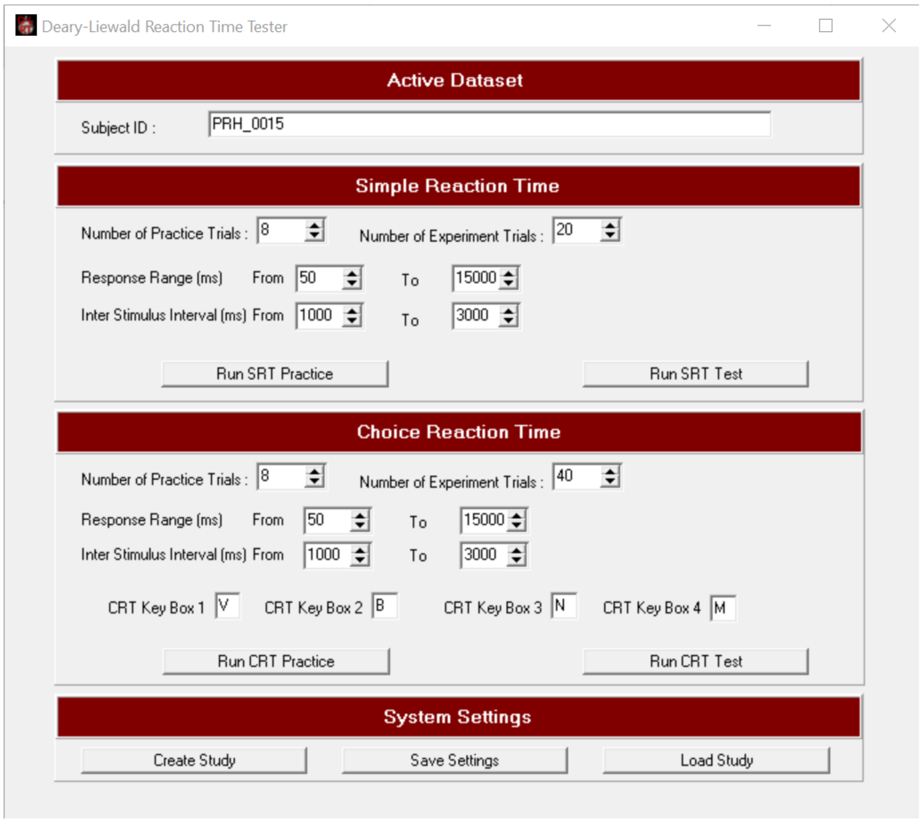

Figure 3. The template for reaction time task.

5. Create two empty folders on the desktop, called 'PRH_0015_T1_RT_aff' and 'PRH_0015_T1_RT_unaff'; the name refers to the patients code, the timing of the assessment (T1, T4, T6), the assessment (RT=reaction task), and whether the hand assessed is on the affected (aff) or unaffected (unaff) side.

6. On the template of the reaction task, when creating a study you have to select the folder related to the hand you are going to assess.

7. When you select the folder, the message 'New Dataset Created and Activated!' should appear. This means that the results of the trials performed will be saved to this folder, as .csv files.

8. Start the assessment with the patient, by clicking in order 'Run SRT practice', 'Run SRT test', 'Run CRT practice', 'Run CRT Test'.

9. For simple reaction task, instruct patient to press the space bar with the hand under assessment, as soon as the letter 'X' appears in the white box at the centre of the screen. Highlight the importance of being as fast as possible because this test is going to measure performance in milliseconds.

10. For choice reaction task, instruct patient to place the fingers over the keys 'V', 'B', 'N', 'M', with the exception of the thumb. This time the patient has to press the key corresponding to the 'X' that appears randomly in one of the four white boxes on screen. Performance must be as fast as possible, yet as accurate as possible (trying not to make errors). Tell patient that whenever an error occurs, the test will continue without any interruption, but the error has been recorded.

1.1.1. Data management – RedCap.

We strived to record all data directly on RedCap, to avoid printing papers and creating excel files. Therefore, the whole data collection for clinical assessment is managed with RedCap.

To use RedCap, login and enter the Project 'Prehabilita'. Then click on the link 'Add / Edit Records' and create or select the patient that you are going to assess.

Here are the list of assessments that should be performed at each timepoint (Figure 4):

- Eortc
- Fugl Meyer Lower Extremity
- Brunel Balance Assessment
- Six minute walk test
- Fugl Meyer Upper Extremity
- 9 Hole Peg Test
- Reaction Time Task
- Hand Dynamometer
- Neurology Assessment in Neuro-oncology
- Karnofsky Performance Status

Some notes:

- Eortc: give the hard copy of the QoL questionnaires to the patient, asking to fill them at home, or the same day during a break in between assessments, and bring them back to you. Then copy the questionnaires on RedCap when the patient is not present; this way,

you save a lot of time during the initial evaluation, by just handing questionnaires to the patient and moving on to the next assessment.

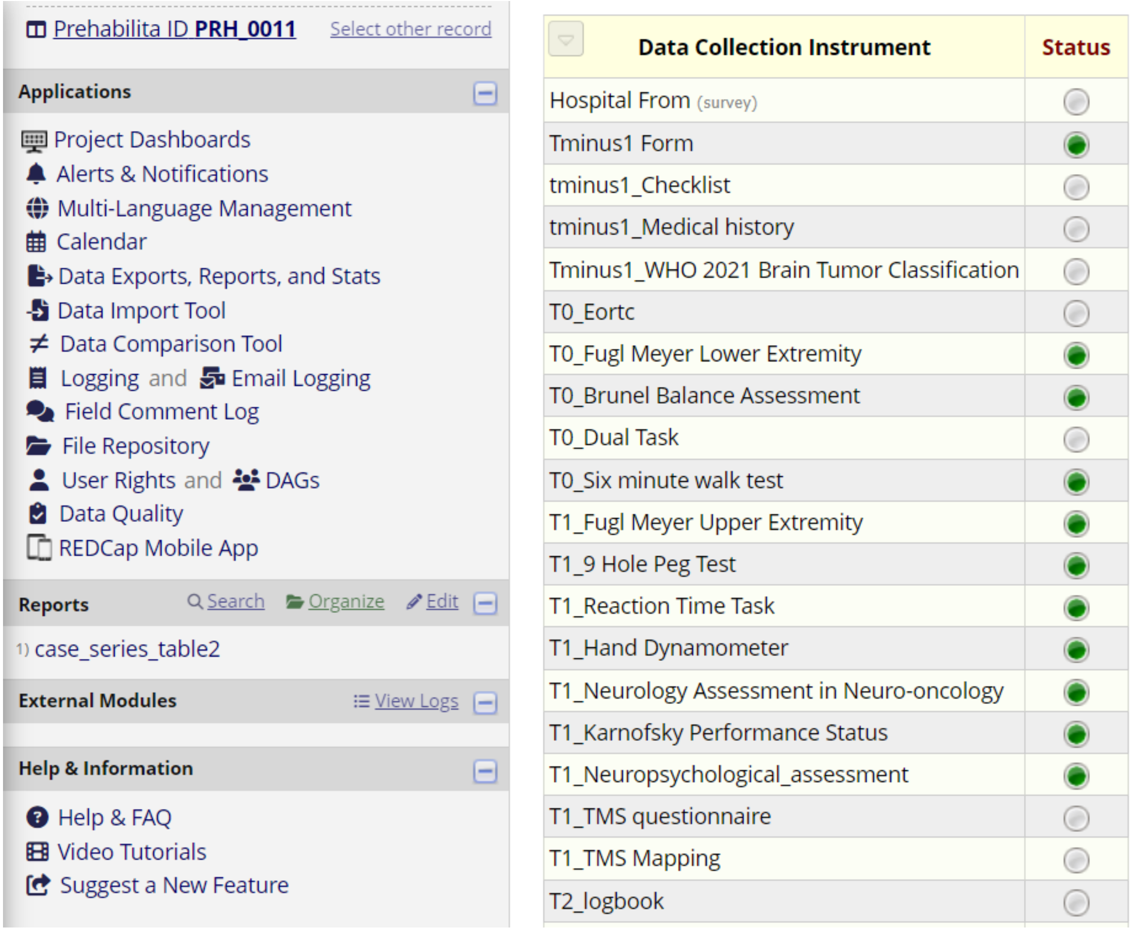

Figure 4. RedCap Prehabilita project.

- Fugl Meyer Lower Extremity: generally, this test is very easy for patients with no lower limb impairment. If this is the case, consider strategies to save time and move on.
- Brunel Balance Assessment: also in this case, generally patients included in the study scores easily the maximum (12 points). According to the rules of the test, you may directly test the last and most difficult trial; if the patient performs it correctly, you can directly score a 12; however, always perform and report the performance of the Forward Reach Test (sitting and standing), as this is a sensitive measure of balance performance.
- Reaction time task: here you report the median of the performance for simple and choice reaction task, for the affected and unaffected hand. To find this value, open the .csv files contained in the folders where you saved the reaction time task. In each folder (one for the affected, one for the unaffected hand) there are four .csv files named ‘SRT_Header’, ‘SRT_Details’, ‘CRT_Header’, ‘CRT_Details’. To find the median, open the headers for SRT and CRT.
- Neurology Assessment in Neuro-oncology and Karnofsky Performance Status: these are two questionnaires of general neurological symptoms, and level of independency. You may fill them without the patient being present, but I often prefer to involve the patient in the selection of each item, and use them as entry assessments to have an initial idea and conduct a sort of structured initial evaluation.

1.1.2. Data management – Teams.

Over the course of the study, a lot of files are generated for each patient. The logic for proper data management on Teams has been to create a folder for each patient, structured as follows:

1st order folder	2nd order folders	3rd order folders	4th order folders
PRH_0015	T1	Clinica	Dual task
			Reaction task
		Mri	Structural mri
			Functional mri
		TMS	1_brainsight
			2_labchart
			3_language
			4_data analysis
	T4	Clinica	Dual task
			Reaction task
		Mri	Structural mri
			Functional mri
		TMS	1_brainsight
			2_labchart
			3_language
			4_data analysis
	T6	Clinica	Dual task
			Reaction task
		Mri	Structural mri
			Functional mri
		TMS	1_brainsight
			2_labchart
			3_language
			4_data analysis

Table 1. Logic of data management on Microsoft Teams. Gray cells indicate folders that are not used because files are saved on another drive (for MRI scans) or the assessment is no longer being used (Dual task).

Each patient's folder is located in 'Projecte Prehabilita' > 'Documents' > 'General' > 'AAA_Prehab Clinical Program' > '1_data collection and analysis'.

To generate an individual patient's folder, select the template 'PRH_000x' and click 'Copy to' the same folder, and rename it with the corresponding patient's code (Figure 5).

In the folder 'Reaction task' you may upload the folders of results generated during the assessment (the .csv files of SRT_Detail, SRT_Header, CRT_Detail, CRT_Header).

Whenever possible, upload files to Teams for the corresponding patient and timeframe. Avoid, for instance, sharing or passing files to other members of the team by email; instead, upload the file on Teams, and then share the link to the file by email.

Finally, consider downloading periodically (one-two months) the whole dataset, and store it on hard disk as back-up copy.

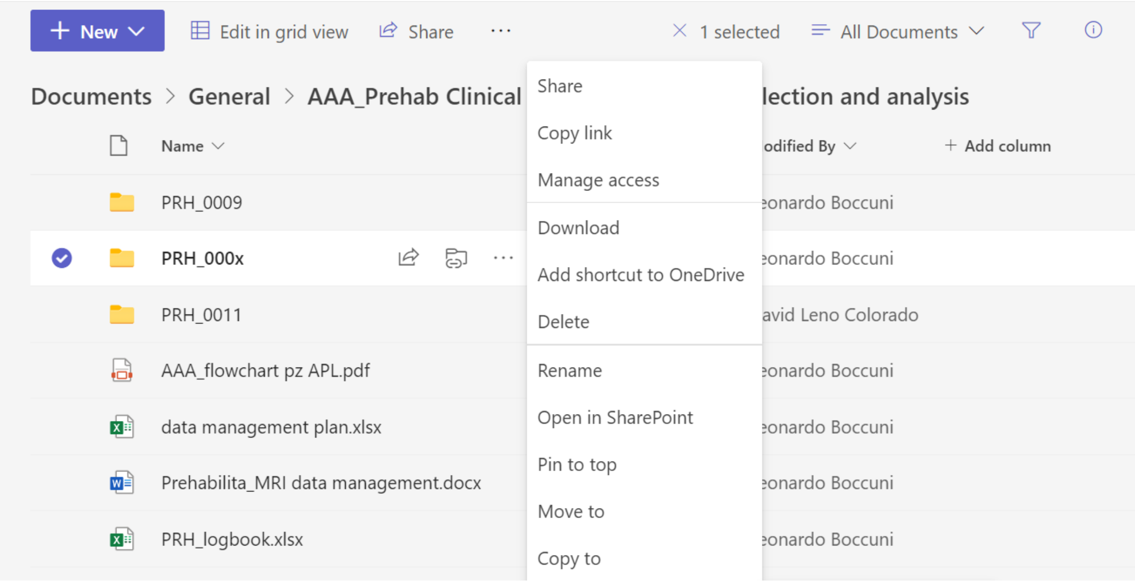

Figure 5. Patient's file template.

1.1.3. How to structure a motor function evaluation session.

Motor evaluation can be time consuming and looks a bit challenging to perform at first, within the same day of neuropsychological assessment and TMS mapping.

The goal is to perform all the assessment in 60-90 minutes. To do so, try these strategies:

- The day before the assessment: collect all the instrumentation needed, print the questionnaires, create patient's file on RedCap and Teams; create two empty folders for Reaction Time Task and save them on the desktop.
- When meeting with the patient: give questionnaires to the patient and ask to fill them later, start with independency and neurological symptoms scale to have a general idea, then all motor assessments for upper limb motor function, then all motor assessments for gait and balance functions.
- Consider adequate time for sensitive assessments measuring units like centimeters, seconds, and kilograms, like reaction time task, 9 hole peg test, dynamometer, forward reach, and 6 minute walk test. Among all measurements, these are the ones that have shown to be more clinically relevant, given the almost complete absence of any neurological deficit preoperatively.

1.2. Clinical assessment – neuropsychology.

The battery of neuropsychological assessment consists of the following tests, adhering to this suggested sequence:

LANGUAGE (Barcelona-Revised Test, TB-R):

a. Spontaneous language (Conversation and narration, Thematic narration, Picture description)

b. Fluency and informative content - Qualitatively assessed based on the information obtained in the spontaneous language subtest.
c. Verbal word repetition
d. Verboverbal naming (Response by naming)
e. Verbal comprehension subtest
f. Reading comprehension (Phrases and texts)

LATERALITY (Edinburgh Handedness Inventory)

ORIENTATION (TB-R):

a. Person
b. Space
c. Time

DIRECT DIGITS - Immediate attention (WAIS-III) - Verbally repeat a series of numerical items in direct order.

REY AUDITORY VERBAL LEARNING TEST (RAVLT) - Immediate recall - Short-term auditory-verbal memory (Rey Auditory Verbal Learning Test) - 15 words are read to the participant, and then they will be asked to repeat as many as they can remember, regardless of the order (note in which order they are repeated). This procedure is repeated a total of 5 times.

TMT A - Selective attention - In this part, the participant is asked to connect a series of numbers in order with a line as quickly as possible and in a single stroke without lifting the hand.

TMT B - Divided attention - In the second part of the TMT, the participant is instructed to connect numbers and letters alternately in order as quickly as possible and in a single stroke without lifting the hand.

NUMBER-CODE - Attention and processing speed (WAIS III) - A response sheet is presented, displaying numbers 1 to 9, each with a corresponding geometric figure. The individual must fill in a grid by drawing the corresponding figure for each number as quickly as possible within a total time of 120 seconds.

VISUAL ATTENTION - Visuospatial skills (TB-R) - With the sheet placed horizontally, the individual is asked to cross out all the triangles they see.

OVERLAPPING IMAGES - Visuoperceptual skills (TB-R) - The individual must identify which figures appear in a series of drawings where objects are superimposed on each other.

PRAXIS (TB-R):

a. Symbolic gesture on command
b. Imitation of postures

BLOCK DESIGN - Executive functions - Planning (WAIS-III) - The individual will be asked to create three-dimensional figures using a set of cubes (9 in total) following a drawing.

REY AUDITORY VERBAL LEARNING TEST (RAVLT) - Delayed recall and Recognition - After the completion of the previous tests and after an elapsed time (approximately 20 minutes), the participant is asked to name all the words they remember from the list. Finally, a list of recognition words is presented (the individual must recognize which words were part of the list and which were not).

REVERSE DIGITS - Executive functions - Working memory (WAIS-III) – Verbally repeat a series of numerical items in inverse order.

LETTERS AND NUMBERS - Executive functions - Working memory (WAIS-III) - A series of items consisting of numbers and letters are presented in a disordered manner, and the patient has to verbally order them so that they first state the numbers in ascending order and then the letters in alphabetical order.

PMR - Executive functions - Letter verbal fluency - The individual is asked to say all the words they can think of that start with "P" in one minute (personal names are not allowed). The same instructions are given for words starting with "M" and "R."

SEMANTIC FLUENCY – Executive functions – semantic verbal fluency – The individual is asked to say all the animals they can think in one minute.

HAYLING TEST - Executive functions - Inhibition capacity:

a. Part A - Complete a series of sentences as quickly as possible with the correct endings.

b. Part B - Complete a series of sentences as quickly as possible with words that do not fit the context of the sentence.

MENTAL CALCULATION - Executive functions - Calculation (TB-R) - A series of mathematical operations is presented, and the participant must verbally solve them as quickly as they can without the use of a paper or pencil.

WISCONSIN CARD SORTING TEST - Executive functions - Mental flexibility, abstract reasoning, categorization - A computerized test in which 4 cards with different shapes, colors, and numbers (categories) are displayed. The individual must match each card from the deck with each of these four cards (without explaining to the individual how to do it). The individual only receives feedback from the computer (Correct or incorrect).

CONNERS CONTINUOUS PERFORMANCE TEST (CPT-3) - Sustained attention - A computerized test in which the individual must press the space bar whenever a letter appears in the center of the screen, except if that letter is X, in which case the key should not be pressed.

ANXIETY AND DEPRESSION SCALE (H.A.D.). Paper or computerized questionnaire

In the event of any language impairment, some additional complementary tests from the TB-R will be administered, as described below:

AUTOMATIC LANGUAGE - MENTAL CONTROL:

a. Forward sequential series
b. Reverse sequential series

VERBAL REPETITION:

a. Syllables
b. Logatoms
c. Phrases

NAMING:

a. Visuo-verbal naming (Images)
b. Verboverbal naming (Naming completion)

VERBAL COMPREHENSION (Complex verbal material)

2. TMS mapping.

TMS mapping includes both motor and language mapping and takes approximately three-four hours to complete. Here we will describe all the practical steps necessary to perform TMS mapping, starting from generating a neuronavigation project on Brainsight.

For general instructions on how to create a Brainsight project, check available online manuals on the company website, or a synthesis in appendix.

For data export, analysis, and reporting, please refer to Chapter 3.

2.1. how to create a Brainsight project for TMS mapping.

The first step is to create a folder (‘PRH_0006’), within the folder ‘PREHABILITA’ on the desktop of the computer in the TMS room. Once we obtain the structural MRI (nifti file) from the radiologists of Hospital Clinic, save a copy of the MRI scan in the folder ‘PRH_0006’, naming the file ‘PRH_0006_T1’).

These initial steps are important because, once you create a Brainsight project, the software will always search for the exact location and the exact name of the MRI scan file. Whenever you edit the name of the file, or move to another folder/computer, the software will be unable to upload the MRI scan in the Brainsight project. So, better to be systematic with folders and file names in advance. Also, save the newly created Brainsight project in the same folder (Figure 6).

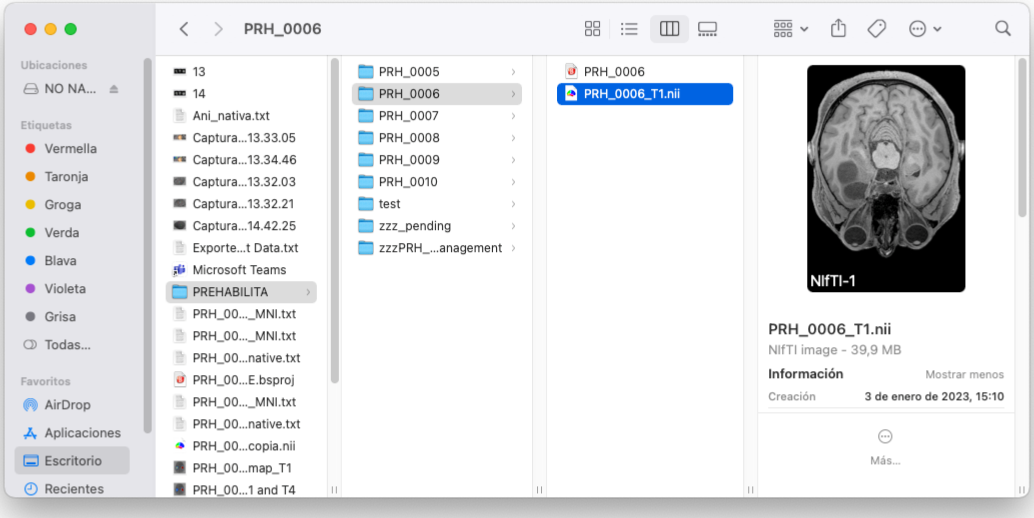

Figure 6. Organization of files for Brainsight project.

Let’s move through each step, as outlined in the Brainsight project:

Atlas spaces. Another issue that may arise when creating the project is the configuration of Atlas Spaces based on the localization of the anterior and posterior commissure (Figure 7). In fact, in case of tumours of moderate size, anatomy may be already altered to the point that these two reference points are no longer visible, or in place (Figure 8). In this case, try to estimate where

anterior and posterior commissure should be located, and run several trial until reaching satisfactory scaling of the brain (Figure 9).

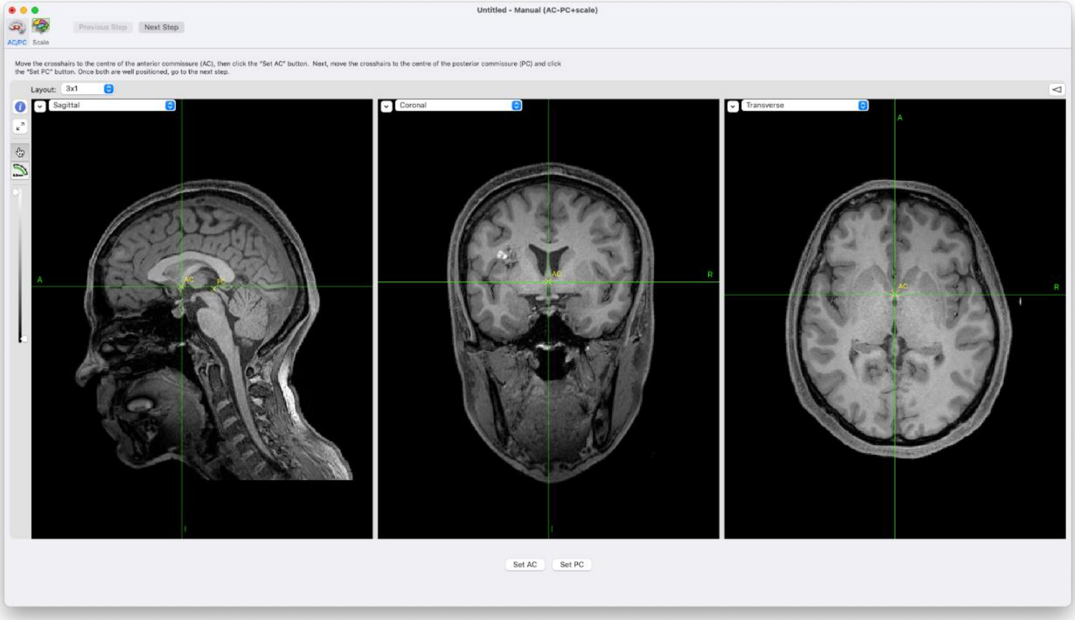

Figure 7. Atlas Spaces manual configuration in a patient without significant neuroanatomical alterations at the level of the anterior and posterior commissure.

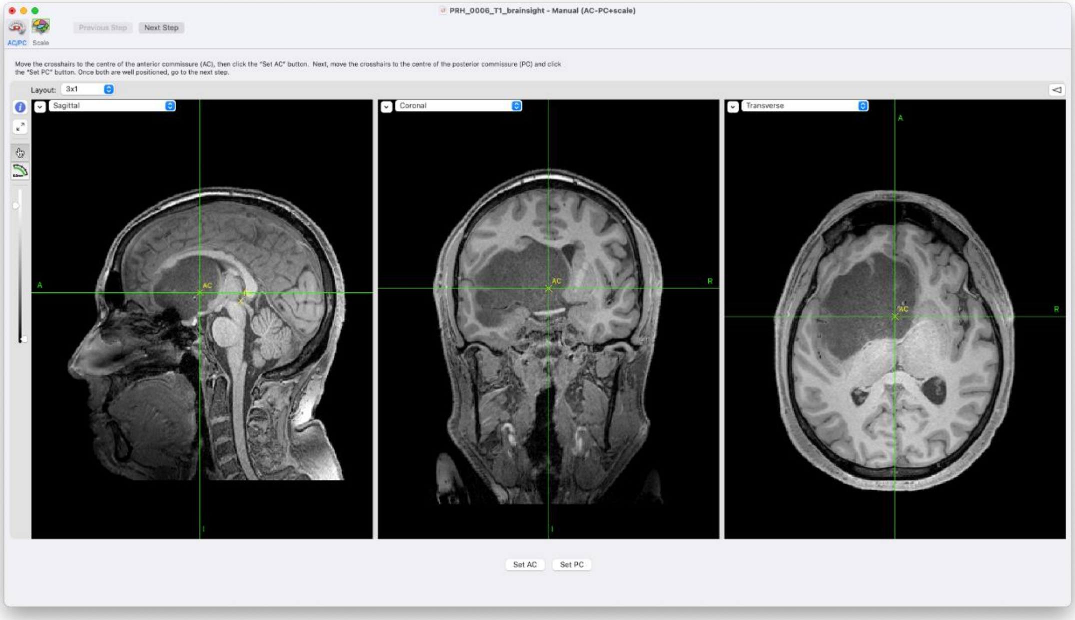

Figure 8. Estimated localization of anterior and posterior commissure in a patient with severe disruption of neuroanatomy.

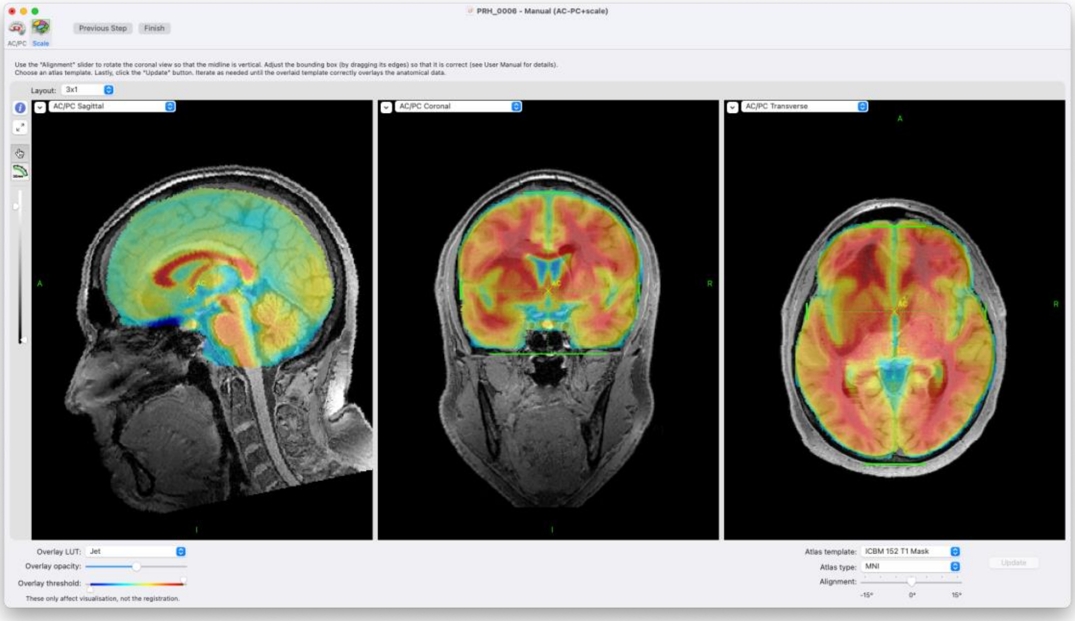

Figure 9. Adequate brain scaling reaching, despite approximate estimation of anterior and posterior commissure localization.

Overlays. This section is optional, but very useful whenever fMRI results are already available. In this section, just upload the fMRI clusters by clicking ‘Add’ and selecting the appropriate nifti file. At this stage you may change the opacity and the Look Up Table (LUT) of preference (Figure 10).

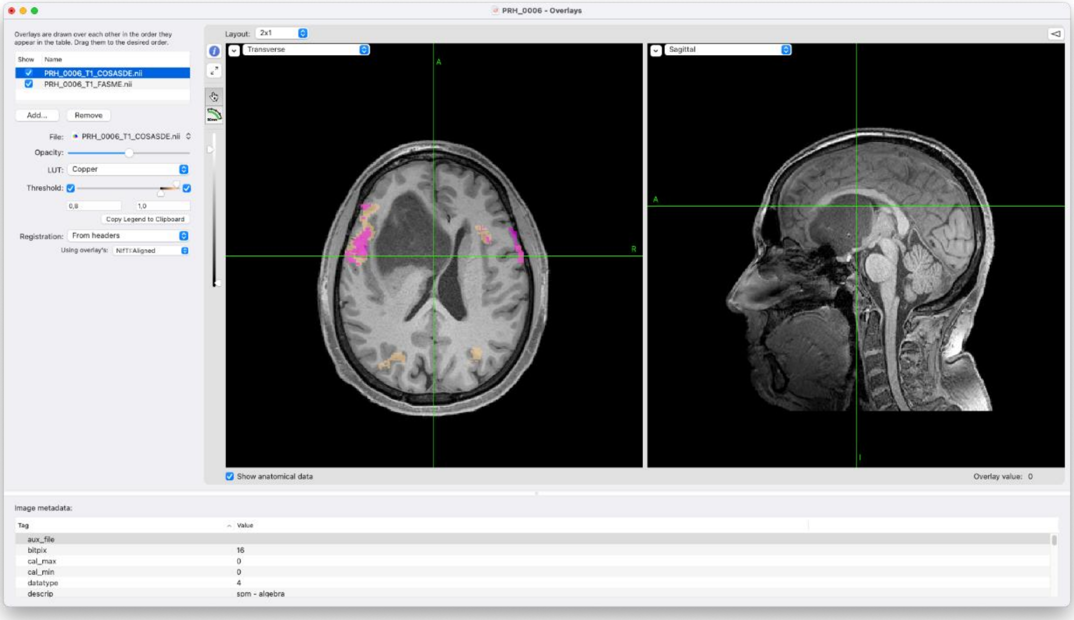

Figure 10. overlay of fMRI clusters for language-related tasks.

Reconstructions. Reconstruct Skin and Curvilinear Brain as usual, and then apply to Curvilinear Brain the appropriate peel depth to have the cortex clearly visible (Figure 11).

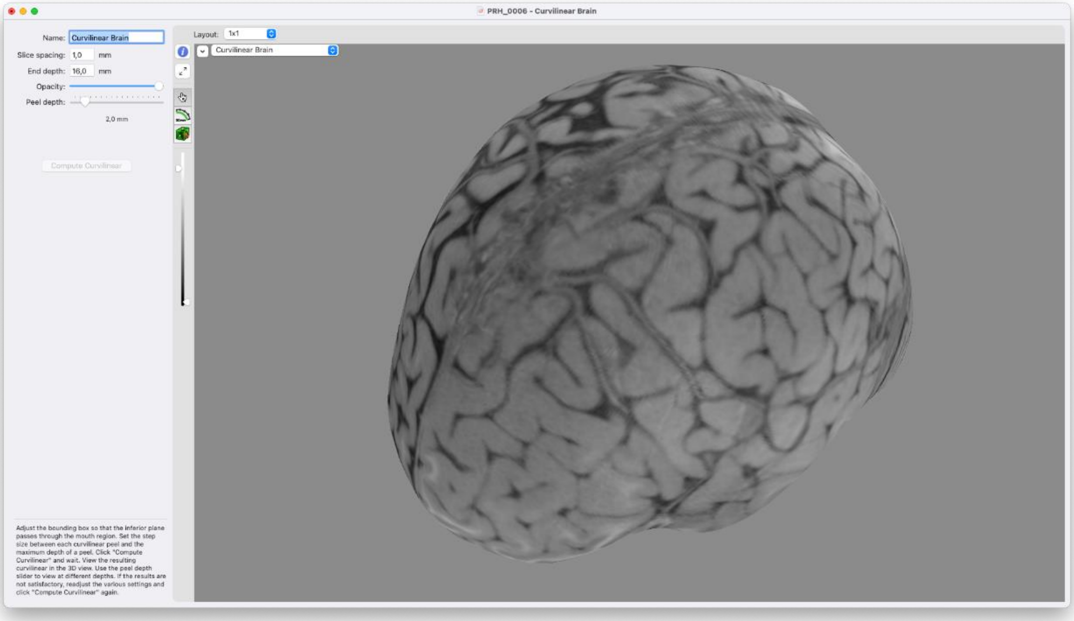

Figure 11. Peel depth set at 2,0 mm to clearly visualize cortical structures.

Landmarks. In my experience, four landmarks are better than three for head reconstruction in subsequent sessions (Figure 12). Sometimes, the tip of nose or the right/left preauricular areas are not available. In these cases, search for alternatives, like the left and right eye cantus.

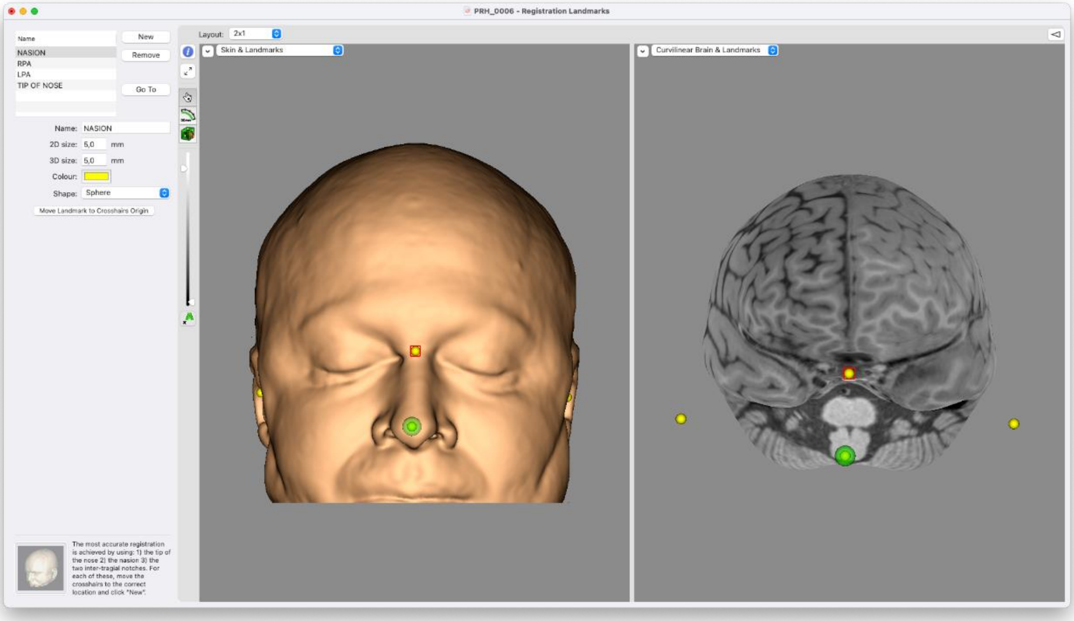

Figure 12. Four skin landmarks at the level of nasion, tip of nose, LPA and RPA.

Targets. This is by far the most laborious part of the Brainsight project (Figure 13).

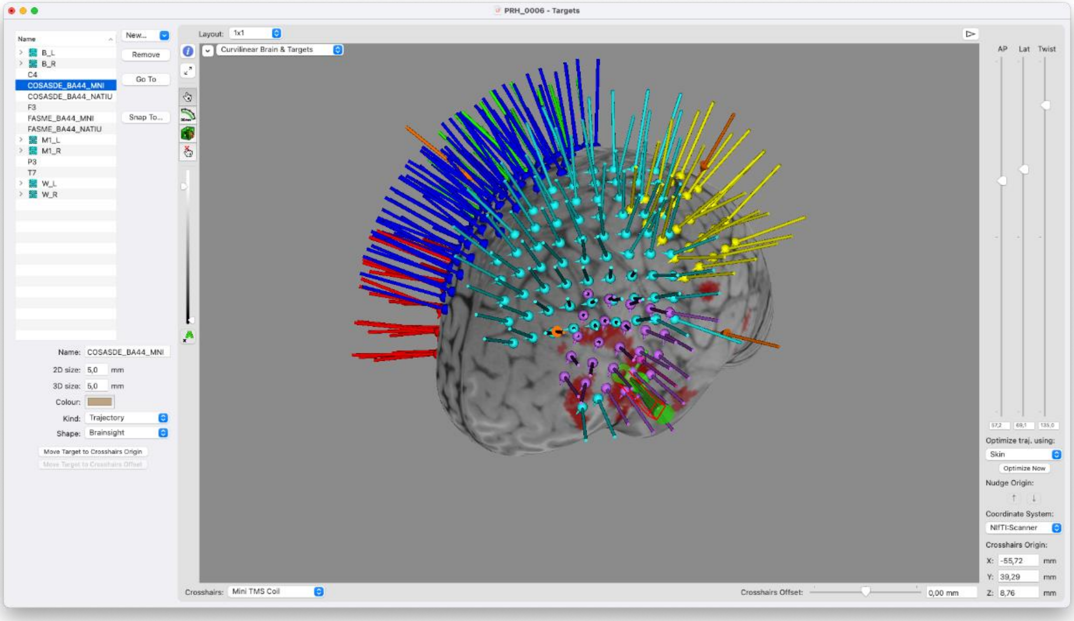

Figure 13. Definition of targets for TMS motor and language mapping.

We create in total six grids, and eventually single targets for specific points of interest. Within each hemisphere, one is for mapping motor evoked potentials of the first dorsal interosseus ('M1'), one for language mapping corresponding to the Broca's Area ('B'), and one for language mapping corresponding to the Wernicke's Area ('W'). Given that each side is identified con _R (right) and _L (left), we should end up with the following grids:

M1_R, M1_L, B_R, B_L, W_R, W_L.

The size of M1 grids are 9 x 9, 10 mm space in between targets. The reason for such enormous grid is that mapping starts from the hotspot, and then progress around until MEPs disappear (0/6 all round the motor map). Therefore, it's useful to create a large grid to be sure of not missing out any potential positive target; those targets out of the borders will simply not be mapped.

The size of B and W grids are 5 x 5, 10 mm space in between targets. Being composed of 25 targets is important, because the Matlab script for language mapping has been developed accordingly, and a mismatch may cause problems. In this case, every target will be mapped 2-3 times, so it's not recommended to create grids larger than that, for timing reasons.

If we have peak fMRI coordinates and wish to use them to create a target on our Brainsight project, we can type manually the coordinates on the bottom right (Figure 13). If coordinates are in MNI, be sure to set in advance the Coordinate System as 'MNI'.

2.2. Motor mapping. How to create a EMG data acquisition file.

The goal of this chapter is to generate a template for EMG data acquisition during TMS motor mapping. The data acquisition and analysis system is PL3508 PowerLab 8/35, the amplifier is FE234 Quad Bio Amp, and the software is Labchart (ADInstruments). The three main elements to define are channel settings, settings for data export, and settings for block registration.

Note: Before launching Labchart, a mandatory step is to first switch the hardware ON. This way, Labchart will recognize and pair to the hardware.

Channel settings. Open a new template, and in the main menu select ‘Set up’ > ‘channel settings’.

On the bottom left of the page, select the number of EMG channels (1 to 8). Each row corresponds to a EMG channel, while each column corresponds to a variable of signal processing.

For the present protocol, settings are as follows (Figure 14):

- Number of channels: 2 (channel 1 for the first dorsal interosseus (FDI), and channel 2 for the abductor pollicis brevis (APB).
- Channel title: ‘Ch1_FDI’ for channel 1, and ‘Ch2_APB’ for channel 2.
- Device input: corresponding channels from the hardware.
- Sample rate: 4k/s.
- Range: 10 mV.
- Input Amplifier: Bio Amplifier.
- Units: mV.
- Color and Style refers to the line of the EMG signal.
- Calculation: Digital Filter (Figure 15).

Once these settings are defined, the main page of the template should appear as Figure 16.

A last set-up is the trigger for EMG registration (Figure 17), which comes from the TMS pulse. On main menu click ‘Set-up’ > ‘External trigger’ and select as trigger type ‘Normal (voltage level)’.

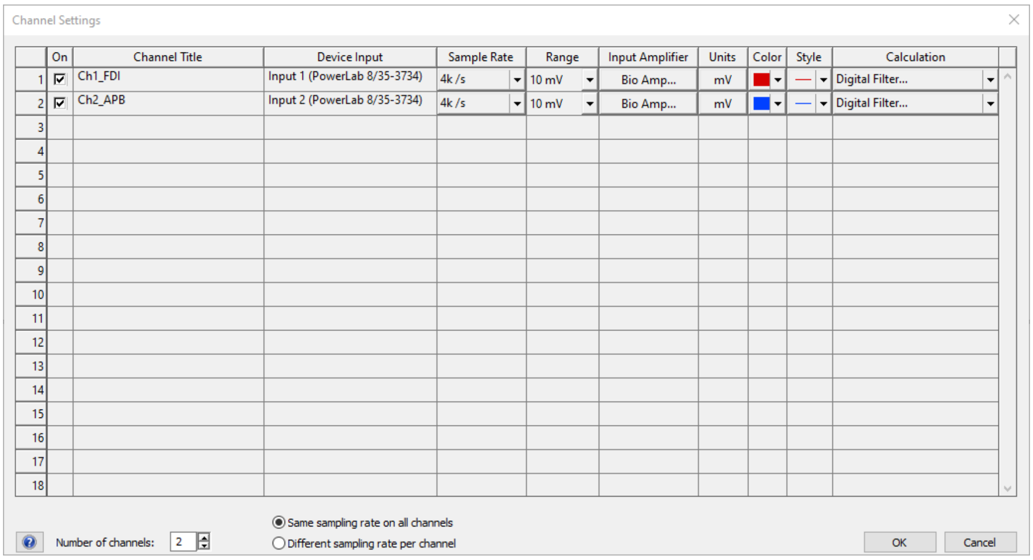

Figure 14. Channel Settings on LabChart.

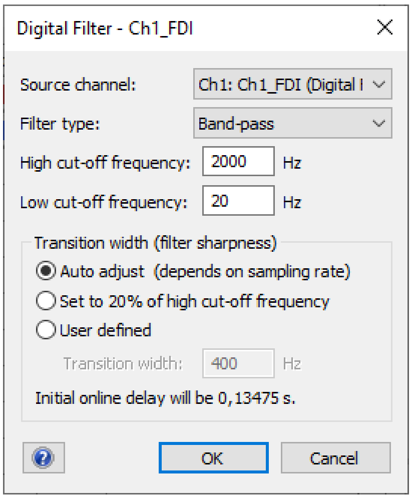

Figure 15. Digital filter, where band-pass filter was selected in the range 20-2000 Hertz.

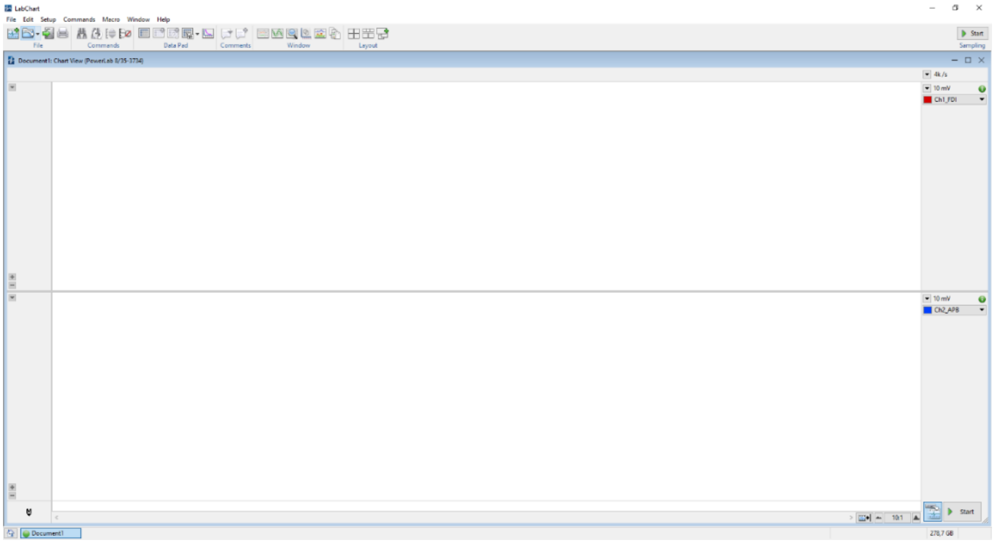

Figure 16. Main page of the template.

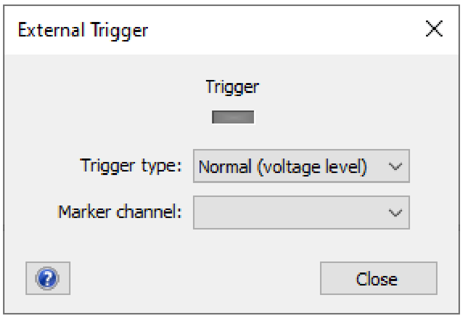

Figure 17. External trigger set-up, to receive the input at each TMS pulse.

Settings for data export. From the main menu, click on 'Data Pad' to open a new window where selected variables will be registered and exported (Figure 18). Each line corresponds to a 'Block' or 'Page'. A page is a section of EMG recording related to a trigger (a TMS pulse).

Columns are named 'A', 'B', 'C'… in alphabetical order. To create a variable of interest, click on the corresponding column to open a menu of variables. In this case, we selected the following:

- Column A (Figure 19): Block start date (day, month, year of recording).
- Column B (Figure 20): Block start time (hour, minute, seconds, milliseconds of recording).
- Column C (Figure 21): Ch1_FDI Full comment text (any comment added to FDI recording).
- Column D (Figure 22): Ch1_FDI Maximum-Minimum (peak-to-peak amplitude for FDI).
- Column E (Figure 23): Ch2_APB Maximum-Minimum (peak-to-peak amplitude for APB).

Settings for block registration. On the main menu, click on 'Window' > 'Scope Pages'. It will open a separate window 'Scope view' (Figure 24).

Click on the bottom 'Sampling' and select the start and stop of the recording at -100 ms and +300 ms, respectively. The time refers to the trigger event (0 ms).

Once all these settings have been defined, save the template. Before each session, open the template and click 'Save as…' to save a separate file to record that specific session, with the same settings of the template, but leaving the template file in the original format.

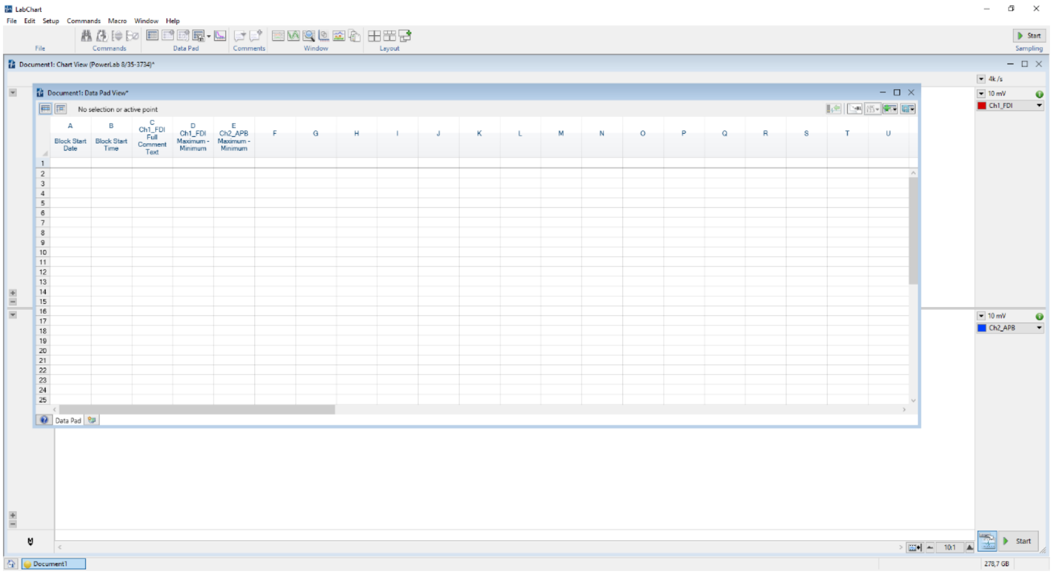

Figure 18. Data Pad window.

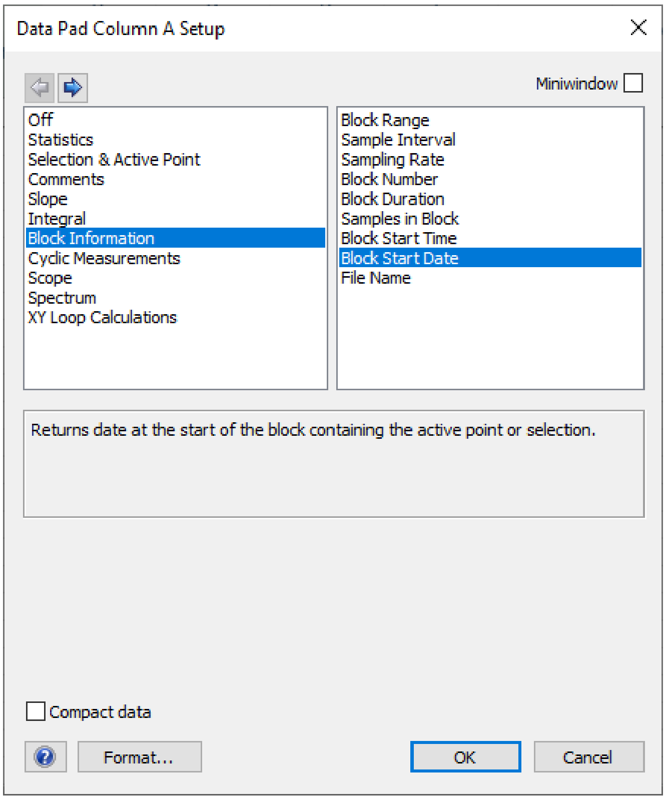

Figure 19. Set-up of column A.

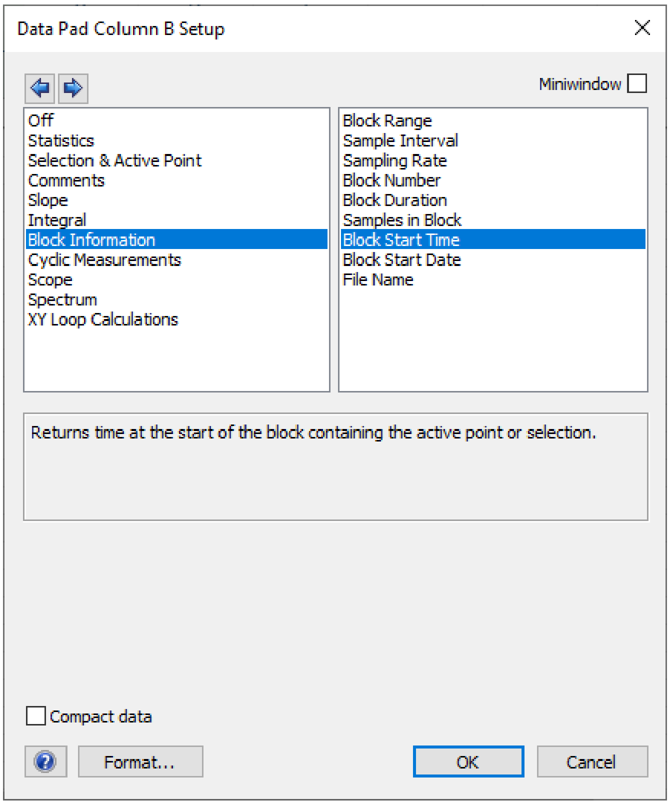

Figure 20. Set-up of column B.

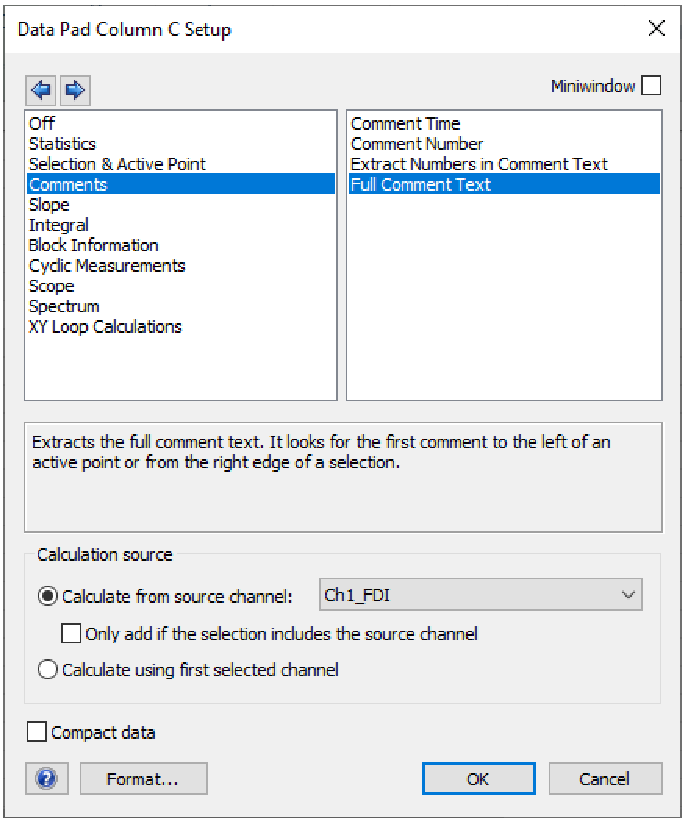

Figure 21. Set-up of Column C.

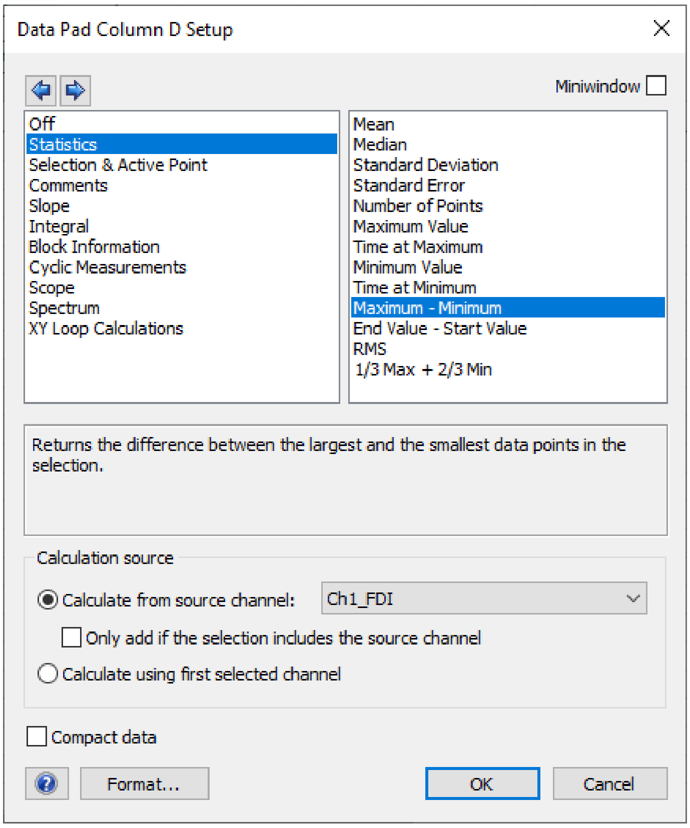

Figure 22. Set-up of Column D.

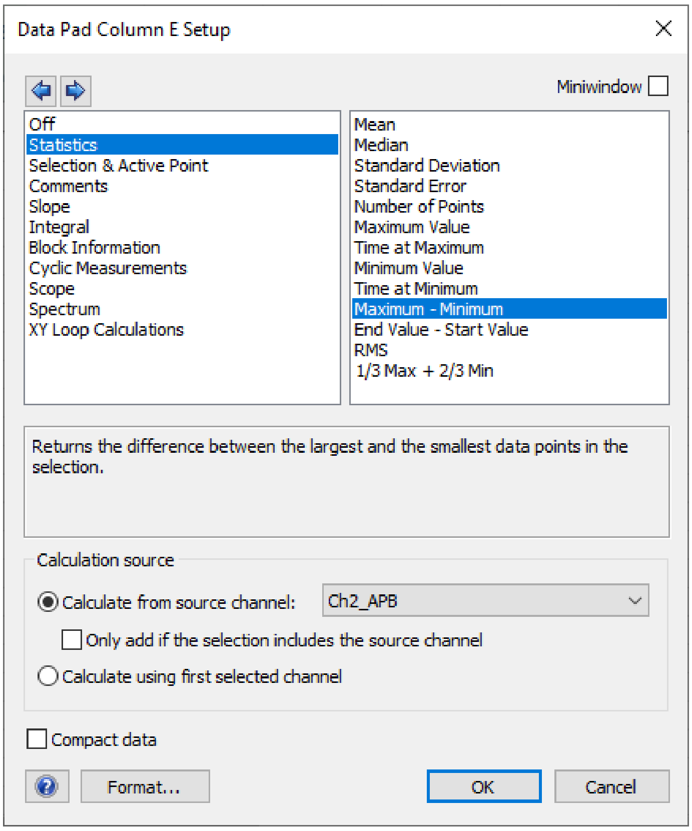

Figure 23. Set-up of Column E.

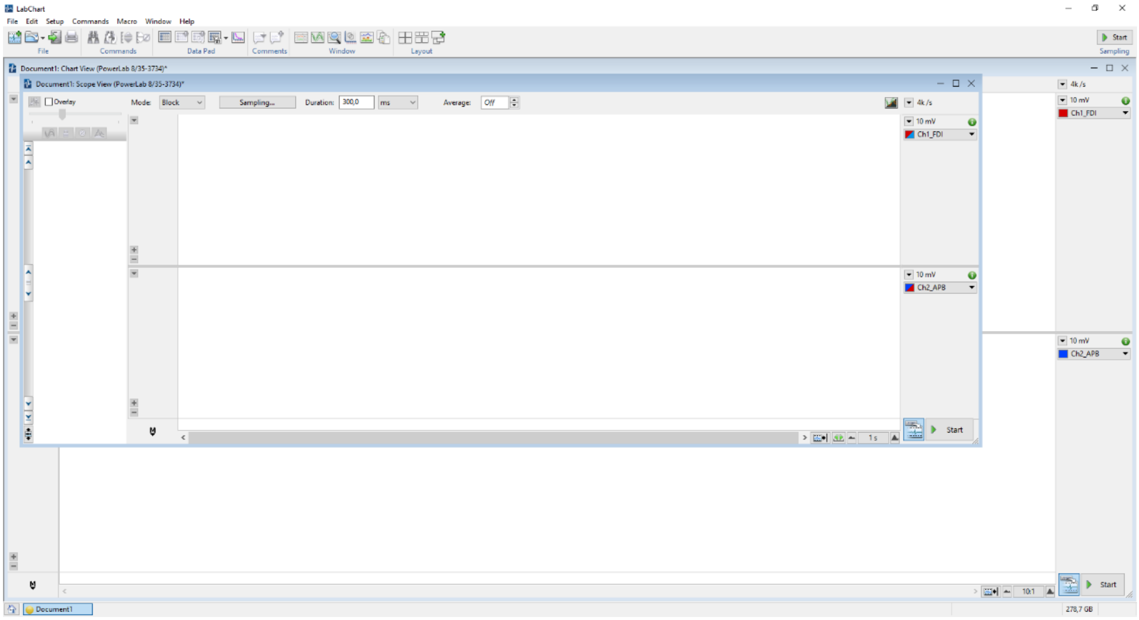

Figure 24. Window for scope view.

2.3. Language mapping with MATLAB.

The objective of language mapping is to identify cortical targets related to language function. The protocol is semi-automated by using an in-house developed Matlab script (Appendix).

Set-up. There are two operators, the first one is a neuropsychologist seated in front of two computers, and the second one behind the patient, holding the TMS coil. The tasks of the neuropsychologist are to select the target on Brainsight (computer on the left side), launch MATLAB script and trigger the TMS stimulation by pressing the letter 't' on the keyboard (computer on the right side), and evaluate patient's verbal response to verify the presence of any speech disturbance. The task of the second operator is to position the TMS coil on the selected target; once the TMS coil is on the target, the neuropsychologist presses the letter 't', check patient's response, and then select the next target on the list.

The patient is seated in front of a screen showing a grey picture with a white cross in the middle, and instructed to watch towards the cross and name out loud anything that will appear on the screen. For instance, if the image of a dog appears on the screen, the patient must say 'dog' as quickly and clear as possible.

Language mapping is always performed after motor mapping, with the following parameters:

- Number of pulses: 5.
- Frequency: 5 Hertz.
- Intensity: 90% RMT.
- Delay between the first pulse and image presentation: 0 ms.
- Duration of image presentation: 500 ms.
- Duration of audio recording of patient's response: 4 seconds.

To recap, when the neuropsychologist presses the letter 't' there are three events starting at the same time: a TMS train of 5 pulses at 5 hertz and 90% RMT, image presentation, and audio recording.

If the patient can name the image correctly, the target is classified as 'negative'.

If the patient presents any difficulty in naming the object correctly, the target is classified as 'positive', and the type of speech disturbance is noted down.

To use the MATLAB script, it is necessary to install Psychtoolbox on the computer, and create folders of images that will be presented to the patient randomly.

2.4. Schematics of TMS lab.

Figure 25 shows a schematic of the TMS lab. Cables were in-house soldered to connectors so that the TMS may receive input from the computer where MATLAB was installed and send a trigger output to both Brainsight and PowerLab.

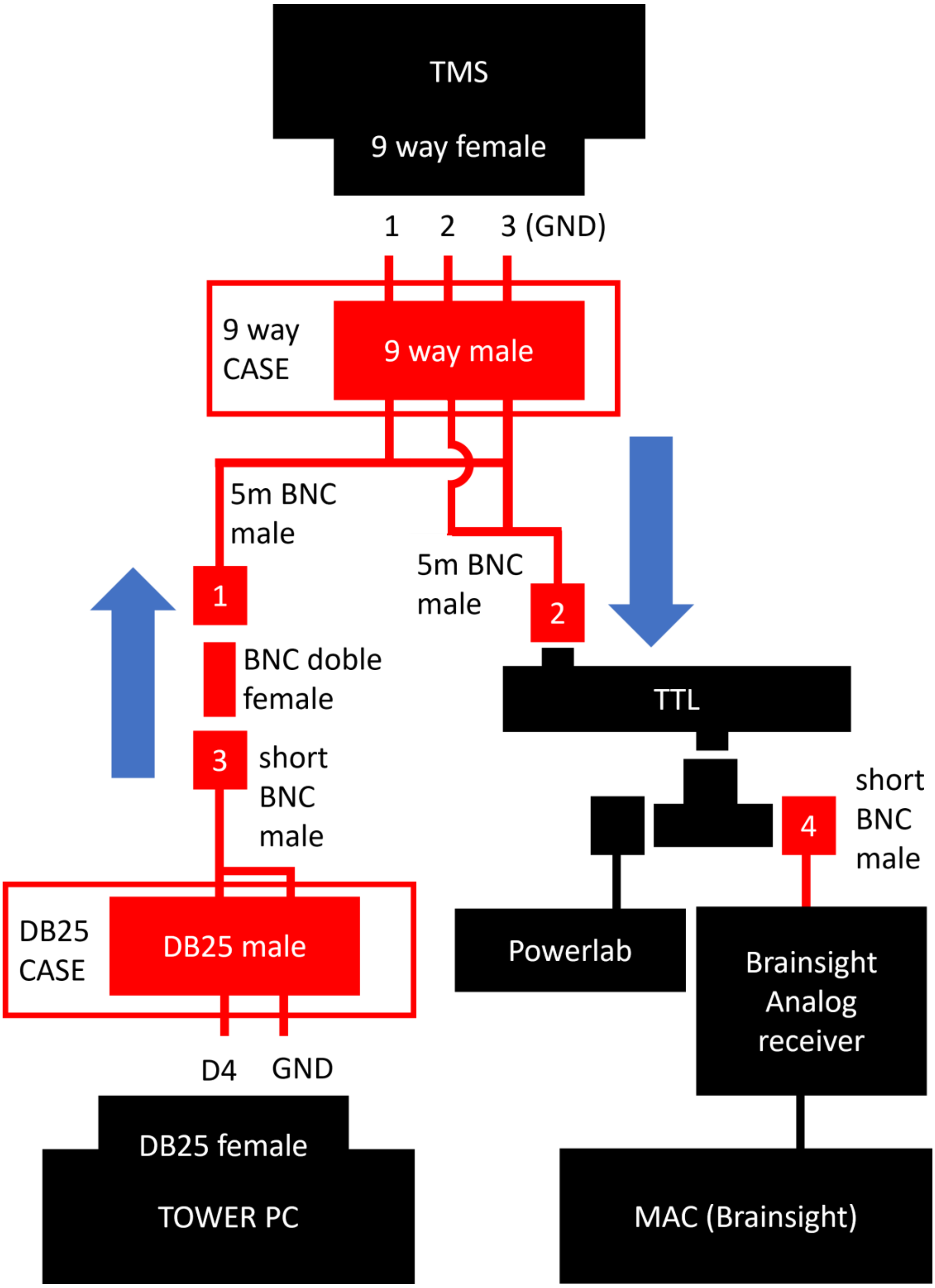

Figure 25. Schematics of the TMS lab.

2.5. Stepwise approach for motor and language mapping.

Here is an example of the typical steps needed to perform a TMS mapping session. The three macro-categories are steps ‘before the patient enter the TMS lab’, ‘when the patient is in the TMS lab’, and ‘after the patient exits the TMS lab’.

Part 1. Before the patient enter the TMS lab.

Step 1. Print the logbook for TMS mapping (Appendix).

Step 2. Launch Brainsight, open patient’s project, and select Session > Online session.

Step 3. Select all the targets for stimulation, which are the grids named M1_L, M1_R, B_L, B_R, W_L, W_R. Each time a grid for language mapping (B…, W…) is selected, click on the button ‘Randomize selected’ to generate a random sequence of targets within the grid, which is important during language mapping to avoid order effects.

Step 4. On the page ‘IOBox’, thick the flag for TTL and write 2000 milliseconds as dead time. This selection ensures that a sample is recorded each time that a TMS pulse is delivered, BUT there is a temporal window of 2 seconds after the pulse during which there is no recording of samples. This is important both during motor mapping, in case there is a double stimulation by accident, and during language mapping, where five pulses at five hertz are delivered on the same target, but only the first pulse is detected to record a sample (recoding a sample for each of the remaining four pulses would be unnecessary and redundant).

Step 5. On the page ‘Polaris’ verify the connection with the Polaris (area of the camera depicted in green). If the Polaris is not connected, click on ‘Reset Polaris’, check cable connection, and eventually click on ‘Window’ > ‘Polaris configuration’ to select the Polaris.

Step 6. Turn the TMS ON and on the ‘Trigger Menu’ select the appropriate polarity of the output to Brainsight and Labchart. Note: to avoid mistakes, you may select ‘Rising edge’ for both ‘Polarity input’ and 'Polarity output'. Polarity is set by default on ‘Falling edge’ (Figure 26).

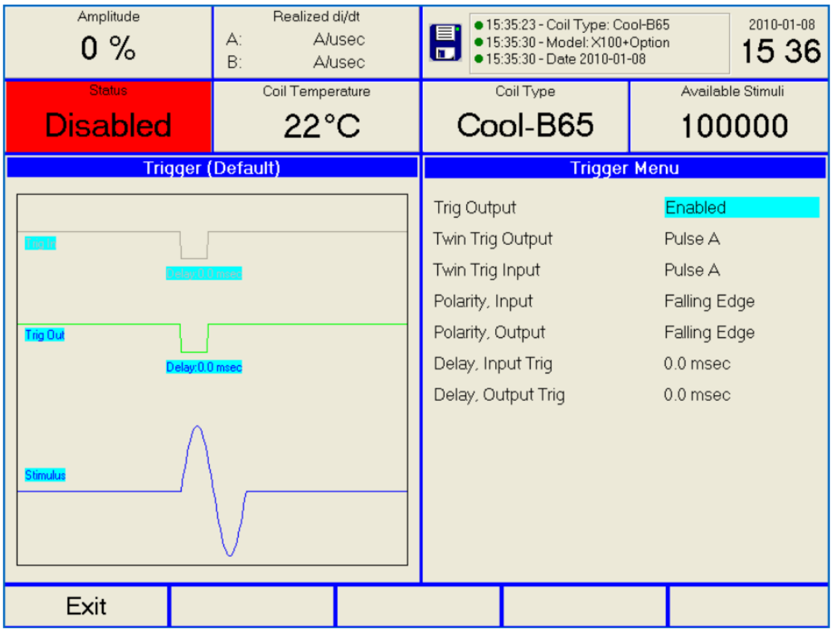

Figure 26. Trigger Menu on MagVenture.

Step 7. Leave the MagVenture on the 'Timing Menu' page (Figure 27) for the whole experiment, to ensure a correct communication between Magventure and Matlab.

Step 8. On the other computer launch the Matlab script and verify the correct functioning of the programme.

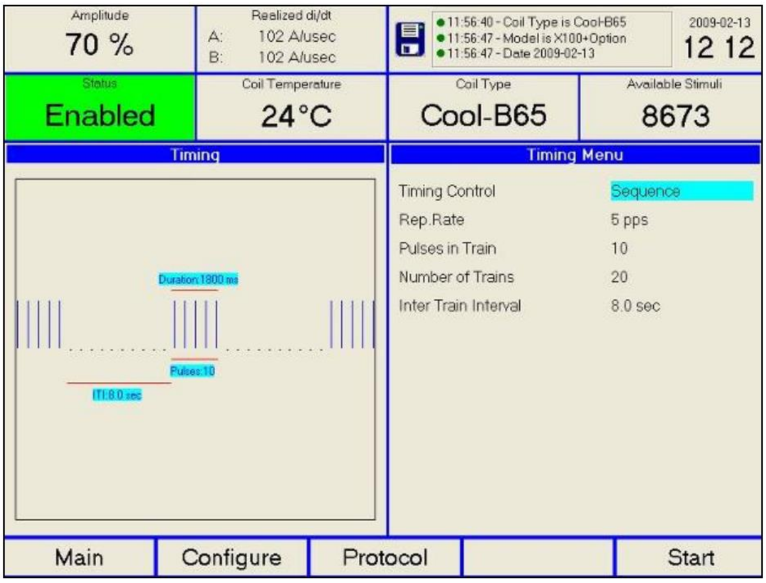

Figure 27. Timing Menu on MagVenture.

Step 9. Turn Powerlab ON and launch Labchart, open the template and save a new file for the TMS motor mapping session. You may name the file 'PRH_000x_Tx_TMSmap', where the first field indicates patient's code, and the second field the time of the study (T1, T2, T3…).

Step 10. Prepare all the material needed: subject tracker, pointer, EMG electrodes, ethyl alcohol and cotton swabs for skin preparation, pillow to be placed underneath the hand and forearm at rest during TMS motor mapping. Recommended: re-calibration of the TMS coil.

Part 2. When the patient is in the TMS lab.

Step 1. Seat the patient comfortably, while ensuring that the trunk is held upward. Explain the general purpose of the session and all the steps that are performed along the session.

Step 2. Place the head tracker and go through the steps 'Registration' and 'Validation' on Brainsight.

Step 3. After proper skin preparation, place the EMG electrodes on the unaffected hand ipsilateral to the tumor (Figure 28). It is recommended to start TMS motor mapping on the unaffected side, to verify patient's RMT and MEPs in the 'normal' hemisphere.

Step 4. Click 'Start' on LabChart to check the quality of the signal. You may ask the patient to open the hand and relax, as additional check. Don't ask the patient to make a fist because it may detach the electrodes. Open Scope View and the window of FDI peak-to-peak, to check at the same time the continuous EMG signal and the MEPs (Figure 29).

Step 5. Set TMS intensity at 35% and press two-three times (with 3-5 seconds interval in between) the button on the TMS handle with the coil held in the air, to check proper sample registration (on the page ‘Perform’ of Brainsight) and page registration (on the page 'Scope Pages’ of labChart).

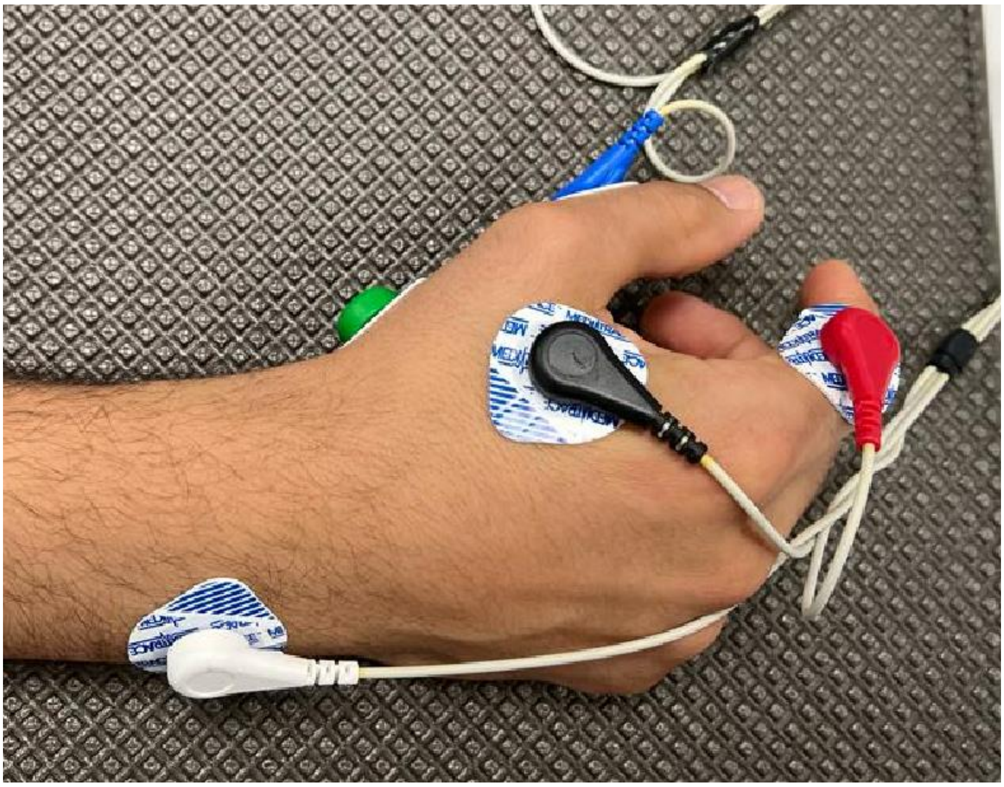

Figure 28. Belly-tendon montage for FDI and APB.

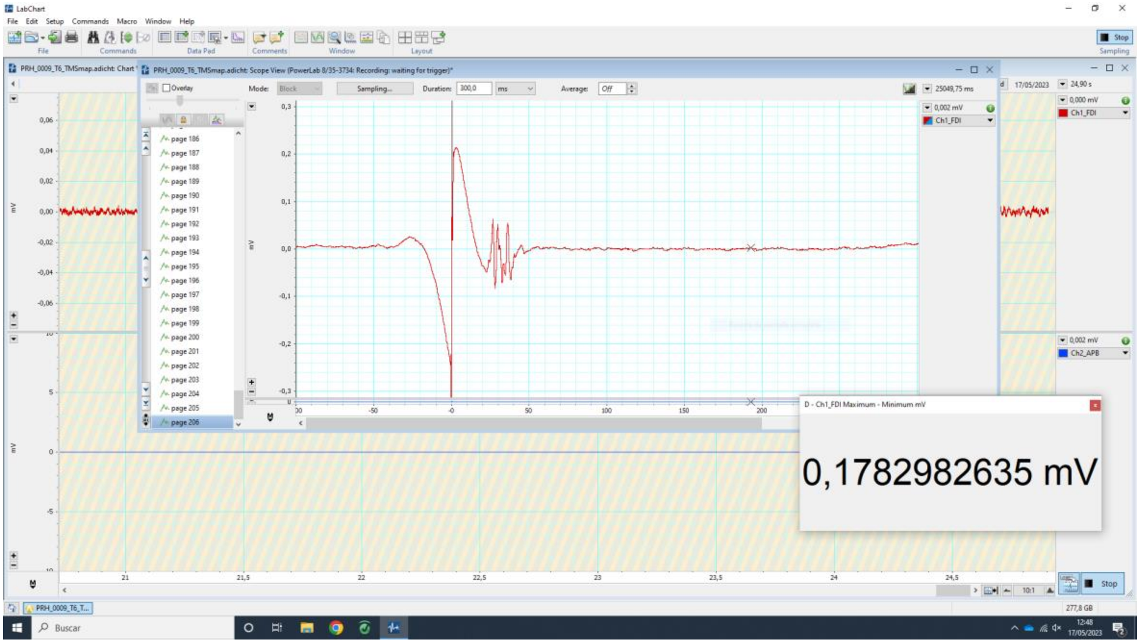

Figure 29. LabChart configuration during TMS motor mapping.

Step 6. Select the target 4,4 in the grid for the motor area of the unaffected hemisphere, which should correspond to the anatomical location of the primary motor area of the hand (hand knob). From 35% rise TMS intensity in steps of 5% until you detect a stable MEP (peak to peak amplitude of about 500 microVolts). If at moderate intensities (60-70%) no MEPs are elicited, select other targets nearby.

Step 7. Once stable MEPs are detected, calculate the average peak-to-peak of five pulses (five seconds interval in between) on a certain target, and repeat the process on the targets nearby (keeping the stimulation intensity constant). The target that shows the largest peak-to-peak response is the M1 hotspot. Note target name on the logbook.

Step 8. Once the hotspot has been identified, lower down the TMS intensity until we have negative MEPs. Positive MEPs are defined if at least 5 pulses out of 10 elicited an EMG response with peak-to-peak greater than 50 microVolts. RMT is defined as the intensity at which we have negative MEPs, + 1%. Note RMT on the logbook, together with 120% RMT (TMS intensity for motor mapping) and 90% RMT (for language mapping).

Note for step 7-8. In case of necessity to reduce the time to perform the whole session, consider this alternative methodology: identify a target where stable MEPs are elicited, then lower down the intensity until we have negative MEPs (3 out of 6 instead of 5 out of 10), then with this intensity check nearby targets to see if positive MEPs could be elicited. In case of positive MEPs on another target, lower further down the intensity until we have negative MEPs and repeat the process. The hotspot is the target where we obtained positive MEPs at the lowest intensity, and the RMT is the corresponding intensity.

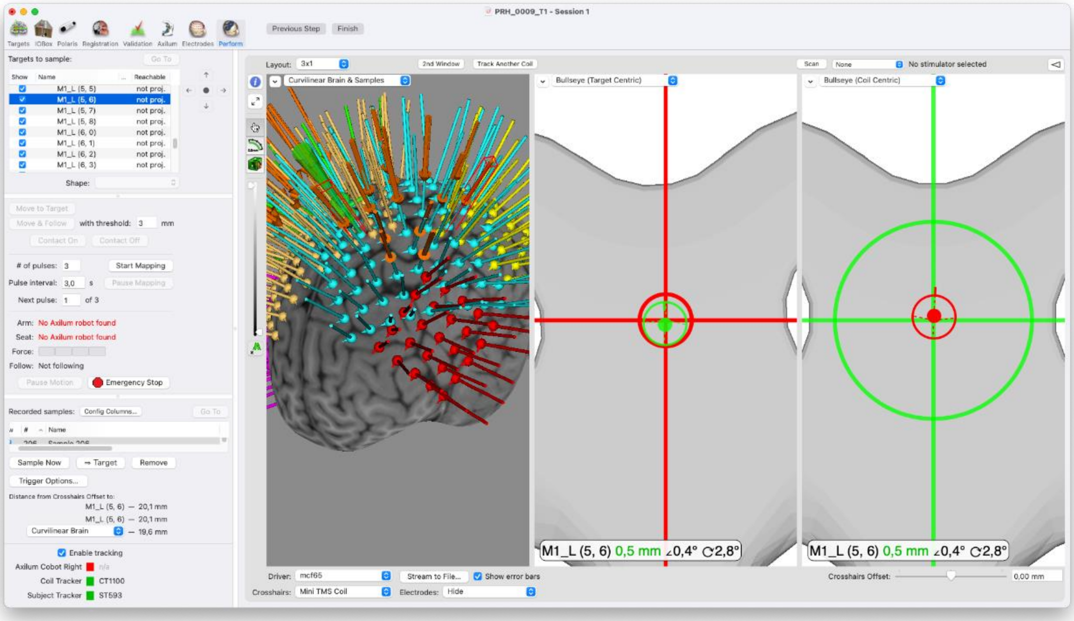

Figure 30. Brainsight window during TMS motor mapping.

Step 9. Perform TMS motor mapping, with the following parameters: intensity of 120% RMT, 5 pulses delivered on each target with at least five seconds of interval in between. Motor mapping starts from the hotspot and then move to adjacent targets randomly, to avoid order effects.

If at least one MEP out of five is positive, the target is classified as MEP+ and adjacent targets are mapped. If no MEP out of five are elicited, the target is classified as MEP- and adjacent targets are not mapped.

During motor mapping it is necessary to take note on the logbook of the MEP+ (X) and MEP- (0) (Figure 31). Any other notes related to motor mapping should be written directly on Labchart, by inserting a comment on Scope Pages with a right click over the MEP of FDI.

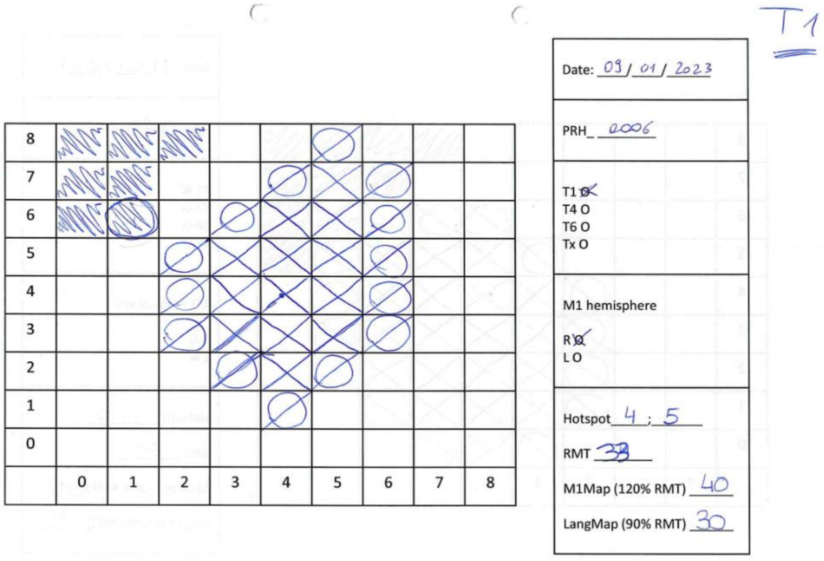

Figure 31. Example of logbook for TMS motor mapping.

Step 10. Once TMS motor mapping has been completed on both hemispheres, start language mapping. Before each stimulation session, show the images to ensure that the patient can name them correctly. Because each session maps a grid of 25 targets at the level of Broca's or Wernicke's areas, it is recommended to create folders of 25 images, and train the patient to name correctly just the images that will be showed pseudo randomly during language mapping.

Each grid is mapped two to three times, to increase the sensitivity of the test.

Part 3. After the patient exits the TMS lab.

Step 1. Save Brainsight project (it is recommended to save it routinely during the whole session). On the page 'Session' of Brainsight select the session of TMS mapping and click on 'Review'. In the left bottom corner of the window select 'Export' to download the recorded samples in MNI coordinates, as .txt file. Upload the text file and the Brainsight project on Microsoft Teams, as back-up copy accessible from a cloud storage.

Step 2. Save Labchart file. On Labchart, open 'Scope Pages', select the time interval where MEPs are elicited, then select 'Multiple Add to Data Pad' by using the function 'time' and stepping through all scope pages. This means that data will be added to data pad based on the time selection (Figure 32).

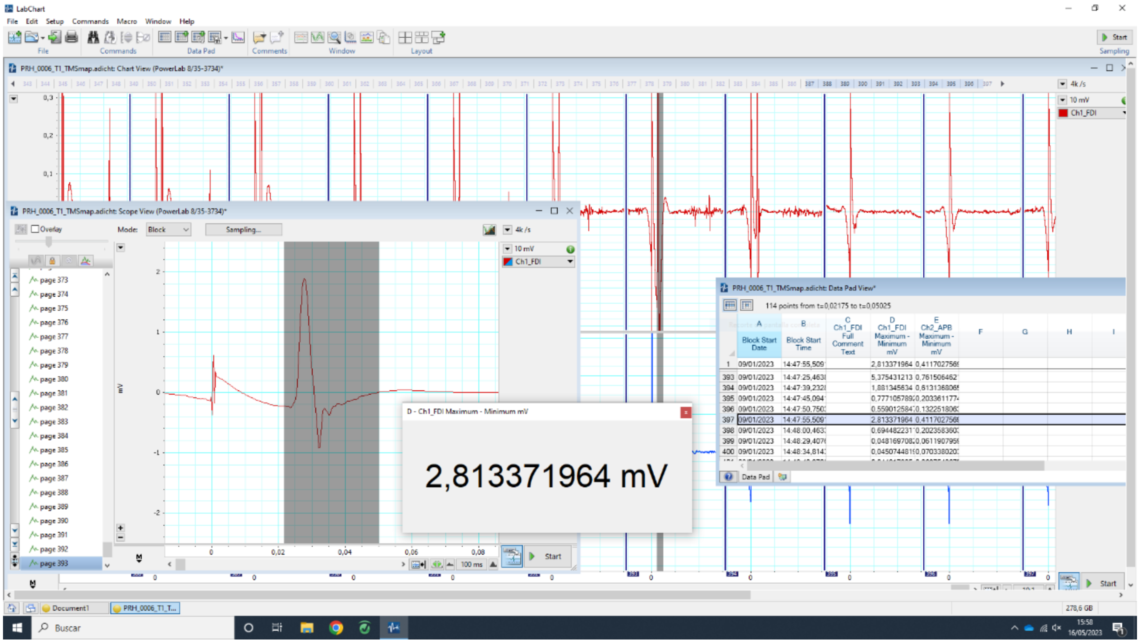

Figure 32. Adding data on Data Pad.

Step 3. Once multiple add to Data Pad is completed, save the latest file version and export two text files, the first one being related to the whole file (Figure 33) and the second one being related to Data Pad only (Figure 34). Upload on Microsoft Teams the two text files, together with the original LabChart file.

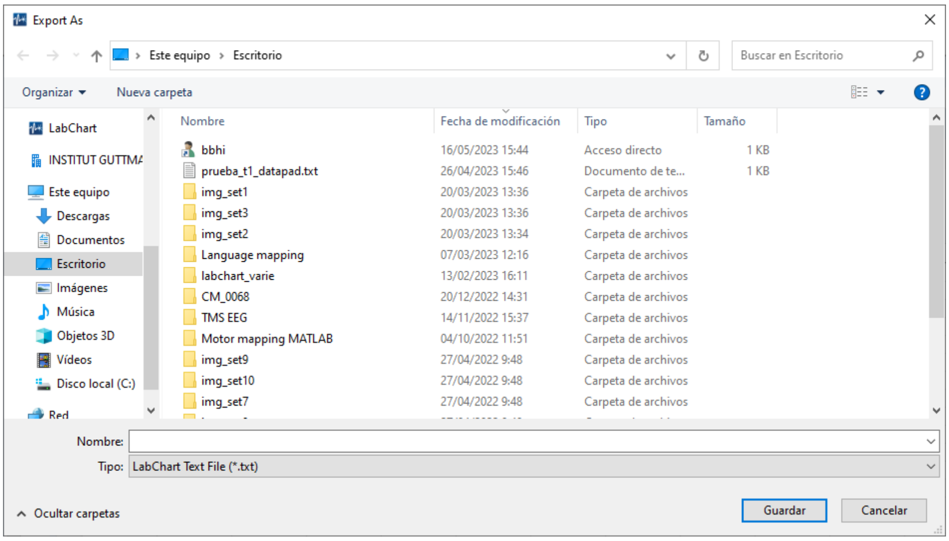

Figure 33. Exporting the whole LabChart file as text file.

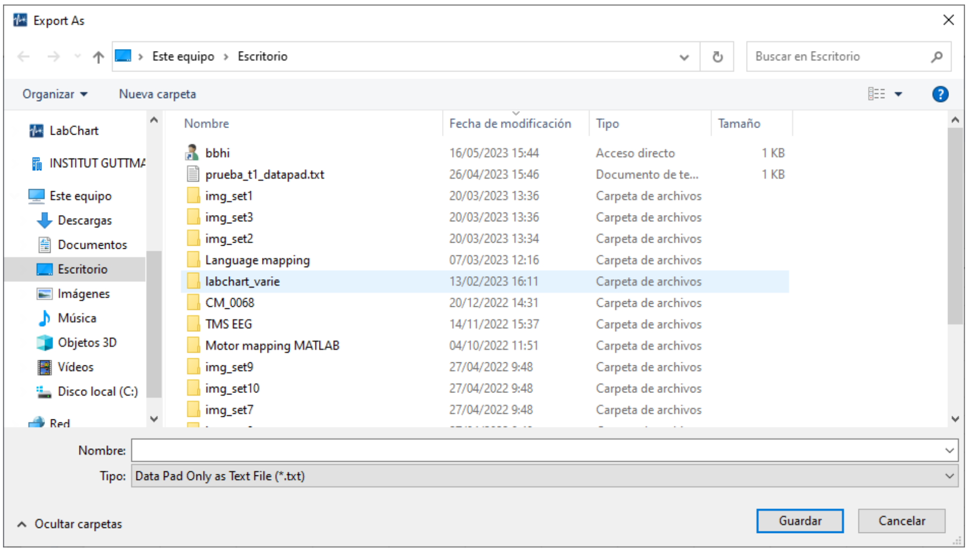

Figure 34. Exporting Data Pad Only as text file.

Step 4. The automatic output from language mapping is a list of audio files (4 seconds duration) recording patient's verbal response to image presentation, together with an excel file listing information of each stimulus event. Files are located the folder 'results' and ordered in subfolders, one for each language mapping session. It is recommended to also upload these files on Microsoft Teams.

A final note: when performing subsequent TMS mapping sessions, it is recommended to use the same stimulation intensity of the first time, to allow comparison between sessions. Therefore, the whole process of identifying hotspot and determining RMT is not performed, which significantly shorten the timing of subsequent mapping sessions.

2.7. TMS motor mapping analysis.

The main two outcomes from TMS motor mapping are the determination of the weighted Centre of Gravity (wCoG) and the creation of a 'heat map' representing the neuroanatomical distribution of motor mapping based on the amplitude of motor response (Figure 35).

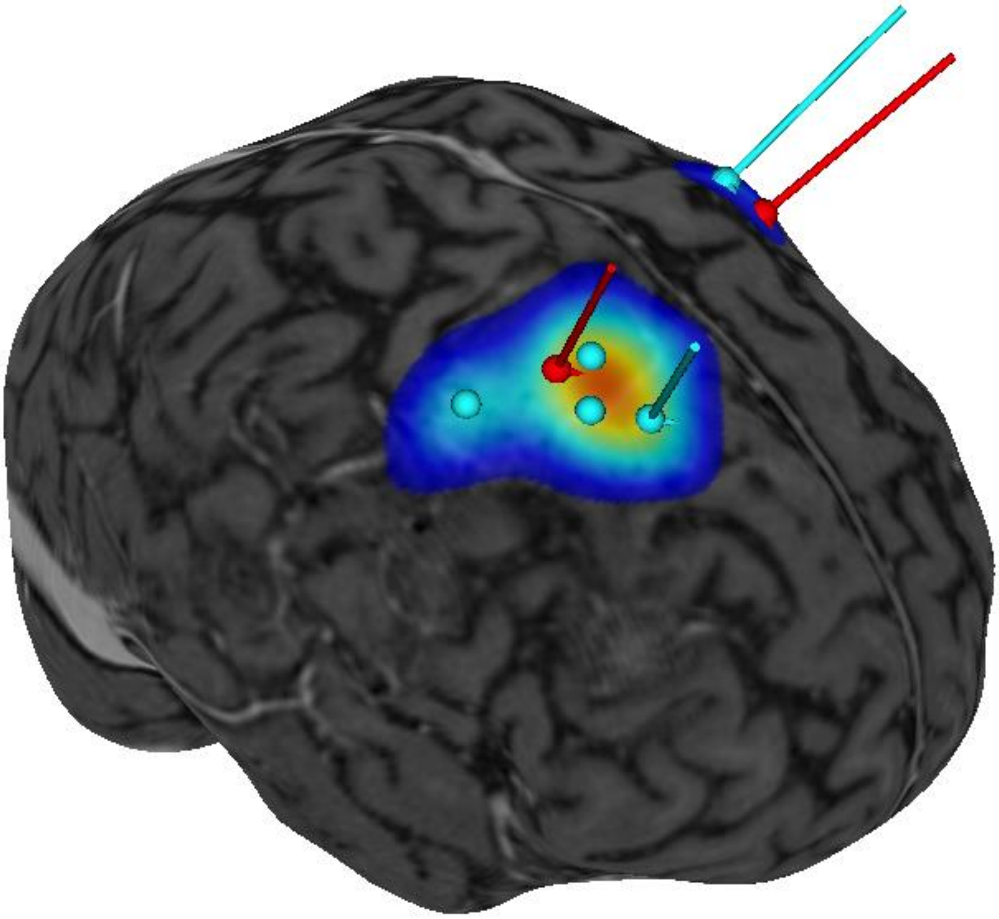

Figure 35. Example of outcomes from TMS motor mapping analysis. Azure spheres are targets with particularly large motor response (the one on the left corresponds with the hand knob, while those on the right are in the premotor cortex). The azure sphere with a stick is the M1 hotspot. The red sphere with a stick is the weighted Centre of Gravity. The heat map underneath is derived from motor mapping analysis, with red colour representing areas of large motor response, and blue areas the borders with no motor response. This figure is also an example of unexpected results (hotspot in the premotor cortex instead of M1) presumably due to neuroplastic changes secondary to the presence of the tumour (supplementary motor area).

Here is the stepwise process for TMS motor mapping analysis:

Step 1. Upload on an excel file (separate sheets) the text files from Brainsight and LabChart, containing samples of the cortical sites that have been stimulated and the peak-to-peak motor response, respectively.

Step 2. Within the same excel file, create a third sheet to pair the list of samples and the list of motor responses. Notably, the timing of samples and the timing of motor responses should match (day, hour, minute, seconds, tenth of seconds).

Step 3. Calculate the average motor responses (usually five) for each cortical site.

Step 4. Convert the average motor response in milliVolts.

Step 5. Report on a separate excel data sheet the list of cortical sites, the associated average response, and the coordinates in MNI space.

Step 6. Determine the three coordinates of the weighted centre of gravity, as follows:

$$\bar{x} = \frac{\sum x_i P_i}{\sum P_i}$$

$$\bar{y} = \frac{\sum y_i P_i}{\sum P_i}$$

$$\bar{z} = \frac{\sum z_i P_i}{\sum P_i}$$

Being *i* one of the cortical targets within M1 grid, x_i y_i z_i are its x,y,z coordinates in MNI space, and P_i is the average peak-to-peak amplitude resulting from stimulation of target *i*. The coordinates of the weighted centre of gravity are the result of the ratio between the sum of all products between target coordinate and peak-to-peak amplitude, and the sum of the average peak-to-peak amplitude of all targets.

On excel, you may calculate wCoG coordinates as depicted in Figures 43, 44, 45. In particular:

- Column A: cortical target name.
- Column B: coordinate x of the cortical target.
- Column C: coordinate y of the cortical target.
- Column D. coordinate z of the cortical target.
- Column E: average peak-to-peak amplitude of the cortical target.
- Column F: sum of all average peak-to-peak (formula in F3: =SUM(E3:E72)).
- Column G: product between coordinate x and the corresponding peak-to-peak (formula in G3: =(B3*E3)).
- Column H: sum of all products in column G (formula in H3: =SUM(G3:G72)).
- Column I: product between coordinate y and the corresponding peak-to-peak (formula in I3: =(C3*E3)).
- Column J: sum of all products in column I (formula in J3: =SUM(I3:I72)).
- Column K: product between coordinate z and the corresponding peak-to-peak (formula in K3: =(D3*E3)).
- Column L: sum of all products in column K (formula in L3: =SUM(K3:K72)).
- Column N: wCoG coordinate x, resulting from the ratio of the value of column H and F (formula in N3 =H3/F3).
- Column P: wCoG coordinate y, resulting from the ratio of the value of column H and F (formula in P3 =J3/F3).
- Column R: wCoG coordinate z, resulting from the ratio of the value of column H and F (formula in R3 =L3/F3).

N15

	A	B	C	D	E	F
1						
2	target	x (coordinate1)	y (coordinate2)	z (coordinate 3)	P (p-p amplitude)	sommatoria P
3	M1_L (5, 5)	-25.7141	-15.8853	68.4319	39	1934
4	M1_L (3, 2)	-58.4813	-21.8017	44.6741	40	
5	M1_L (3, 7)	-23.2017	10.1869	62.0913	43	
6	M1_L (2, 4)	-48.9929	-3.4603	49.5615	44	
7	M1_L (2, 6)	-36.5942	9.9386	56.5295	44	
8	M1_L (2, 3)	-55.8037	-9.9253	45.1063	46	
9	M1_L (5, 6)	-16.9876	-11.3001	70.2953	47	
10	M1_L (5, 3)	-39.5414	-31.0234	60.4053	49	
11	M1_L (4, 2)	-51.7288	-30.813	51.5402	49	
12	M1_L (4, 7)	-17.2617	3.694	65.2015	50	
13	M1_L (4, 5)	-32.6316	-9.4469	64.5793	54	
14	M1_L (1, 5)	-46.898	8.9718	45.3614	57	
15	M1_L (4, 6)	-24.8434	-3.1498	64.9767	61	
16	M1_L (5, 4)	-32.7416	-24.2078	64.2118	65	
17	M1_L (3, 6)	-31.0924	3.6694	60.5594	80	
18	M1_L (2, 5)	-42.9741	3.124	53.2554	87	
19	M1_L (4, 3)	-50.6219	-20.9991	55.4801	122	
20	M1_L (3, 3)	-52.186	-16.0267	53.1555	156	
21	M1_L (3, 5)	-38.3527	-3.1382	58.3247	191	
22	M1_L (3, 4)	-45.1634	-10.2035	57.4364	244	
23	M1_L (4, 4)	-39.9236	-16.7901	62.5936	366	

Figure 36. First part of wCoG calculation.

E	F	G	H	I	J	K	L
P (p-p amplitude)	sommatoria P	xP	sommatoria xP	yP	sommatoria yP	zP	sommatoria zP
39	1934	-1002.8499	-78385.5628	-619.5267	-20096.7919	2668.8441	112730.698
40		-2339.252		-872.068		1786.964	
43		-997.6731		438.0367		2669.9259	
44		-2155.6876		-152.2532		2180.706	
44		-1610.1448		437.2984		2487.298	
46		-2566.9702		-456.5638		2074.8898	
47		-798.4172		-531.1047		3303.8791	
49		-1937.5286		-1520.1466		2959.8597	
49		-2534.7112		-1509.837		2525.4698	
50		-863.085		184.7		3260.075	
54		-1762.1064		-510.1326		3487.2822	
57		-2673.186		511.3926		2585.5998	
61		-1515.4474		-192.1378		3963.5787	
65		-2128.204		-1573.507		4173.767	
80		-2487.392		293.552		4844.752	
87		-3738.7467		271.788		4633.2198	
122		-6175.8718		-2561.8902		6768.5722	
156		-8141.016		-2500.1652		8292.258	
191		-7325.3657		-599.3962		11140.0177	
244		-11019.8696		-2489.654		14014.4816	
366		-14612.0376		-6145.1766		22909.2576	

Figure 37. Second part of wCoG calculation.

L	M	N	O	P	Q	R
sommatoria zP		Xcog (coordinata1 del CoG)		Ycog (coordinata2 del CoG)		Zcog (coordinata3 del CoG)
112730.698		-40.53028066		-10.39130915		58.28888211

Figure 38. Third part of wCoG calculation.

Step 7. Creation of the heat map on Brainsight – insert values. From the main menu open Session > select the session of TMS motor mapping and click 'Review'. Click on 'Config Columns' (Figure 40) and select 'EMG Ch 1', which should return a blank column (n/a). By clicking on each cell of the EMG column, it should be possible to fill the blank cell manually, with the value of the average peak-to-peak in milliVolts. Insert the value only in the last sample of the five samples recorded on the same cortical site.

Step 8. Creation of the heat map on Brainsight – finalize. Click on the icon with an 'i' within a blue circle to open the Inspector window. In the inspector, select 'MotorMaps' and adjust the setting as needed (threshold, opacity,…) to correctly visualize the heat map (Figure 39).

Step 9. Repeat the process for the other side, to allow inter-hemispheric comparisons.

Step 10. Repeat the process at the end of prehabilitation, to allow pre-post comparisons.

A final note for TMS language mapping: because each target is assigned a binary code (positive, in case of speech disturbance during TMS stimulation; negative, in case of normal verbal response despite TMS stimulation) there is no mathematical processing afterwards. To better visualize the results on Brainsight, you may create markers in correspondence to the positive targets, and maybe overlay language mapping with fMRI clusters for language function (Figure 40).

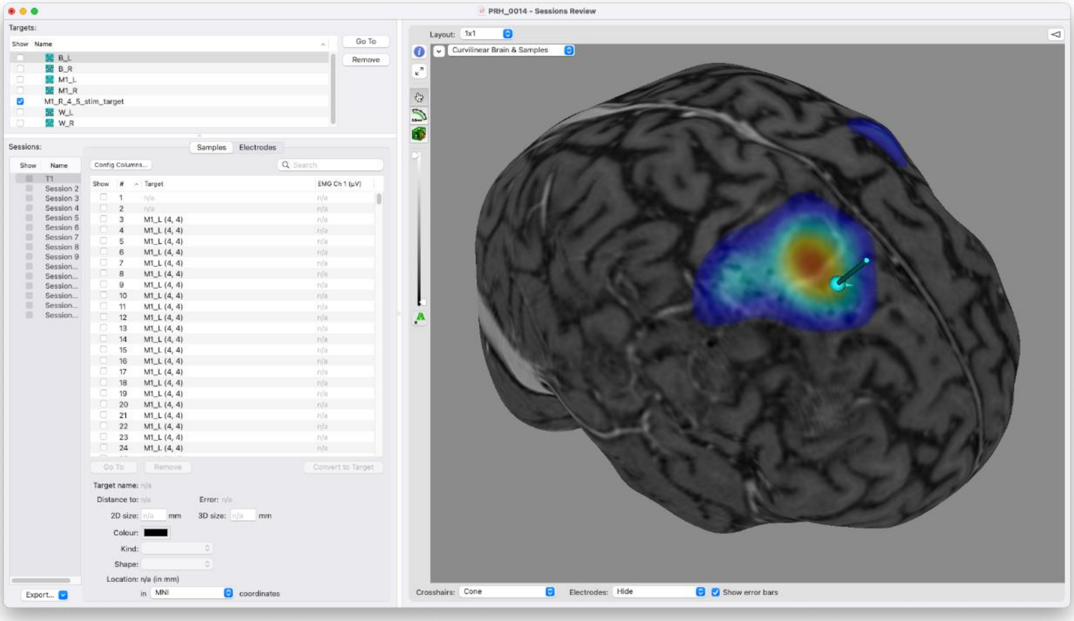

Figure 39. Creation of heat map for TMS motor mapping.

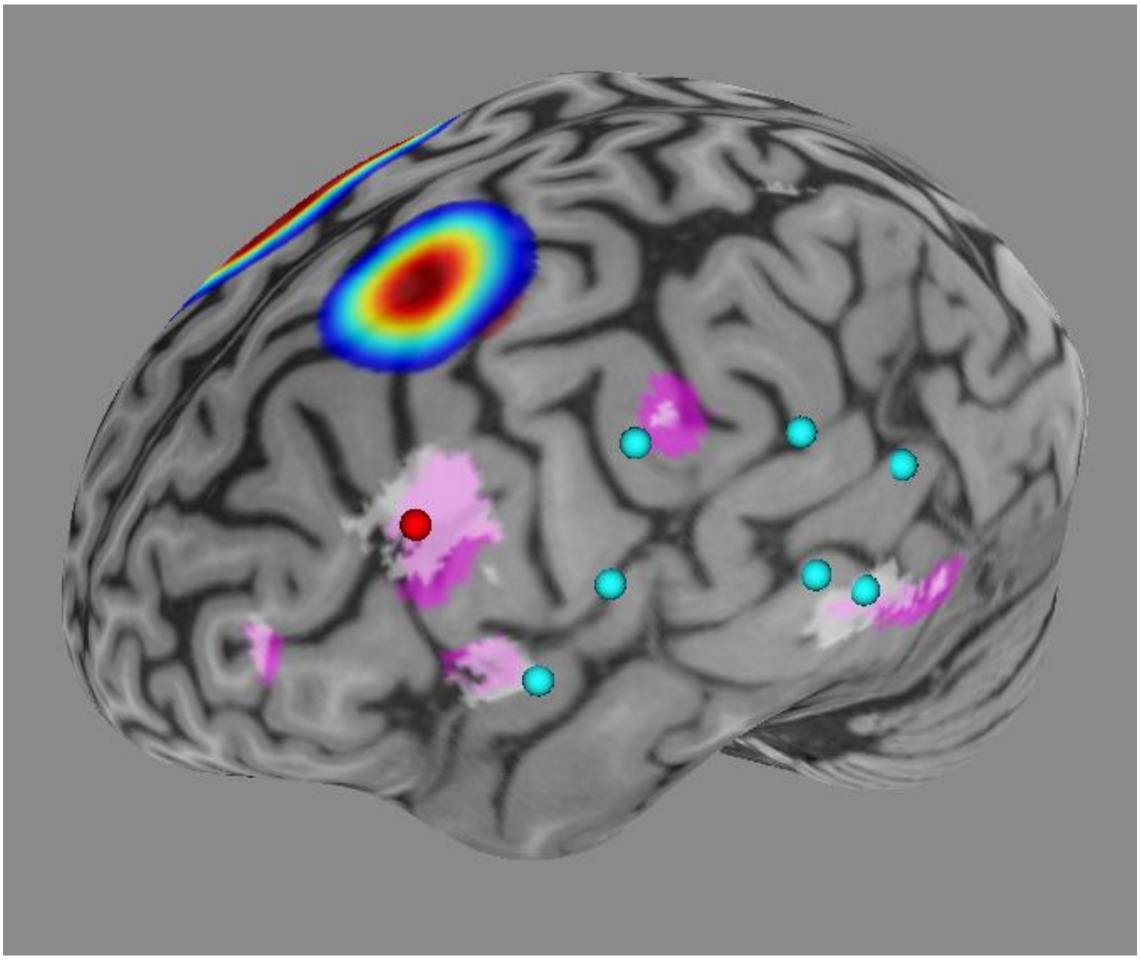

Figure 40. Positive targets for language mapping over fMRI cluster for language function. Azure spheres are for targets that resulted positive only once, while the red target was positive twice.

3. Neuroimaging: main data of interest.

Neuroimaging acquisition is performed at the Hospital Clinic de Barcelona. For each patient, MRI files are stored on a database that is accessible with credentials (username and password).

Files are organized in folders, as follows:

- main folder: patient's study ID (example: PRH_0009).

- subfolders of the main folder: study timepoint (example: PRH_0009_00, PRH_0009_01, PRH_0009_02).

- within each study timepoint: two folders, one for 'Results' (processed data and nifti files), and the other for the original DICOM.

- within the 'Results' folder: the structural MRI in native space (T1w_MPR_...) and MNI space (pwmT1w_MPR_...); eight folders for each of the eight fMRI acquisitions (Figure 41).

- within each fMRI acquisition folder: the nifti files with the fMRI clusters (in native and MNI space), and a pdf file with statistics and the MNI coordinates of the peak-fMRI for each cluster (Figure 42 and 50).

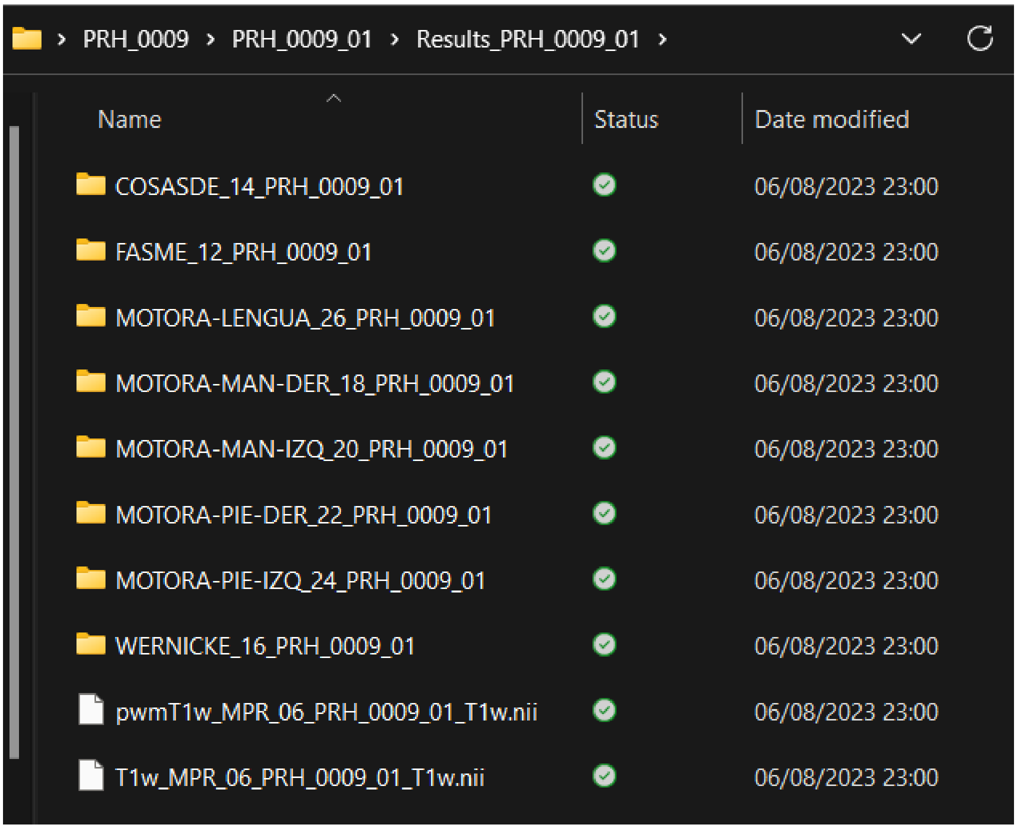

Figure 41. Files within the 'Results' folder.

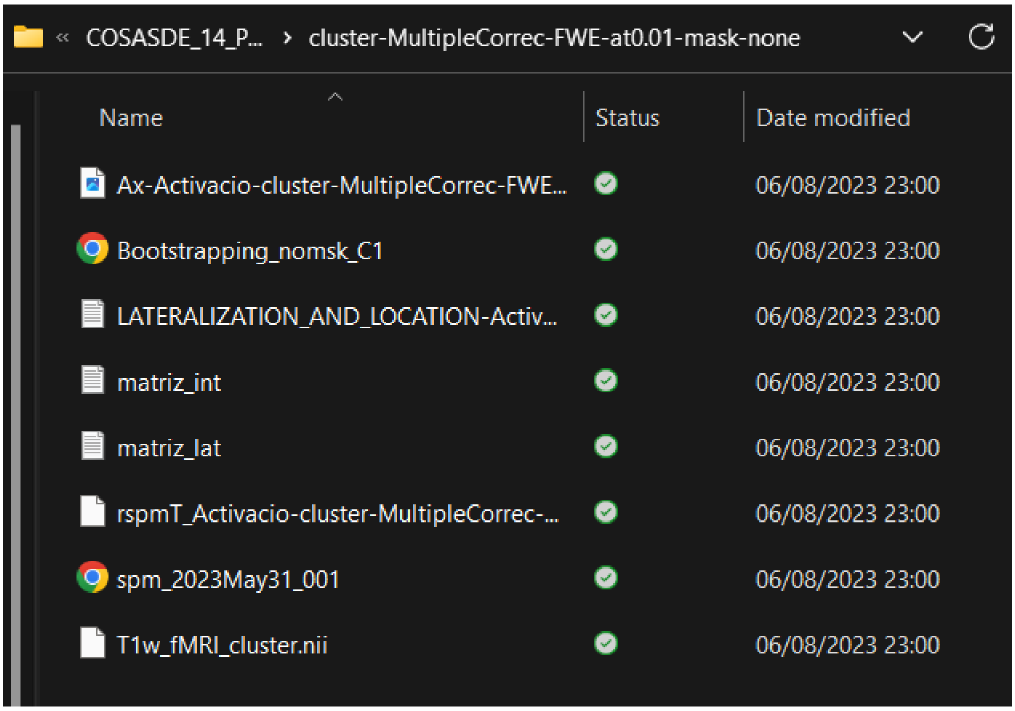

Figure 42. Files within each fMRI folder (in this case, 'COSASDE_14_PRH_0009'). The fMRI clusters in native space are in the nifti file 'T1w_fMRI_cluster.nii', while the fMRI clusters in MNI space are in the nifti file 'rspmT_Activacio-...'). The pdf file with statistics is named 'spm_...'.

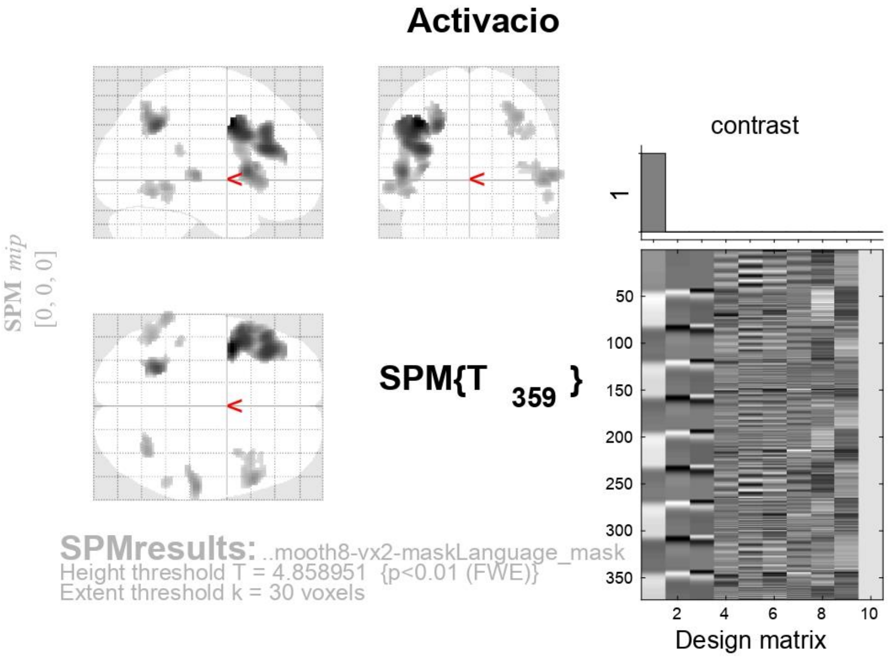

Statistics: ***p-values adjusted for search volume***

set-level		cluster-level				peak-level							
p	c	$p_{FWE-corr}$	$q_{FDR-corr}$	k_E	p_{uncorr}	$p_{FWE-corr}$	$q_{FDR-corr}$	T	(Z_E)	p_{uncorr}	mm	mm	mm
0.000	9	0.000	0.000	1973	0.000	0.000	0.000	12.21	Inf	0.000	-40	2	40
						0.000	0.000	10.26	Inf	0.000	-38	30	20
						0.000	0.000	9.74	Inf	0.000	-48	28	32
		0.000	0.000	298	0.000	0.000	0.000	9.51	Inf	0.000	-26	-54	38
						0.000	0.000	7.39	7.12	0.000	-26	-62	46
		0.000	0.000	140	0.000	0.000	0.000	7.35	7.09	0.000	60	-26	2
		0.000	0.000	273	0.000	0.000	0.000	7.30	7.04	0.000	54	18	-2
						0.000	0.016	5.84	5.71	0.000	36	16	10
						0.004	0.403	5.08	4.99	0.000	40	24	4
		0.000	0.000	147	0.000	0.000	0.000	6.86	6.64	0.000	34	-62	44
						0.000	0.001	6.46	6.28	0.000	28	-62	50
						0.000	0.011	5.94	5.80	0.000	30	-64	32
		0.000	0.000	222	0.000	0.000	0.000	6.69	6.49	0.000	-62	-48	-8
						0.000	0.001	6.54	6.36	0.000	-48	-58	-10
		0.000	0.027	32	0.013	0.000	0.011	5.92	5.78	0.000	-44	-38	20
		0.000	0.001	84	0.000	0.000	0.023	5.75	5.62	0.000	36	6	34
						0.000	0.030	5.69	5.57	0.000	44	16	34
		0.000	0.011	44	0.005	0.000	0.023	5.75	5.62	0.000	56	-46	-14

table shows 3 local maxima more than 8.0mm apart

Height threshold: T = 4.86, p = 0.000 (0.010)
Extent threshold: k = 30 voxels, p = 0.016 (0.000)
Expected voxels per cluster, <k> = 4.734
Expected number of clusters, <c> = 0.00
FWEp: 4.453, FDRp: 5.579, FWEc: 1, FDRc: 32

Degrees of freedom = [1.0, 359.0]
FWHM = 10.9 10.8 10.7 mm mm mm; 5.4 5.4 5.4 {voxels}
Volume: 364944 = 45618 voxels = 224.0 resels
Voxel size: 2.0 2.0 2.0 mm mm mm; (resel = 158.07 voxels)

Figure 43. Example of pdf file with statistics.

From these files, main data of interest are:

- The structural MRI in native space, to visualize the tumor.
- The structural MRI in MNI space, as basis to add overlays of fMRI clusters in MNI space.
- The pdf file with statistics, to identify the coordinates of the peak-fMRI for main clusters of interest.

MRIcron is a freely available software used to visualize nifti files (Figure 44).

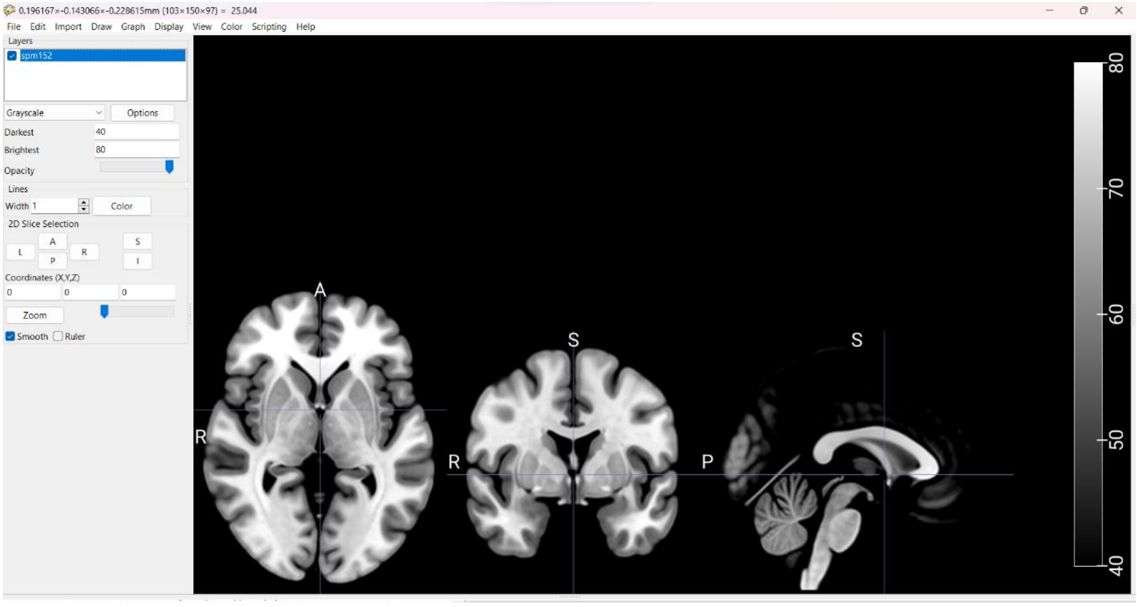

Figure 44. Main window of MRIcron.

From the main window, select ‘File’ > ‘Open’ to select the structural MRI (Figure 45).

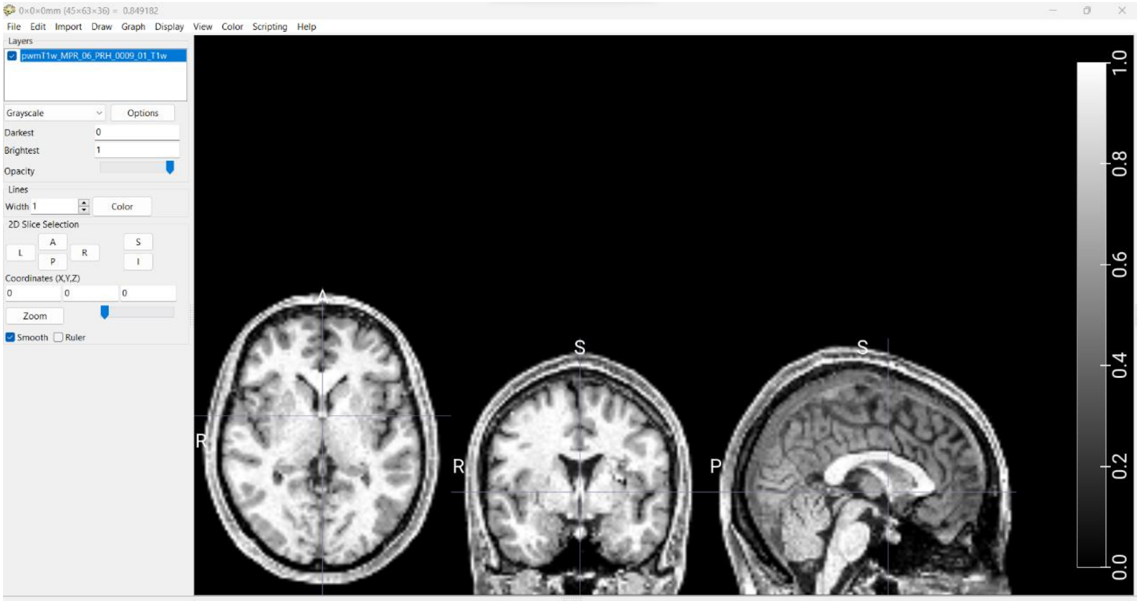

Figure 45. Structural MRI in MNI space.

To add the fMRI clusters, select 'File' > 'Add overlay' (Figure 46).

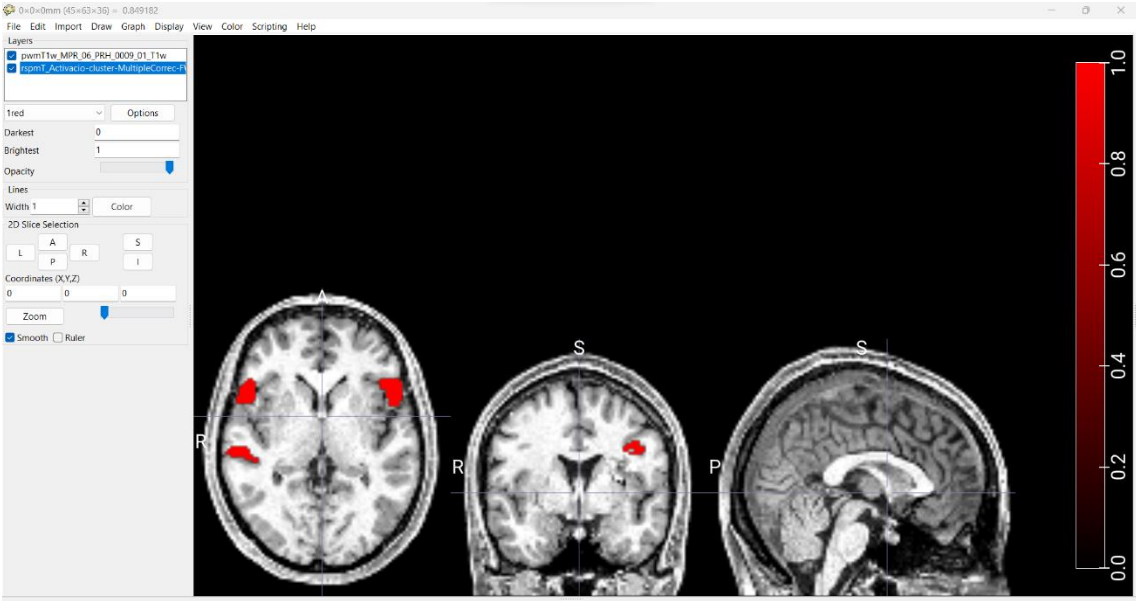

Figure 46. fMRI clusters added as overlay.

To visualize the number of clusters, the volume of each cluster (number of voxels), and the coordinates of the centre of mass, click on 'Options' > 'Generate Cluster Table' (Figure 47).

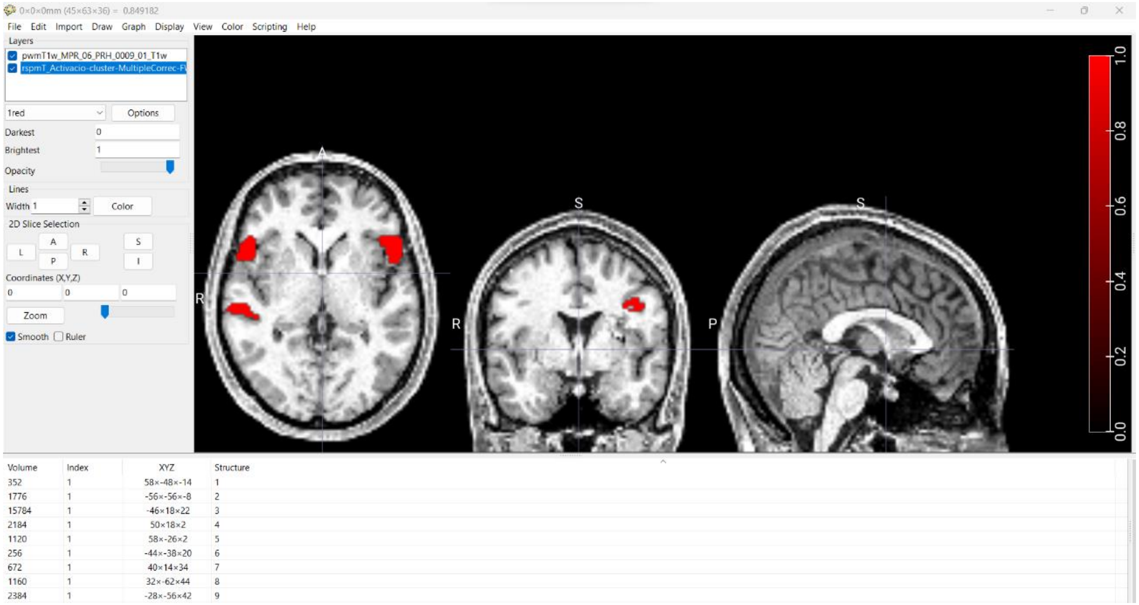

Figure 47. Cluster Table on the bottom of the screen.

By clicking on 'Volume' in the cluster table, it is possible to order the list of clusters based on the volume (from the smallest to the biggest, or from the biggest to the smallest).

Now it's time to check visually which clusters are the closest to the tumor. To do so, click on the corresponding row within the cluster table, which will automatically display the pointer at the centre of mass for the selected cluster (Figure 48).

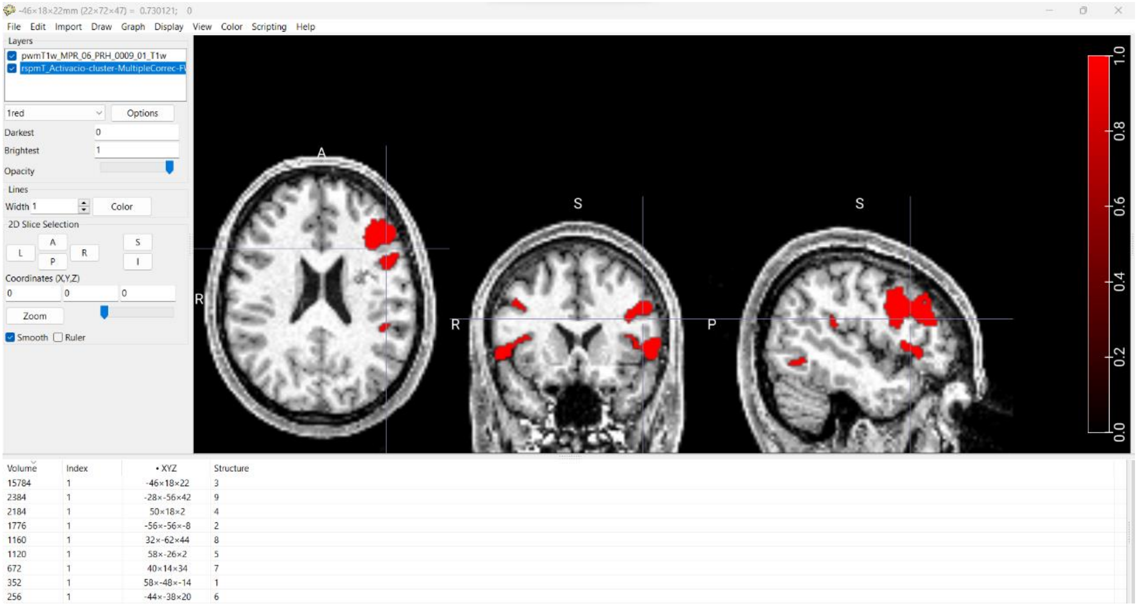

Figure 48. By clicking on the first row of the cluster table, the pointer moved to its centre of mass (-46, 18, 22). This is the cluster of interest because is the largest on the affected hemisphere.

Now it's time to search for the peak-fMRI of this cluster. By looking at the list of peak-fMRI in the pdf file (Figure 43), we identified a potential peak-fMRI with coordinates -40,2,40. To verify that this point is related to the cluster of interest, the coordinates are typed into MRIcron (Figure 49).

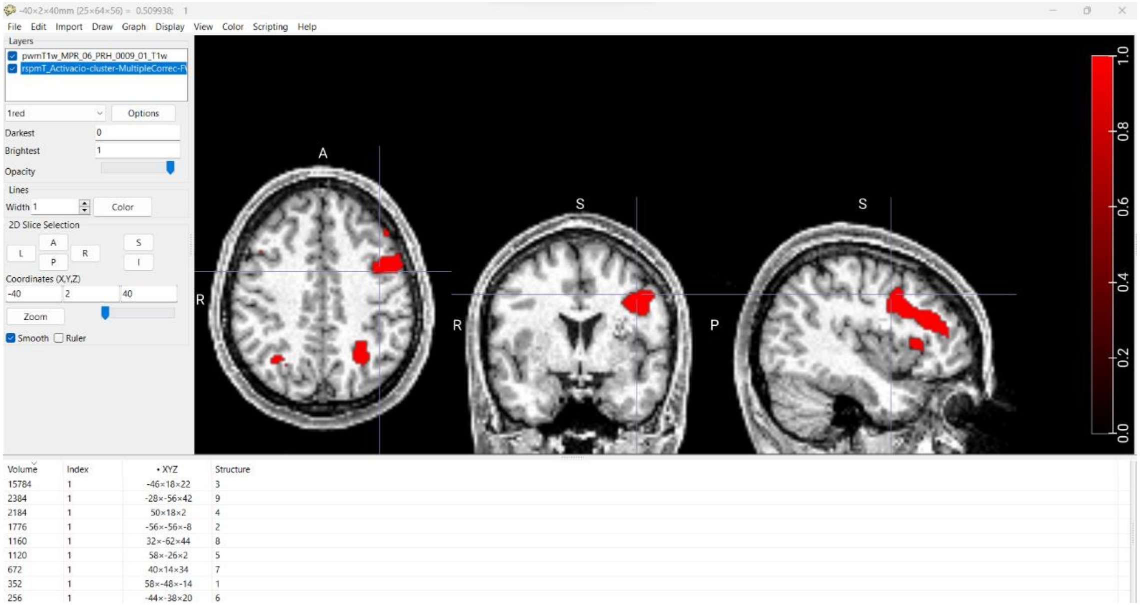

Figure 49. Pointer at coordinates -40, 2,40.

Identifying the main cluster location within each fMRI task with respect with the tumour is the first step to hypothesize which functions are more at risk of being compromised, thus potential goals for prehabilitation.

Peak-fMRI could be the target for neuromodulation, by inserting the coordinates on Brainsight.

4. Treatment.

4.1. Decision making process for treatment planning.

The objective of prehabilitation is to optimize neurosurgical outcomes, by extending the functional margins for safe tumor excision. To do so, functions at risk of being compromised (functional representation close to the tumour) are at the core of both neuromodulation (inhibition of eloquent areas) and subsequent intensive task training (promotion of activation for alternative nodes within the same functional network).

The decision-making process cannot be a rigid algorithm because there are too many factors that should be considered on a case-by-case basis. Instead, we propose a semi-structured method of reasoning leading to optimal, personalized interventions.

There are three steps in the development of the intervention: target selection, neuromodulation parameters, design of task training.

Step 1. Select the target for neuromodulation.

Target selection is the most articulated step, which can be divided into five consecutive questions:

1) Which function(s) is potentially at risk of being compromised?

To answer this question, we should consider tumour location, symptoms, and neurosurgical planning. For instance, if the tumour is located subcortically close to Broca's area then speech production could be a function of interest; this function could have been already altered (presence of symptoms/deficits), although it is also possible that the patient is asymptomatic. Finally, the neurosurgeon may be more worried about other functions (motor function for the hand and the face) because the entry point for surgery passes in proximity of the prerolandic gyrus. Therefore, it is mandatory that at this initial stage there is a multidisciplinary discussion between the neurosurgeon and the neuroscientists involved in the intervention.

As an example, the first ten cases included in the protocol presented with one or more of these functions as target for treatment:

- Motor function (upper limb).

- Motor function (face).

- Speech production.

- Memory.

- Executive function.

2) Is there an fMRI scan available?

After having considered the neuroanatomy and clinical symptoms, a further step is to see the results from fMRI scans. For the present protocol, fMRI investigates language comprehension, speech production, motor function for the upper limb, lower limb, and tongue. If the tumour is located close to areas related to language or motor function, then we should consider the peak-fMRI of the largest cluster ipsilateral to the tumour as target for neuromodulation.

3) Is there a TMS mapping available?

TMS motor and language mapping may also be useful to identify the target for neuromodulation. For TMS motor mapping, the target is the M1 hotspot, while for language mapping it may be one of the targets that resulted positive.

In general, if the target is upper limb motor function we select the M1 hotspot, while if the target is language function we select the peak-fMRI of the largest cluster.

4) Are there standardized coordinates in the literature?

In cases where the targeted function is not related to language or upper limb movement, it is useful to search for standardized coordinates of cortical structures in the literature. For instance, the function at risk could be executive function, because the tumour is underneath the dorsolateral prefrontal cortex. In this case, we will search for coordinates of sub-regions within the dorsolateral prefrontal cortex and select the one with cortical-subcortical correspondence with tumour location (and/or with surgical entry point). Another example is whether the function at risk is memory because of a tumour close to the hippocampus. In this case it is not possible to apply neuromodulation directly to this area; instead, we select the cortical target that has been reported in the literature as functionally related to memory function (in the parietal lobe).

5) Is there a neuroanatomical landmark?

Finally, in case that fMRI and TMS mapping are not available, and standardized coordinates are not applicable (anatomical distortion provoked by the tumour mass), we search visually for anatomical landmarks related to a specific function at risk, to select the target for neuromodulation. This can be done in the structural MRI scan, or even directly in the neuronavigation project.

Step 2. Neuromodulation protocol (TMS/tDCS).

Most of the time, neuromodulation consists of low-frequency rTMS focus on a specific target, with a 1-cm diameter. This is the case for cortical-subcortical tumours where the function at risk may be neuromodulated through one of its main nodes. However, sometimes tumours are located more in-depth and/or more extended than others, posing risks for more than one function and more than one region, or even more than one lobe. In these cases, multifocal tDCS may be used to inhibit extended cortical areas ipsilateral to the tumour (cathodes), while at the same time stimulating the corresponding areas on the contralateral hemisphere (anodes). A third possibility is to perform both TMS and tDCS, for instance with TMS sessions in the morning and tDCS sessions in the afternoon, to benefit from both focused neuromodulation of a specific functional node and broad cortical inhibition.

Step 3. Intensive task training.

Task training should be carefully designed according to the following parameters:

- Specificity. Focus on the function at risk of being compromised.
- Instensity. Considering that we want to provoke durable neuroplastic changes with only 10-20 sessions, and that we should allow practice only in the 60 minutes after neuromodulation (the temporal window when the targeted area has been inhibited), intensity in the sense of number of repetitions should be as high as possible.
- Variability. While holding the focus on a certain function at a certain intensity, allow variability in the type of exercise. The motto is ‘repetitions without repetitions’.

- Difficulty. The level of mental challenge posed by the exercise should at the level of being 'difficult, yet achievable'. If the exercise is too easy the brain will not be forced to recruit alternative resources to accomplish the task. If the exercise is too difficult the task will not be accomplished, or the number of repetitions will reduce significantly.
- Engagement. Consider patient's motivation and level of engagement. This is achieved by creating a therapeutic alliance and coaching during the training, but also by tuning the type of exercise, insert variations and adjust the difficulty in a playful way.

It is important to note that rehabilitation and prehabilitation are two different paradigms, and so is the focus of the intervention and the design of the training. In traditional rehabilitation, we focus on improving functionality (focus on the deficit, or in other words 'what is missing'). In prehabilitation, we focus on leveraging neuroplastic changes to preserve functionality (focus on a potential risk, or in other words 'what is still present, but…'). Therefore, it may be that the patient presents with certain symptoms that would require rehabilitation but are not the focus of prehabilitation. For instance, a patient with a tumour close to primary sensorimotor cortex, somatosensory symptoms (proprioceptive deficits), but with function at risk being upper limb motor function. In this case, the main focus of the training is motor function, not somatosensation.

4.2. Examples of treatments applied so far.

In this final section we provide a succinct description of two cases that were enrolled in the prehabilitation protocol, to give a practical idea of the whole intervention.

Case #1 is a patient with a grade IV frontotemporoinsular glioma with an IDH mutation. During the last three years there were episodic alterations of consciousness, suggestive of epileptic seizures; however, during the whole intervention the patient was asymptomatic. Because of tumour localization, the neurosurgeon indicated speech production as the function at risk of being compromised. Accordingly, fMRI pre-prehabilitation showed the main cluster for semantic decision in proximity with the tumour (Figure 50). Therefore, the peak-fMRI for the main ipsilesional cluster was selected as target for rTMS. After each rTMS session the patient undertook one hour of intensive neurocognitive training with an experienced neuropsychologist. Furthermore, because of the extension of the tumour, tDCS was applied with the cathodes over the ipsilesional parietofrontal cortex (F3, P3, T7, EEG electrodes), and the anode contralaterally on C4 (Figure 51). During each tDCS session, the patient undertook cognitive training with the online program 'Guttmann NeuroPersonalTrainer®'. TMS sessions were performed in the morning, and tDCS sessions in the afternoon. If only one slot was available, TMS was performed. At the end of the last session of the day, the patient undertook a short session of High Intensity Interval Training (HIIT) on a stationary bike, to promote neuroplasticity at molecular level (release of BDNF) and foster learning consolidation. The HIIT protocol was: 5 minutes warm-up, first HIIT bout (30 seconds all-out + 30 seconds rest, 10 times), 5 minutes rest, second HIIT bout (same as the first bout), 5 minutes cool-down.

By the end of the intervention (20 sessions) a second fMRI was performed, showing an increase in the distance between the tumour and the main ipsilesional cluster for semantic decision (Figure 52). The patient then undertook neurosurgery with partial tumour resection, with no neurological sequelae. Post-surgical treatments included radiotherapy and oral chemotherapy.

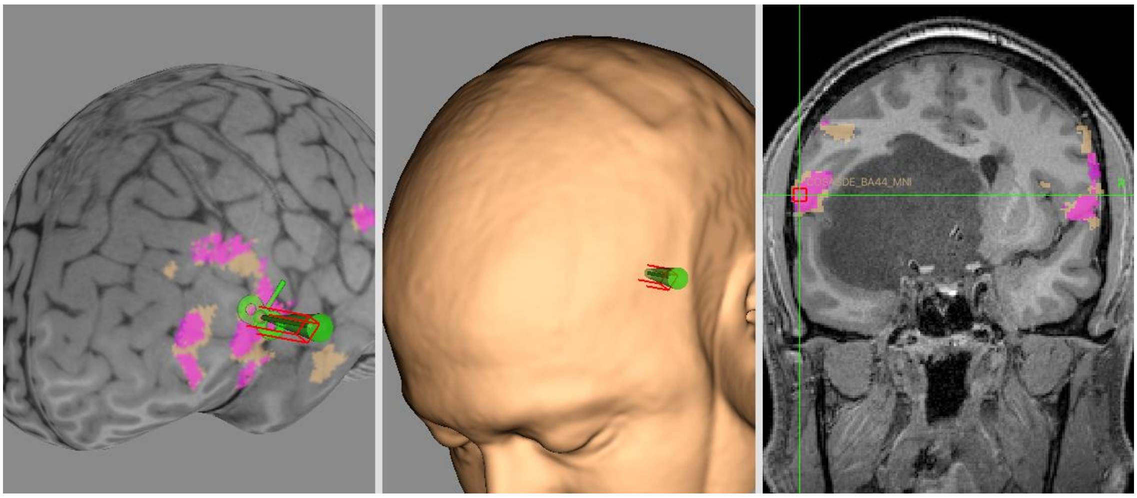

Figure 50. Target for neuronavigated rTMS on peak-fMRI for semantic decision task.

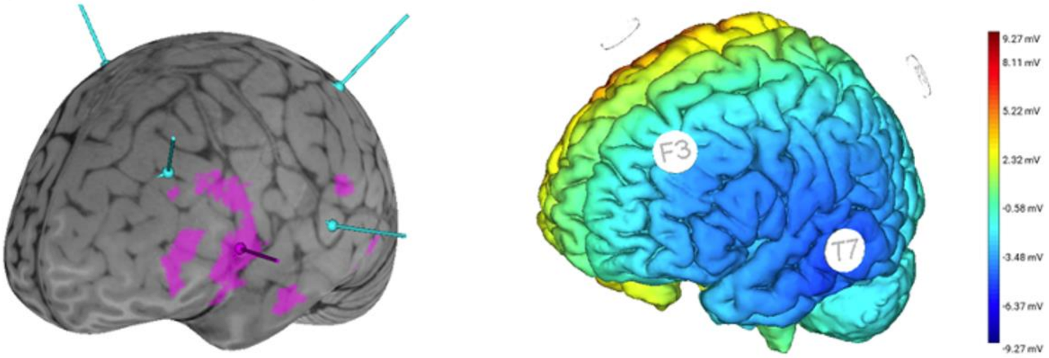

Figure 51. Neuromodulation targets (on the left) depicted in violet (for rTMS) and azure (for tDCS). Multifocal tDCS map (on the right) showing diffuse inhibition over the ipsilesional parietofrontotemporal cortex.

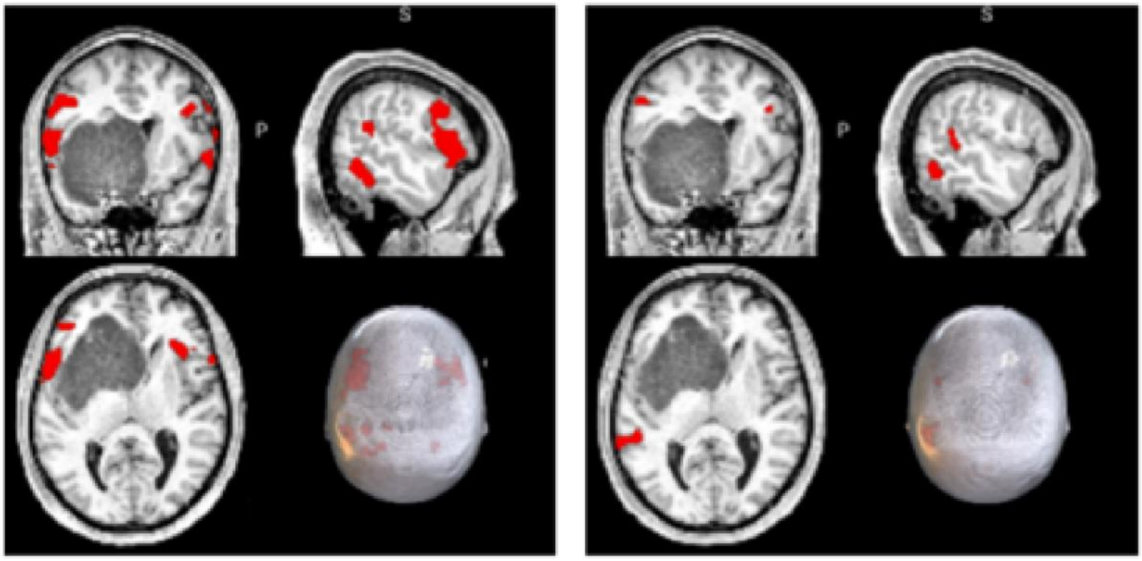

Figure 52. fMRI pre-prehabilitation (left) and post-prehabilitation (right).

Case #2 is a patient with recidivant glioblastoma in correspondence with the right premotor cortex. Before enrolment in the prehabilitation protocol, DTI performed at the hospital showed the posterior border of the tumour in proximity with the corticospinal tract. (Figure 53). At baseline, TMS motor mapping showed a pattern of activation in correspondence with tumour location (Figure 54), with the hotspot on the premotor cortex. Clinically, the patient reported left hemiplegia immediately after the first neurosurgery (about nine months before the inclusion in the prehabilitation protocol) with full recovery, so that at baseline presented with no symptoms. The intervention consisted of 15 sessions of rTMS followed by intensive task training for upper limb (reaching) and hand (finger individuation, dexterity) movements. Additional tasks where the inclusion of cognitive components (dual task), such as complex motor sequence memorization, task switch, stroop task, bimanual coordination, etc. Sessions were performed once-twice a day, and by the end of each session a HIIT was performed (stationary swimming in the swimming pool by an elastic rope attached to the trunk and fastened to the border of the pool) similarly to what described for case #1.

By the end of the prehabilitation protocol, a second TMS motor mapping showed that the pattern of activation was displaced laterally and posteriorly. A DTI based on positive targets from TMS mapping showed that there were additional fibres of the corticospinal tract laterally and posteriorly (Figure 55), as compared to the first DTI reconstruction.

The neurosurgeon performed gross total resection (Figure 56), with no sequelae postoperatively.

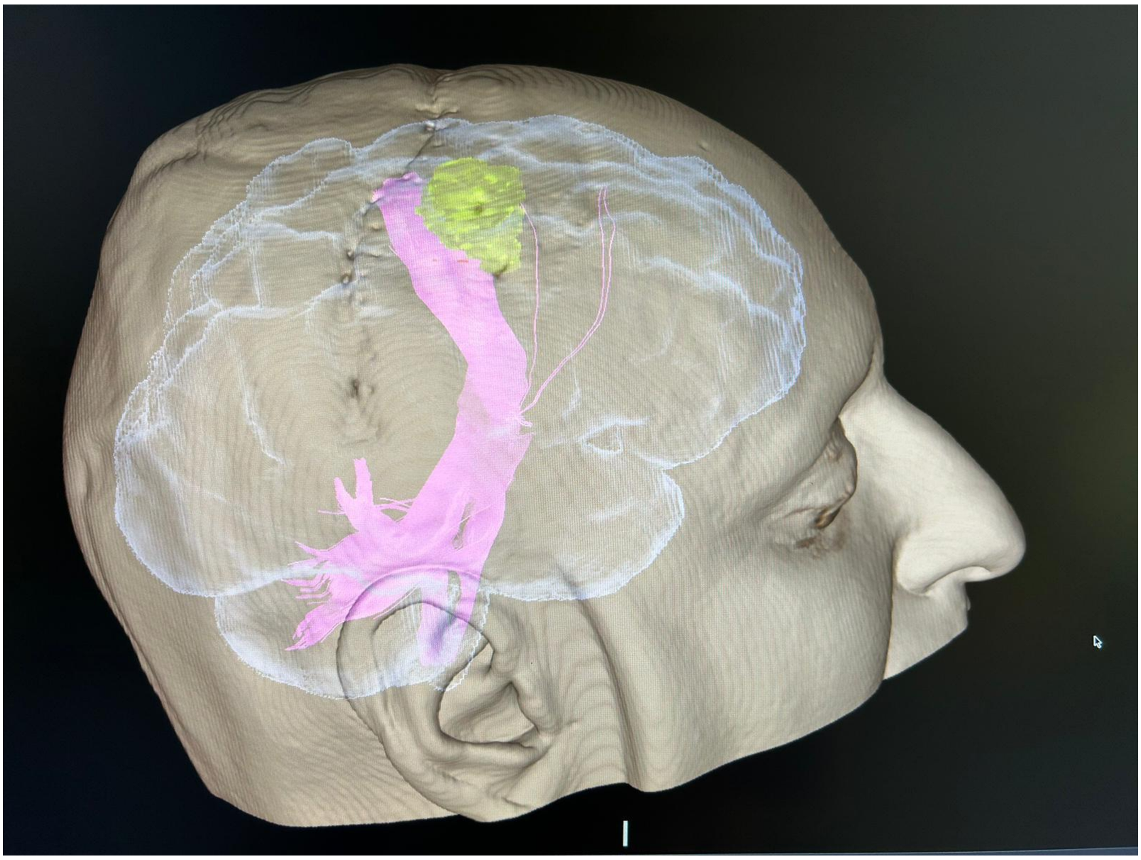

Figure 53. Initial DTI reconstruction (before inclusion in the prehabilitation protocol). The corticospinal tract (pink) is posterior to the tumour (yellow).

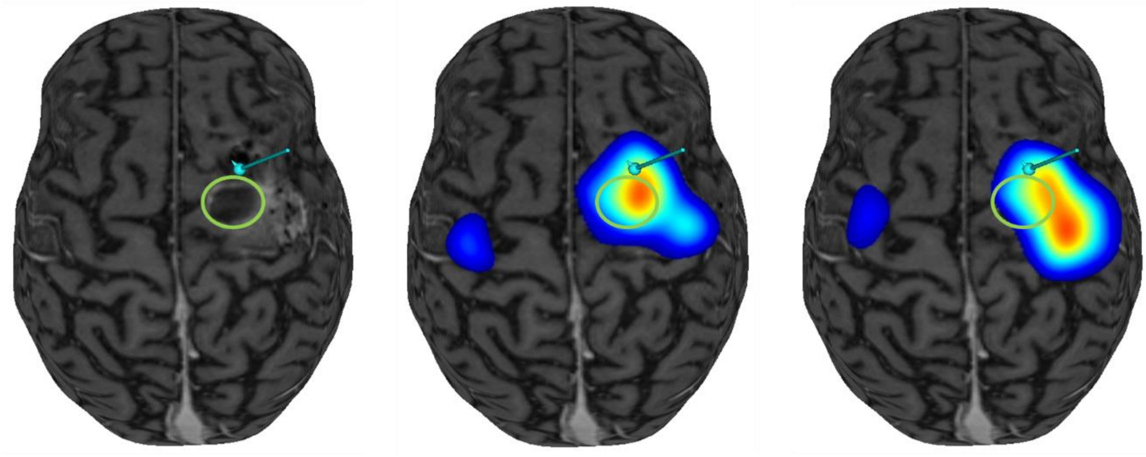

Figure 54. TMS motor mapping pre-prehabilitation (centre) and post-prehabilitation (right). Tumour location is delimited by the green circle; rTMS was applied on the azure target.

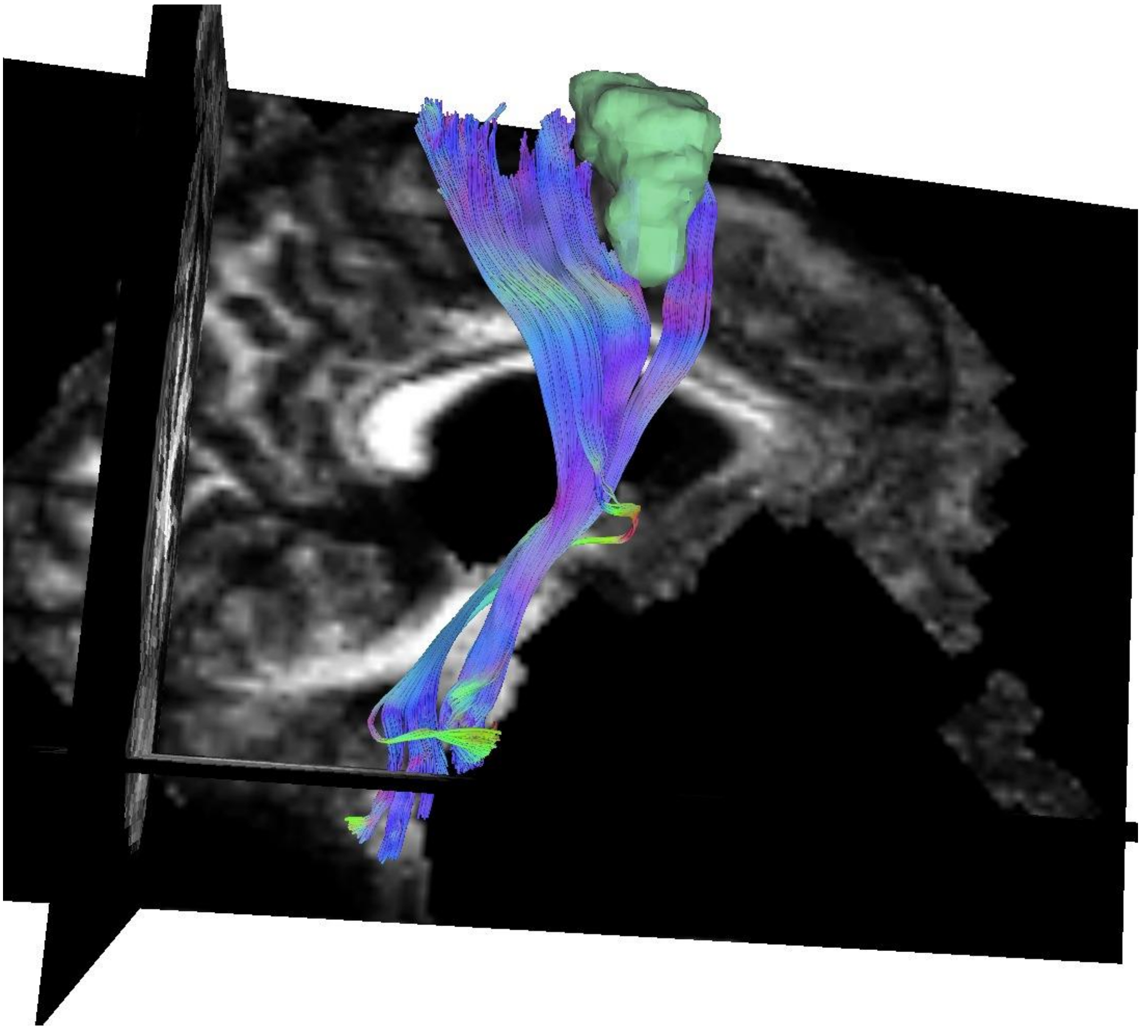

Figure 55. DTI reconstruction post-prehabilitation, based on targets from TMS motor mapping, showing additional fibres composing the corticospinal tract laterally and anteriorly.

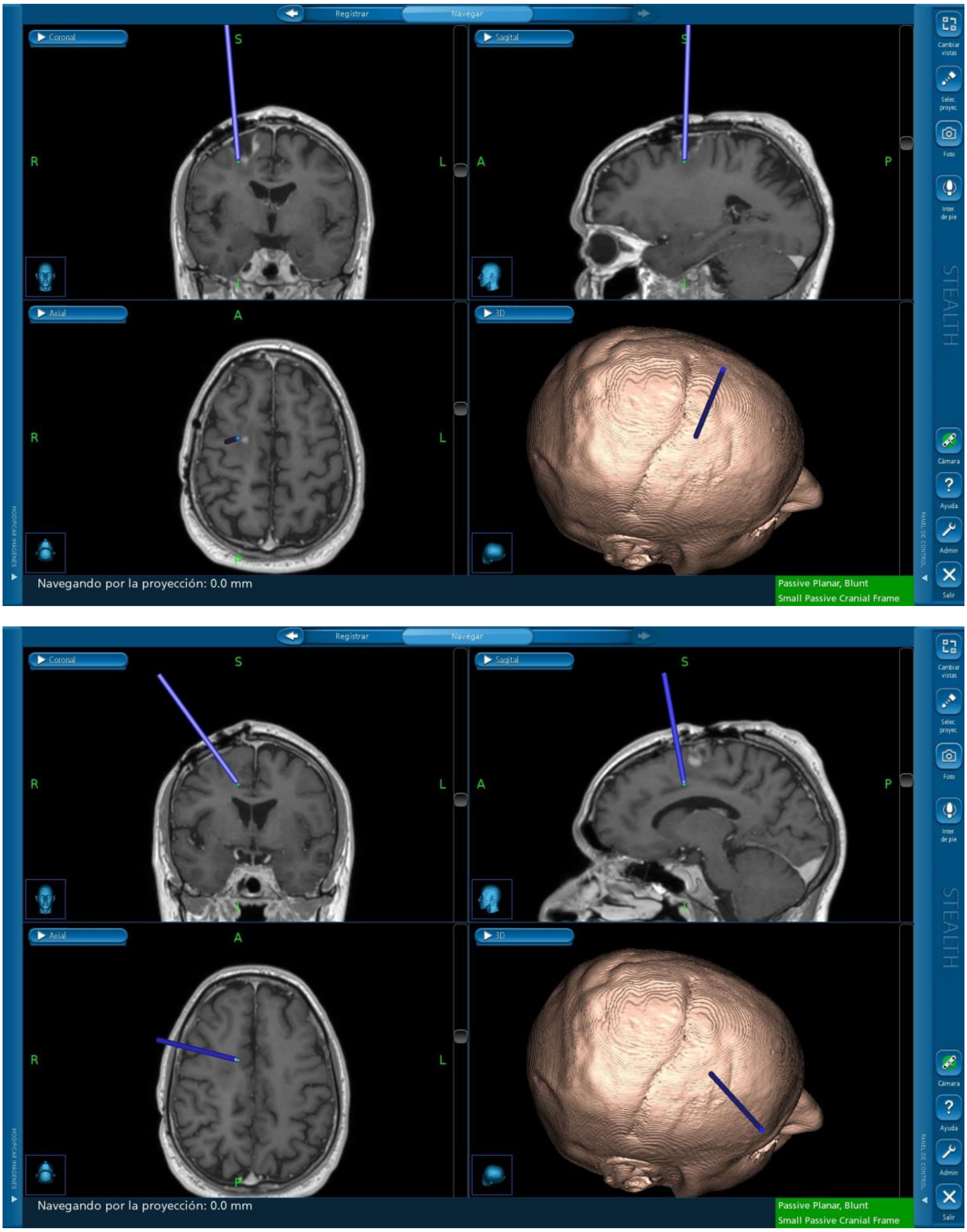

Figure 56. Intra-operatory neuronavigation with the pointer showing the extent of resection margins.

Appendix.

1. Summary of steps needed to create a neuronavigation project using Brainisight.

2. Printable logbook for a TMS mapping session.

3. Matlab script for language mapping.

4. Study protocol.

Appendix 1. Summary of steps needed to create a neuronavigation project using Brainsight.

Step 1: Loading Anatomical Images.

Definition: upload of static MRI data.

Rationale: basis of coordinate system onto which all data is registered to.

Substeps:

1. Launch Brainsight-> **I agree** -> **New empty project.**
2. **Choose** -> select the MRI file -> **Show Image & details** (optional).

Step 2: MNI/Talairach registration.

Definition: co-registration of individual MRI into MNI/Talairach coordinates.

Rationale: only if you wish to input targets based on MNI/Talairach coordinates, or export sampled targets in MNI/Talairach coordinates.

Substeps for manual MNI registration:

1. **Atlas Spaces** -> **New** -> **Manual (AC-PC + Scale).**
2. Scroll the images, then **Set AC** and **Set PC** -> **Next Step.**
3. If tilted, use **Alignment** slider to align (bottom right).
4. Set the size of the bounding box on both coronal and transverse plans.
5. **Update**-> Check the correspondence between Overlay LUT and MRI -> **Finish.**

AC (Letter F) is the anterior commissure, a white matter tract connecting the medial aspect of the two temporal lobes, right in front of the two columns of the fornix.

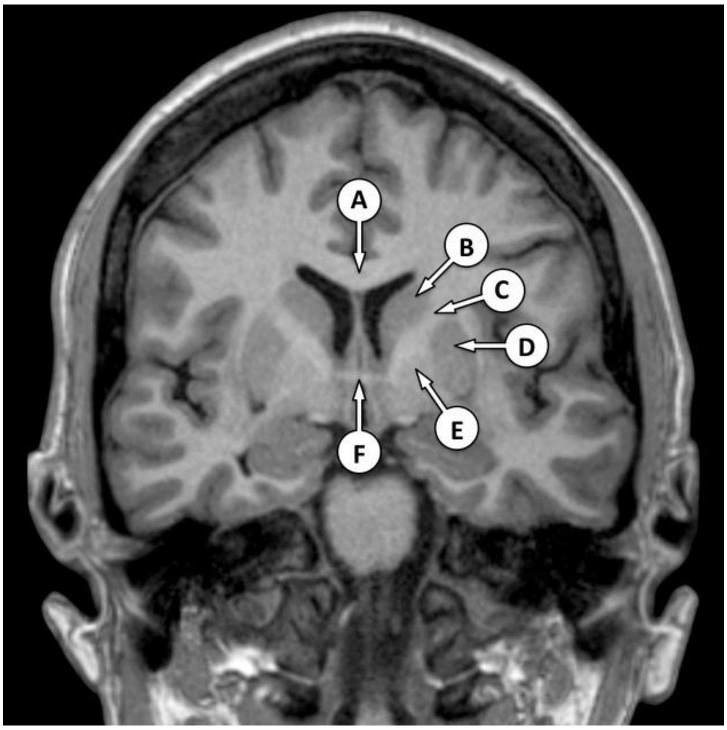

PC is the posterior commissure, a white matter tract running on the dorsal aspect of the rostral end of the cerebral aqueduct.

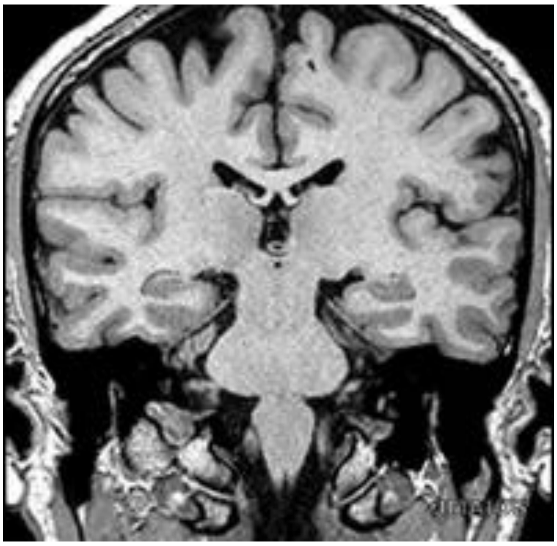

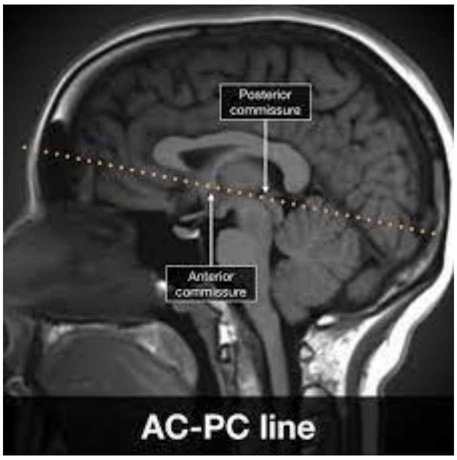

Step 3: Image Overlays

Definition: overlay of fMRI, other structural MRI (T2) or standard Broadman areas.

Rationale: if the overlaid data have meaning for subsequent steps (for instance, target identification).

Substeps:

1. **Configure overlays** -> **Add**…
2. If you upload broadmann template, select **Using atlas spaces** as Registration.
3. If you upload fMRI -> check co-registration:
    a. if functional data were resampled to match anatomical data -> select **None** as Registration.
    b. if there is a header that stores information of registration to anatomical data (as sometimes done for MINC and NIFTI) -> select **from headers.**
    c. if a matrix is used -> select **Explicit Matrix** (check manual).

Step 4: ROI painting.

Definition: labelling ROI.

Rationale: differentiate ROI from surrounding areas not of interest.

Substeps:

1. **ROIs** -> **New ROI.**
2. Set threshold and main image view (suggestion: Transverse).
3. Select on ROI (either clicking on Seed or manually with pencil and fill).
4. Going back and forth with the buttons Next and Previous Slice will fill automatically the same regions identified with the Seed.

Step 5: 3D reconstruction.

Definition: recreating a 3D reconstruction.

Rationale: display purposes and to simplify the identification of markers and targets.

Examples:

- skin reconstruction > easier marker identification.
- brain reconstruction > easier target identification.
- ROI reconstruction > important anatomical/functional areas identification.

Substeps:

1. **New** -> **Skin.**
2. Set bounding boxes to include the whole scalp.
3. **Compute Skin** and close the window.
4. **New** -> **Full Brain Curvilinear.**
5. Set slice spacing and end depth as preferred (default: 1 mm, 16 mm).
6. **Compute Curvilinear** and close the window.
7. If needed, make the same computations for ROI or overlays.
8. If needed, compute surface ROI and overlays.

Step 6: Selecting anatomical landmarks.

Definition: marker identification on 3D skin reconstruction.

Rationale: identify unequivocal head reference points.

Substeps:

1. **Landmarks** -> **Configure landmarks.**
2. Go to Skin & Landmarks and point towards your first landmark (Nasion).
3. Click on New and Name it 'Nasion'.
4. Do the same for the tip of the nose, the left and right notch above tragus, and outer canti of the eyes (if one of the previous is not available) and close the window.

Step 7: Selecting targets for stimulation.

Definition: target identification.

Rationale: based on 3D brain reconstruction, 3D ROI reconstruction or MNI/Talairach coordinates.

Substeps:

1. **Targets** -> **Configure Targets.**
2. Select the Reconstruction you need (Skin, Curvilinear, ROI, Overlays…).
3. Rotate the image until you see clearly your anatomical or functional target.
4. Select the **Target Positioning Tool.**
5. Click on the target.
6. Leave AP and Lat as default; adjust Twist as needed.
7. New -> Select Type of target (note: for Cobot, you would need trajectories).
8. if you select a grid:
    a. select grid size and grid spacing.
    b. **kind** -> select **Trajectory.**
    c. **Snap To…** -> Curvilinear Brain // Skin -> **Snap.**
9. alternative to anatomical based target:
    a. MNI based target: input x y z coordinates.
    b. fMRI based target: just display fMRI by opening the inspector.
    c. target based on previous study:
        i. define a rough target and perform a study.
        ii. record coordinates of relevant targets.
        iii. review data, select the sample and click on **Convert to target.**
        iv. Open the target window again, and click on Move to **Crosshair offset** (target on cortex, rather than on scalp).

Step 8: Performing the study.

Substeps:

1. PREPARE THE TRACKED TOOLS (subject tracker, coil tracker and pointer).
2. PREPARE THE SUBJECT (head orientation and head-strap).
3. PREPARE THE HARDWARE (Magstim, EMG, etc.).
4. BEGIN A NEW TMS SESSION.

- open Brainsight and open the **subject's project file.**
- **New -> Online Session.**
- Add all targets of interest (even more than once if you want to repeat stimulation).
- Click on **Next Step.**

5. CONFIGURE THE I/O OR TRIGGER BOX.
6. VERIFY PROPER POLARIS LOCATION (Polaris verification screen).
7. PERFORM THE SUBJECT-IMAGE REGISTRATION.

- Pointer to reference marker on subject.
- **Sample & Go to the next landmark**...
- Click on **Next Step.**
- On registration verification, check on **Crosshair-> Skin** to verify that scalp and pointer locations match (up to 3 mm error is good).
- **Click on Next Step.**

8. PERFORM THE REGISTRATION USING MNI MODEL.
9. PERFORM THE TMS STIMULATION SESSION.

- select the tool on the crosshair **driver** popup window (in our case, TMS coil).
- select the target of stimulation by clicking on the list of targets and start...

Appendix 2. Printable logbook for a TMS mapping session (one for each hemisphere).

Date: ____/____/______
PRH_ ________
T1 O<br>T4 O<br>T6 O<br>Tx O
M1 hemisphere<br><br>R O<br>L O
Hotspot_____;_______<br><br>RMT ________<br><br>M1Map (120% RMT) ______<br><br>LangMap (90% RMT) ______

8									
7									
6									
5									
4									
3									
2									
1									
0									
	0	1	2	3	4	5	6	7	8

Appendix 3. Matlab script for TMS language mapping.

```
%%%%%%%%%%%%%%%%%%%%%%%%%%%%%%%%%%%%%%%%%%%%%%%%%%%
%%%%%%%%%%%%%%%%%%%%

clear; close all; clc; % Clear everyting

%% Get Experiment Settings

debugging = 0; % Change this to one to disable all external equipment triggering

ntrials = 25;

IMApath = [pwd,'\images\'];

prompt = {'Date (DDMMYY):', ...

    'Subject ID:',...

    'Image Sequence',...

    'Targets Sequence',...

    'TMS intensity',...

    'Number of rTMS trains',...

    'Arduino Button Trigger'};

defaults = {char(datetime('now','Format','ddMMyy')),'PRH','1','1','30','1','1'};

answer = inputdlg(prompt,'Experimental setup',1,defaults);


[S.date, S.ID, S.IMAsequence, S.TARsequence, S.power, S.ntrains, S.arduino] = deal(answer{:});

S.date = str2double(S.date);

S.TARsequence = str2double(S.TARsequence);

S.IMAsequence = str2double(S.IMAsequence);

S.power = str2double(S.power);

S.ntrains = str2double(S.ntrains);

S.arduino = str2double(S.arduino);


%% Create Results Table

S.Results                                                                    =
table('Size',[ntrials,6],'VariableTypes',{'string','datetime','double','double','string','string'},...


'VariableNames',{'SubjectID','OpticalTime','IMA_Sequence','TAR_Sequence','ImageName','Vide
oName'});
```

```
S.Results(:,1) = {S.ID};
%S.Results(:,2) = {S.date};
S.Results.IMA_Sequence = S.IMAsequence*ones(ntrials,1);
S.Results.TAR_Sequence = S.TARsequence*ones(ntrials,1);
%Create results folders
if ~exist('results', 'dir')
   mkdir('results')
end
if ~exist(['results\',S.ID], 'dir')
   mkdir(['results\',S.ID])
   mkdir(['results\',S.ID,'\audios'])
end

%% Setup Arduino PushButton trigger
if S.arduino && ~debugging
   disp('Conecting to Arduino...')
   port='COM3';
   board='Uno';
   try
   a=arduino(port, board);
   configurePin(a,'D2',"DigitalInput");
   catch
      disp('Cannot connect to Arduino! Use Keybord instead')
      S.arduino = 0;
   end
end
%%  Setup parallel port
if ~debugging
 ioObj = io64;
 status = io64(ioObj);
 if(status == 0)
```

```
    disp('Parallel port object created succesfully')
 else
    disp('unable to create parallel port object')
 end
 adress = hex2dec('3FF8');
 io64(ioObj, adress, 0);
end
%% Setup Magventure
if ~debugging
   magventureObject = magventure('COM4');
   magventureObject.connect();
   magventureObject.setTrain(5,5,S.ntrains,1);
   magventureObject.arm();
   magventureObject.setAmplitude(S.power);
end
%% Load the sequence of images (from xxx folder) and convert to array
sequencePath = [IMApath,'img_set',num2str(S.IMAsequence),'\'];
IMAlist = dir([sequencePath,'*.jpg']);
IMAlist = Shuffle({IMAlist.name});
S.Results.ImageName = IMAlist';
imdata = cell(1,ntrials);
for i = 1:ntrials
   imdata_temp=imread(char([sequencePath,IMAlist{i}]));
   imdata{i} = imresize(imdata_temp,[800 600]);
end
%% Setup Psychotoolbox stuff
%Get screen paramenters and initialize
Screen('Preference', 'SkipSyncTests', 2);
screens = Screen('Screens');
screenNumber = 1;
white = WhiteIndex(screenNumber);
```

```
black = BlackIndex(screenNumber);
grey = white / 2;
[window, windowRect] = PsychImaging('OpenWindow', screenNumber, grey);
Screen('BlendFunction', window, 'GL_SRC_ALPHA', 'GL_ONE_MINUS_SRC_ALPHA');
[myWin] = get_winprops(window, windowRect, screenNumber);
% fixation parameters
fixCrossDimPix = 60; % the length of arms of fixation cross
fixCrossLine = 6; % the thickness of lines of fixation cross
allCoordsFix = [-fixCrossDimPix fixCrossDimPix 0 0;0 0 -fixCrossDimPix fixCrossDimPix];
% Make a base Rect of 200 by 250 pixels
baseRect = [0 0 150 150];
% For Ovals we set a miximum diameter up to which it is perfect for
maxDiameter = max(baseRect) * 1.01;
%HideCursor;
% Do some logging functions checks and initialize them to avoid future
% delays.
KbCheck;
WaitSecs(0.1);
GetSecs;
% Set priority for script execution to realtime priority:
priorityLevel=MaxPriority(window);
Priority(priorityLevel);
%% Initialize sound recording with low latency mode
pahandle = PsychPortAudio('Open', 1, 2, 1, 16000, 1);
s = PsychPortAudio('GetStatus', pahandle); % Get sound sampling paramenters
S.audiosrate = s.SampleRate;
S.audiodata = {};
%% Main task loop
for i = 1:ntrials
   %Draw and show white fixation cross
```

```
Screen('DrawLines', window, allCoordsFix, fixCrossLine, white, [myWin.xCenter myWin.yCenter], 2);
Screen('Flip', window);
%Transform image into a texture
tex=Screen('MakeTexture', window, imdata{i});
%Setup video/audio capture settings
moviename = ['trial_',num2str(i),'.wav'];
S.Results.VideoName(i) = moviename;
%We tell experimenter that he can now trigger machine
disp('GOOOOOOOOOOOOO!!!!')
%Wait for Arduino pushbutton trigger
ready = 0;
if S.arduino && ~debugging
  button_pressed = 0;
  while(~ready)
    [keyIsDown, secs, keyCode, deltaSecs] = KbCheck;
    button_pressed = readDigitalPin(a,'D2');
    if keyCode(KbName('t'))
      ready = 1;
    elseif keyCode(KbName('esc'))
      break
    elseif button_pressed
      ready = 1;
    end
    WaitSecs(0.01)
  end
%Or wait for keyboard pushbutton press to start trial
else
  while ~ready
    [keyIsDown, secs, keyCode, deltaSecs] = KbCheck;
    if keyCode(KbName('t'))
```

```
          ready = 1;
        elseif keyCode(KbName('esc'))
          break
        end
      end
    end
    if ~ready
      break
    end
    S.Results.OpticalTime(i) = datetime('now');
    % Empty audio buffer from previous trial and Start Capturing Video and audio
    if i > 1
      PsychPortAudio('GetAudioData', pahandle);
    end
    PsychPortAudio('GetAudioData', pahandle, 5); %Prealocate buffer of size 5secs
    PsychPortAudio('Start', pahandle, 0, 0, 1);
    %Draw image texture and show on screen
    Screen('DrawTexture', window, tex);
    Screen('Flip', window);
    %Send TTL high signal to MagPro
%     io64(ioObj, adress, 255);
    if ~debugging
    magventureObject.sendTrain()
    end
    %After 1s stop showing image and draw fixation back
    WaitSecs(1)
    Screen('DrawLines', window, allCoordsFix, fixCrossLine, white, [myWin.xCenter myWin.yCenter], 2);
    [VBLTimestamp, startrt]=Screen('Flip', window);
    %Keep recording audio for 3s
    WaitSecs(3);
```

```
    %Get audio data from buffer
    audiotemp = PsychPortAudio('GetAudioData', pahandle);
    S.audiodata = [S.audiodata audiotemp];
    % Stop capture:
    PsychPortAudio('Stop', pahandle);
end

%% Save Results, clear ports and close up
save(['results\',S.ID,'\res_struct.mat'],'S')
writetable(S.Results,['results\',S.ID,'\res_table.xls'])
audiofnames = rmmissing(S.Results.VideoName);
for i = 1:length(S.audiodata)
    audiowrite(['results\',S.ID,'\audios\',audiofnames{i}], transpose(S.audiodata{i}), S.audiosrate)
end
if S.arduino && ~debugging
    clear a
end
if ~debugging
magventureObject.disconnect();
io64(ioObj,adress,0);
clear magventureObject ioObj
end
PsychPortAudio('Close', pahandle);
sca;
```

Appendix 4. Matlab script for Euclidean distance calculation (between two points)

```
% Load the MNI coordinates from a text file
coords = dlmread('P9_T4_stim_pdf.txt');

% Load the transformation matrix from MNI to voxel space
MNI_to_voxel = spm_matrix([0 0 0 -78 -112 -70 1 1 1 0 0 0 0 0 0 1]);

% Apply the transformation matrix to convert MNI coordinates to voxel coordinates
voxels = round(MNI_to_voxel \ [coords, ones(size(coords,1),1)]');
voxels = voxels(1:3,:)';

% Calculate the Euclidean distance between all coordinates and the first one
ncoords = size(coords,1);
distances = zeros(ncoords-1, 1);
for i = 2:ncoords % start at index 2 to skip the first coordinate
   distances(i-1) = sqrt(sum((voxels(1,:) - voxels(i,:)).^2));
end

% Save the distances to a text file
dlmwrite('results_P9_T4_stim_pdf.txt', distances, 'delimiter', '\t', '-append');

% Display the distances in a column
disp(distances(:));

% Copyright 2023. ChatGPT3 & Kilian Abellaneda-Pérez, PhD
```

Appendix 5. Matlab script for Euclidean distance calculation (between two volumes).

```
% Add the SPM12 toolbox to MATLAB path
%addpath('C:\Users\lboccuni\OneDrive - INSTITUT GUTTMANN\Escritorio\MATLAB\spm12');

% Specify the paths to the first and second mask NIfTI files
mask1_path = 'D:\analisi_luglio\cluster_fMRI_analisi\P9_T4_main.nii';
mask2_path = 'D:\analisi_luglio\cluster_fMRI_analisi\P9T1.nii';

% Load the first mask NIfTI file
mask1 = spm_vol(mask1_path);

% Load the second mask NIfTI file
mask2 = spm_vol(mask2_path);

% Get the mask data from both files
mask_data1 = spm_read_vols(mask1);
mask_data2 = spm_read_vols(mask2);

% Find the voxel indices of non-zero voxels in both masks
indices1 = find(mask_data1);
indices2 = find(mask_data2);

% Convert voxel indices to 3D coordinates using the affine transformation matrix
coords1 = zeros(numel(indices1), 3);
for i = 1:numel(indices1)
    [coords1(i, 1), coords1(i, 2), coords1(i, 3)] = ind2sub(mask1.dim, indices1(i));
    coords1(i, :) = coords1(i, :) * mask1.mat(1:3, 1:3)' + mask1.mat(1:3, 4)';
end

coords2 = zeros(numel(indices2), 3);
for i = 1:numel(indices2)
```

```
    [coords2(i, 1), coords2(i, 2), coords2(i, 3)] = ind2sub(mask2.dim, indices2(i));
    coords2(i, :) = coords2(i, :) * mask2.mat(1:3, 1:3)' + mask2.mat(1:3, 4)';
end

% Calculate the squared Euclidean distances between coordinates
squared_distances = zeros(size(coords1, 1), size(coords2, 1));
for i = 1:size(coords1, 1)
    for j = 1:size(coords2, 1)
        squared_distances(i, j) = sum((coords1(i, :) - coords2(j, :)).^2);
    end
end

% Find the minimum distance and its corresponding indices
[min_distance, min_indices] = min(squared_distances(:));
[min_index1, min_index2] = ind2sub(size(squared_distances), min_indices);

% Get the MNI coordinates of the nearest points
nearest_coords1 = coords1(min_index1, :);
nearest_coords2 = coords2(min_index2, :);

% Display the minimal squared Euclidean distance
disp('Minimal Squared Euclidean Distance:');
disp(min_distance);

% Display the coordinates of the two nearest points
disp('Coordinates of Nearest Point 1 (Mask 1):');
disp(nearest_coords1);
disp('Coordinates of Nearest Point 2 (Mask 2):');
disp(nearest_coords2);

% Save the generating matrix to a file
```

```
generating_matrix_file = 'generating_matrix.mat';

generating_matrix = coords1(min_index1, :)' * coords2(min_index2, :);

save(generating_matrix_file, 'generating_matrix');

disp(['Generating matrix saved to ' generating_matrix_file]);
```
